# Supplementary material for: Protein Networks Associated with Native Metabotropic Glutamate 1 Receptors (mGlu1) in the Mouse Cerebellum
Source: Cells. 2023 May 5;12(9):1325. doi: 10.3390/cells12091325 (PMC10177021; doi:10.3390/cells12091325)
Supplement: Supplementary file 1 [file cells-12-01325-s001.zip › Figure S1.pdf]

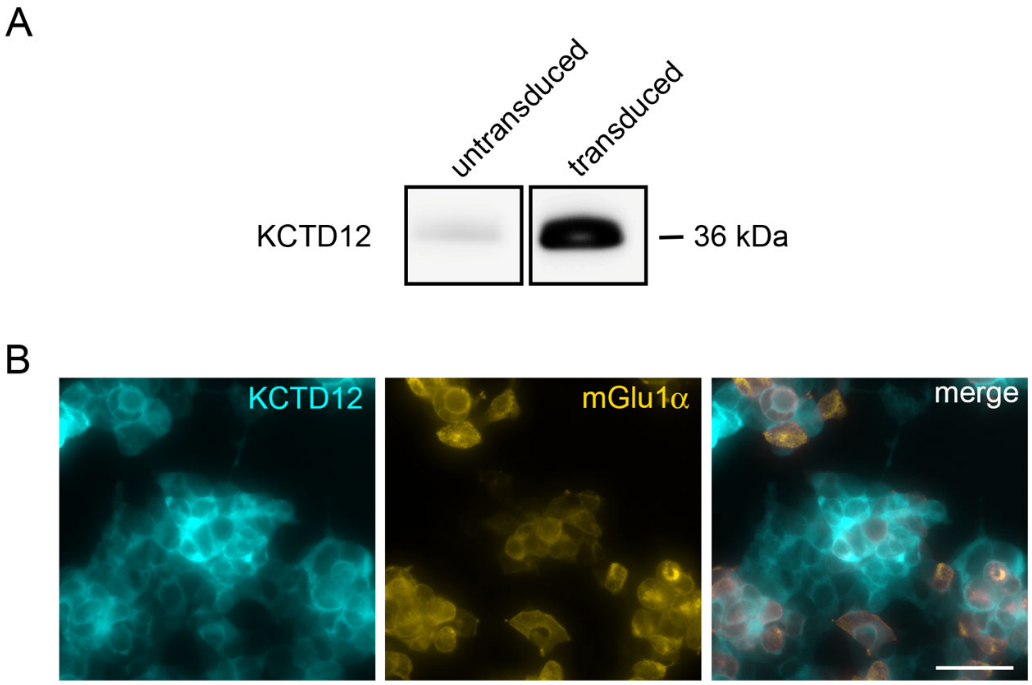

Supplementary Figure S1. Overexpression of mouse KCTD12 and mGlu1α in HEK293 cells. A: Western blot on whole-cell lysates (1μg) of untransduced and KCTD12 stably-overexpressing HEK293 cells. Native untransduced HEK293 cells show low levels of endogenous human KCTD12 expression. B: Fluorescence images of KCTD12-overexpressing HEK293 cells transiently transfected with mGlu1α. Scale bar:50 μm.
