# Supplementary material for: Protein Networks Associated with Native Metabotropic Glutamate 1 Receptors (mGlu1) in the Mouse Cerebellum
Source: Cells. 2023 May 5;12(9):1325. doi: 10.3390/cells12091325 (PMC10177021; doi:10.3390/cells12091325)
Supplement: Supplementary file 1 [file cells-12-01325-s001.zip › Table S1.pdf]

Table S1: mGlu$_1$ peptides identified by LC-MS/MS in six immunoprecipitation replicate experiments.

mGlu$_1\alpha$-specific peptides are indicated in bold and light green cells. mGlu$_1\beta$-specific peptides are indicated in italics and in light blue cells. The other peptides do not discriminate between the two isoforms

Peptide	Exp 1	Exp 2	Exp 3	Exp 4	Exp 5	Exp 6
ACDLGWWPNAELTGCEPITIR	X	X	X	X	X	
AMLDIVKR		X	X	X		
CLPDGQTLPPGR	X	X	X	X	X	X
DEVIEGYEVEANGGITIK			X			X
DSCWHSSVALEQSIEFIR	X		X	X	X	
DSLISIRDEK	X	X		X	X	X
DSLISIRDEKDGLNR	X	X				
DSVASGSSVPSSPVSESVLCTPPNVTYASVILR			X	X	X	
EEPISPPGEDDDDDSSER	X	X	X	X	X	X
EGNTEEDDLEEEEDLPAASK	X	X	X	X	X	X
ELAAQEGLCIAHSDKIYSNAGEK					X	
ELAAQEGLCIAHSDK	X	X	X	X	X	X
ENEFVQDEFTCR	X	X	X	X	X	X
GDAPGRYDIMNLQYTEANR					X	
GEVSCCWICTACK			X	X	X	
GLPPPLPQQQQQPPPQPPPQQPK	X	X	X	X	X	X
GPPVATTPPLPPHLSAEETPLFLADSVIPK	X	X	X	X	X	X
KGEVSCCWICTACK		X	X	X	X	
KLLDFLIK	X	X	X	X	X	X
KPIAGVIGPGSSSVAIQVQNLLQLFDIPQIAYSATSIDLSDK				X	X	
KPIAGVIGPGSSSVAIQVQNLLQLFDIPQIAYSATSIDLSDKTLYK					X	
LGVVGEFSLIGSDGWADR			X	X	X	
LLDFLIK	X		X	X		X
LLQEFVYER	X			X	X	X
LLVGLSSAMCYSALVTK	X		X	X	X	X
LPGHLLENPNFK	X	X	X	X	X	X
LPGHLLENPNFKK	X	X				X
LTPEDSPALTPPSPFR	X	X	X	X	X	X
MDGDVIIGALFSVHHQPPAEK	X	X	X	X	X	X
MHVGDGKLPCR				X	X	
MYIIIAKPER	X	X	X	X	X	X
NPWFPEFWQHR	X	X	X	X	X	
RQPEFSPSSQCPSAHVQL		X	X	X		
RGPPVATTPPLPPHLSAEETPLFLADSVIPK	X	X	X	X	X	
QPEFSPSSQCPSAHVQL			X	X	X	X
SAFTTSDVVR	X		X	X	X	X
SFDDYFLK	X	X		X		
SLYPPPPPPQHLQMLPLQLSTFR			X			X
SNTFLNIFR	X	X		X		
SSFVGVSGEEVWFDEK	X	X	X	X	X	X
SSFVGVSGEEVWFDEKGDAPGR	X	X	X	X	X	X

SLMDQLQGVVTNFGSGIPDFHAVLAGPGTPGNGLR			X	X	X	
SVSWSEPGGR	X					
TLYNVEEEDNTPSTHFSPPSSPSMVVHR				X	X	
TNETACNQTAVIKPLTK	X	X	X	X	X	X
VEAMFHTLDK	X	X	X	X	X	X
VVVCFCEGMTVR	X	X	X	X	X	X
YDIMNLQYTEANR	X	X	X	X	X	X
YDYVHVGTWHEGVLNIDDYK	X	X	X	X		X
