# Supplementary material for: Protein Networks Associated with Native Metabotropic Glutamate 1 Receptors (mGlu1) in the Mouse Cerebellum
Source: Cells. 2023 May 5;12(9):1325. doi: 10.3390/cells12091325 (PMC10177021; doi:10.3390/cells12091325)
Supplement: Supplementary file 1 [file cells-12-01325-s001.zip › Table S2.pdf]

Supplementary Table S2: complete list of proteins identified in immunoprecipitation experiments.
Proteins indentified in six replicate experiments are shown in bold.

Entry	Entry name	Protein names	Gene names	Organism	Length
Q9CQV8	1433B_MOUSE	14-3-3 protein beta/alpha	Ywhab	Mus musculus	246
P62259	1433E_MOUSE	14-3-3 protein epsilon	Ywhae	Mus musculus	255
P68510	1433F_MOUSE	14-3-3 protein eta	Ywhah	Mus musculus	246
**P61982**	**1433G_MOUSE**	**14-3-3 protein gamma**	**Ywhag**	**Mus musculus**	**247**
**P68254**	**1433T_MOUSE**	**14-3-3 protein theta**	**Ywhaq**	**Mus musculus**	**245**
P63101	1433Z_MOUSE	14-3-3 protein zeta/delta	Ywhaz	Mus musculus	245
Q9Z1T6	FYV1_MOUSE	1-phosphatidylinositol 3-phosphate 5-kinase	Pikfyve Fab1 Kiaa0981 Pip5k3	Mus musculus	2097
Q91UZ1	Q91UZ1_MOUSE	1-phosphatidylinositol 4,5-bisphosphate phosphodiesterase	Plcb4	Mus musculus	1175
**P16330**	**CN37_MOUSE**	**2',3'-cyclic-nucleotide 3'-phosphodiesterase**	**Cnp Cnp1**	**Mus musculus**	**420**
Q60597	ODO1_MOUSE	2-oxoglutarate dehydrogenase, mitochondrial	Ogdh Kiaa4192	Mus musculus	1023
P61922	GABT_MOUSE	4-aminobutyrate aminotransferase, mitochondrial	Abat Gabat	Mus musculus	500
P63038	CH60_MOUSE	60 kDa heat shock protein, mitochondrial	Hspd1 Hsp60	Mus musculus	573
Q8QZT1	THIL_MOUSE	Acetyl-CoA acetyltransferase, mitochondrial	Acat1	Mus musculus	424
Q5SWU9	ACACA_MOUSE	Acetyl-CoA carboxylase 1	Acaca Acac, Gm738	Mus musculus	2345
Q99KI0	ACON_MOUSE	Aconitate hydratase, mitochondrial	Aco2	Mus musculus	780
P68033	ACTC_MOUSE	Actin, alpha cardiac muscle 1	Actc1 Actc	Mus musculus	377
P60710	ACTB_MOUSE	Actin, cytoplasmic 1 (Beta-actin)	Actb	Mus musculus	375
P63260	ACTG_MOUSE	Actin, cytoplasmic 2 (Gamma-actin)	Actg1 Actg	Mus musculus	375
**P48962**	**ADT1_MOUSE**	**ADP/ATP translocase 1**	**Slc25a4 Aac1 Anc1 Ant1**	**Mus musculus**	**298**
P51881	ADT2_MOUSE	ADP/ATP translocase 2	Slc25a5 Aac2 Ant2	Mus musculus	298
P84084	ARF5_MOUSE	ADP-ribosylation factor 5	Arf5	Mus musculus	180
P07724	ALBU_MOUSE	Albumin	Alb Alb-1 Alb1	Mus musculus	608
Q91VB8	Q91VB8_MOUSE	Alpha globin 1	Hba-a1 Glnc1, haemaglobin alpha 1, haemaglobin alpha 2, Hba-a2	Mus musculus	142
P17182	ENOA_MOUSE	Alpha-enolase	Eno1 Eno-1	Mus musculus	434

P46660	AINX_MOUSE	Alpha-internexin	Ina	Mus musculus	501
Q02357	ANK1_MOUSE	Ankyrin-1	Ank1 Ank-1	Mus musculus	1862
Q8C8R3	ANK2_MOUSE	Ankyrin-2	Ank2 AnkB	Mus musculus	3898
G5E8K5	ANK3_MOUSE	Ankyrin-3	Ank3	Mus musculus	1961
P48036	ANXA5_MOUSE	Annexin A5	Anxa5 Anx5	Mus musculus	319
O35643	AP1B1_MOUSE	AP-1 complex subunit beta-1	Ap1b1 Adtb1	Mus musculus	943
Q7SIG6	ASAP2_MOUSE	Arf-GAP with SH3 domain, ANK repeat and PH domain-containing protein 2	Asap2 Ddef2, Gm1523, Gm592	Mus musculus	958
P05202	AATM_MOUSE	Aspartate aminotransferase, mitochondrial	Got2 Got-2	Mus musculus	430
Q9CQQ7	AT5F1_MOUSE	ATP synthase F(0) complex subunit B1, mitochondrial	Atp5pb Atp5f1	Mus musculus	256
Q78IK2	ATPMK_MOUSE	ATP synthase membrane subunit K, mitochondrial	Atp5mk Atp5md, Dapit, Usmg5	Mus musculus	58
P03930	ATP8_MOUSE	ATP synthase protein 8	Mtatp8 Atp8, mt-Atp8	Mus musculus	67
Q03265	ATPA_MOUSE	ATP synthase subunit alpha, mitochondrial	Atp5f1a Atp5a1	Mus musculus	553
P56480	ATPB_MOUSE	ATP synthase subunit beta, mitochondrial	Atp5f1b Atp5b	Mus musculus	529
Q9DCX2	ATP5H_MOUSE	ATP synthase subunit d, mitochondrial	Atp5pd Atp5h	Mus musculus	161
Q9D3D9	ATPD_MOUSE	ATP synthase subunit delta, mitochondrial	Atp5f1d Atp5d	Mus musculus	168
P56135	ATPK_MOUSE	ATP synthase subunit f, mitochondrial	Atp5mf Atp5j2	Mus musculus	88
**Q91VR2**	**ATPG_MOUSE**	**ATP synthase subunit gamma, mitochondrial**	**Atp5f1c Atp5c1**	**Mus musculus**	**298**
Q9DB20	ATPO_MOUSE	ATP synthase subunit O, mitochondrial	Atp5po Atp5o, D12Wsu28e	Mus musculus	213
Q9ESV0	DDX24_MOUSE	ATP-dependent RNA helicase DDX24	Ddx24	Mus musculus	857
Q99PV8	BC11B_MOUSE	B-cell lymphoma/leukemia 11B	Bcl11b Ctip2, Rit1	Mus musculus	884
P28663	SNAB_MOUSE	Beta-soluble NSF attachment protein (SNAP-beta)	Napb Snapb	Mus musculus	298
**Q6WVG3**	**KCD12_MOUSE**	**BTB/POZ domain-containing protein KCTD12**	**Kctd12 Pfet1**	**Mus musculus**	**327**
P12658	CALB1_MOUSE	Calbindin	Calb1	Mus musculus	261
P11798	KCC2A_MOUSE	Calcium/calmodulin-dependent protein kinase type II subunit alpha	Camk2a	Mus musculus	478
**P28652**	**KCC2B_MOUSE**	**Calcium/calmodulin-dependent protein kinase type II subunit beta**	**Camk2b Camk2d**	**Mus musculus**	**542**
Q6PHZ2	KCC2D_MOUSE	Calcium/calmodulin-dependent protein kinase type II subunit delta	Camk2d Kiaa4163	Mus musculus	499

Q08460	KCMA1_MOUSE	Calcium-activated potassium channel subunit alpha-1	Kcnma1 Kcnma	Mus musculus	1209
Q8BH59	CMC1_MOUSE	Calcium-binding mitochondrial carrier protein Aralar1	Slc25a12 Aralar1	Mus musculus	677
Q0VF55	Q0VF55_MOUSE	Calcium-transporting ATPase	Atp2b3	Mus musculus	1220
Q6W3E0	Q6W3E0_MOUSE	Calmodulin 5	Calm5 Scarf2	Mus musculus	140
P0DP28	CALM3_MOUSE	Calmodulin-3	Calm3 Cam3 Camc	Mus musculus	149
P35564	CALX_MOUSE	Calnexin	Canx	Mus musculus	591
Q9JKC6	CEND_MOUSE	Cell cycle exit and neuronal differentiation protein 1	Cend1 Bm88	Mus musculus	149
P60766	CDC42_MOUSE	Cell division control protein 42 homolog	Cdc42	Mus musculus	191
Q6A078	CE290_MOUSE	Centrosomal protein of 290 kDa	Cep290 Kiaa0373 Nphp6	Mus musculus	2472
Q68FD5	CLH1_MOUSE	Clathrin heavy chain 1	Cltc	Mus musculus	1675
Q60771	CLD11_MOUSE	Claudin-11	Cldn11 Osp Otm	Mus musculus	207
P45591	COF2_MOUSE	Cofilin-2	Cfl2	Mus musculus	166
E9Q6B2	CC85C_MOUSE	Coiled-coil domain-containing protein 85C	Ccdc85c	Mus musculus	420
Q9QZS0	CO4A3_MOUSE	Collagen alpha-3(IV) chain	Col4a3	Mus musculus	1669
Q02105	C1QC_MOUSE	Complement C1q subcomponent subunit C	C1qc C1qg	Mus musculus	246
P12960	CNTN1_MOUSE	Contactin-1	Cntn1	Mus musculus	1020
O54991	CNTP1_MOUSE	Contactin-associated protein 1	Cntnap1 Nrxn4	Mus musculus	1385
Q1RLL3	CPNE9_MOUSE	Copine-9	Cpne9	Mus musculus	553
Q04447	KCRB_MOUSE	Creatine kinase B-type	Ckb Ckbb	Mus musculus	381
P30275	KCRU_MOUSE	Creatine kinase U-type, mitochondrial	Ckmt1	Mus musculus	418
P97315	CSRP1_MOUSE	Cysteine and glycine-rich protein 1	Csrp1 Crp1 Csrp	Mus musculus	193
Q9CZ13	QCR1_MOUSE	Cytochrome b-c1 complex subunit 1, mitochondrial	Uqcrc1	Mus musculus	480
Q9DB77	QCR2_MOUSE	Cytochrome b-c1 complex subunit 2, mitochondrial	Uqcrc2	Mus musculus	453
Q62425	NDUA4_MOUSE	Cytochrome c oxidase subunit NDUFA4	Ndufa4	Mus musculus	82
Q9D0M3	CY1_MOUSE	Cytochrome c1, heme protein, mitochondrial	Cyc1	Mus musculus	325
Q9JHU4	DYHC1_MOUSE	Cytoplasmic dynein 1 heavy chain 1	Dync1h1 Dhc1, Dnch1, Dnchc1, Dyhc	Mus musculus	4644
Q5SQX6	CYFP2_MOUSE	Cytoplasmic FMR1-interacting protein 2	Cyfip2 Kiaa1168 Pir121	Mus musculus	1253

Q8JZM4	DNER_MOUSE	Delta and Notch-like epidermal growth factor-related receptor	Dner Bet Bret	Mus musculus	737
Q6WQJ1	DGLA_MOUSE	Diacylglycerol lipase-alpha	Dagla Kiaa0659, Nsddr	Mus musculus	1044
O08749	DLDH_MOUSE	Dihydrolipoyl dehydrogenase, mitochondrial	Dld	Mus musculus	509
Q8BMF4	ODP2_MOUSE	Dihydrolipoyllysine-residue acetyltransferase component of pyruvate dehydrogenase complex	Dlat	Mus musculus	642
O08553	DPYL2_MOUSE	Dihydropyrimidinase-related protein 2	Dpysl2 Crmp2 Ulip2	Mus musculus	572
P54103	DNJC2_MOUSE	DnaJ homolog subfamily C member 2	Dnajc2 Mida1, Zrf1	Mus musculus	621
P39053	DYN1_MOUSE	Dynamin-1	Dnm1 Dnm Kiaa4093	Mus musculus	867
P63168	DYL1_MOUSE	Dynein light chain 1, cytoplasmic	Dynll1 Dlc1, Dncl1, Dnclc1	Mus musculus	89
Q61510	TRI25_MOUSE	E3 ubiquitin/ISG15 ligase TRIM25	Trim25 Efp, Zfp147, Znf147	Mus musculus	634
P23804	MDM2_MOUSE	E3 ubiquitin-protein ligase Mdm2	Mdm2	Mus musculus	489
O88343	S4A4_MOUSE	Electrogenic sodium bicarbonate cotransporter 1	Slc4a4 Nbc1, Nbce1	Mus musculus	1079
P10126	EF1A1_MOUSE	Elongation factor 1-alpha 1	Eef1a1 Eef1a	Mus musculus	462
P62631	EF1A2_MOUSE	Elongation factor 1-alpha 2	Eef1a2 Eef1al, Stn	Mus musculus	463
Q8BFR5	EFTU_MOUSE	Elongation factor Tu, mitochondrial	Tufm	Mus musculus	452
P20029	BIP_MOUSE	Endoplasmic reticulum chaperone BiP	Hspa5 Grp78	Mus musculus	655
Q8BFZ9	ERLN2_MOUSE	Erlin-2	Erlin2 Spfh2	Mus musculus	340
Q61304	Q61304_MOUSE	Erythroid ankyrin (Fragment)	Ank1 Ank-1	Mus musculus	1098
**P56564**	**EAA1_MOUSE**	**Excitatory amino acid transporter 1**	**Slc1a3 Eaat1 Gmt1**	**Mus musculus**	**543**
P43006	EAA2_MOUSE	Excitatory amino acid transporter 2	Slc1a2 Eaat2 Glt1	Mus musculus	572
**O35544**	**EAA4_MOUSE**	**Excitatory amino acid transporter 4**	**Slc1a6 Eaat4**	**Mus musculus**	**561**
P05063	ALDOC_MOUSE	Fructose-bisphosphate aldolase C	Aldoc Aldo3 Scrg2	Mus musculus	363
P97807	FUMH_MOUSE	Fumarate hydratase, mitochondrial	Fh Fh1	Mus musculus	507
Q3UNH4	GRIN1_MOUSE	G protein-regulated inducer of neurite outgrowth 1	Gprin1	Mus musculus	932
P62812	GBRA1_MOUSE	Gamma-aminobutyric acid receptor subunit alpha-1	Gabra1 Gabra-1	Mus musculus	455
P50571	GBRB1_MOUSE	Gamma-aminobutyric acid receptor subunit beta-1	Gabrb1	Mus musculus	474
P22723	GBRG2_MOUSE	Gamma-aminobutyric acid receptor subunit gamma-2	Gabrg2	Mus musculus	474
Q9WV18	GABR1_MOUSE	Gamma-aminobutyric acid type B receptor subunit 1	Gabbr1	Mus musculus	960

Q80T41	GABR2_MOUSE	Gamma-aminobutyric acid type B receptor subunit 2	Gabbr2 Gm425, Gpr51	Mus musculus	940
Q61668	Q61668_MOUSE	Gene for histone H2a (Fragment)		Mus musculus	37
P26443	DHE3_MOUSE	Glutamate dehydrogenase 1, mitochondrial	Glud1 Glud	Mus musculus	558
Q9Z2W9	GRIA3_MOUSE	Glutamate receptor 3	Gria3 Glur3, Kiaa4184	Mus musculus	888
**Q61625**	**GRID2_MOUSE**	**Glutamate receptor ionotropic, delta-2**	**Grid2**	**Mus musculus**	**1007**
P15105	GLNA_MOUSE	Glutamine synthetase	Glul Glns	Mus musculus	373
P16858	G3P_MOUSE	Glyceraldehyde-3-phosphate dehydrogenase	Gapdh Gapd	Mus musculus	333
Q921M4	GOGA2_MOUSE	Golgin subfamily A member 2	Golga2	Mus musculus	999
Q99JG2	G37L1_MOUSE	G-protein coupled receptor 37-like 1	Gpr37l1 Etbrlp2	Mus musculus	481
B2RSH2	GNAI1_MOUSE	Guanine nucleotide-binding protein G(i) subunit alpha-1	Gnai1 Gnai-1	Mus musculus	354
P08752	GNAI2_MOUSE	Guanine nucleotide-binding protein G(i) subunit alpha-2	Gnai2 Gnai-2	Mus musculus	355
P63213	GBG2_MOUSE	Guanine nucleotide-binding protein G(I)/G(S)/G(O) subunit gamma-2	Gng2	Mus musculus	71
**P62874**	**GBB1_MOUSE**	**Guanine nucleotide-binding protein G(I)/G(S)/G(T) subunit beta-1**	**Gnb1**	**Mus musculus**	**340**
P62880	GBB2_MOUSE	Guanine nucleotide-binding protein G(I)/G(S)/G(T) subunit beta-2	Gnb2	Mus musculus	340
**P18872**	**GNAO_MOUSE**	**Guanine nucleotide-binding protein G(o) subunit alpha**	**Gnao1 Gna0 Gnao**	**Mus musculus**	**354**
P21279	GNAQ_MOUSE	Guanine nucleotide-binding protein G(q) subunit alpha	Gnaq	Mus musculus	359
P21278	GNA11_MOUSE	Guanine nucleotide-binding protein subunit alpha-11	Gna11 Gna-11	Mus musculus	359
P30677	GNA14_MOUSE	Guanine nucleotide-binding protein subunit alpha-14	Gna14 Gna-14	Mus musculus	355
P63017	HSP7C_MOUSE	Heat shock cognate 71 kDa protein	Hspa8 Hsc70, Hsc73	Mus musculus	646
P11499	HS90B_MOUSE	Heat shock protein HSP 90-beta	Hsp90ab1 Hsp84 Hsp84-1 Hspcb	Mus musculus	724
P02088	HBB1_MOUSE	Hemoglobin subunit beta-1	Hbb-b1	Mus musculus	147
Q640R3	HECAM_MOUSE	Hepatocyte cell adhesion molecule	Hepacam	Mus musculus	418
A0AUV1	A0AUV1_MOUSE	Histone H2A (Fragment)	H2ac12 Hist1h2ah	Mus musculus	127
P70696	H2B1A_MOUSE	Histone H2B type 1-A	H2bc1 Hist1h2ba, Th2b	Mus musculus	127
A1L0U3	A1L0U3_MOUSE	Histone H3 (Fragment)	Hist1h3e	Mus musculus	133
P84244	H33_MOUSE	Histone H3.3	H3-3a H3.3a H3f3a; H3-3b H3.3b H3f3b	Mus musculus	136

P62806	H4_MOUSE	Histone H4	H4c1 Hist1h4a, others	Mus musculus	103
P62806	H4_MOUSE	Histone H4	H4c1 Hist1h4a; H4c2 H4-53 Hist1h4b; H4c3 H4-12 Hist1h4c; H4c4 Hist1h4d; H4c6 Hist1h4f; H4c8 Hist1h4h; H4c9 Hist1h4i; H4c11 Hist1h4j; H4c12 Hist1h4k; Hist1h4m; H4c14 Hist2h4 Hist2h4a; H4f16 Hist4h4	Mus musculus	103
Q9Z2Y3	HOME1_MOUSE	Homer protein homolog 1 (Homer-1)	Homer1 Vesl1	Mus musculus	366
**Q99JP6**	**HOME3_MOUSE**	**Homer protein homolog 3 (Homer-3)**	**Homer3**	**Mus musculus**	**356**
**P11881**	**ITPR1_MOUSE**	**Inositol 1,4,5-trisphosphate receptor type 1**	**Itpr1 Insp3r Pcd6 Pcp1**	**Mus musculus**	**2749**
**Q7TNC9**	**I5P1_MOUSE**	**Inositol polyphosphate-5-phosphatase A**	**Inpp5a**	**Mus musculus**	**412**
Q8R4K2	IRAK4_MOUSE	Interleukin-1 receptor-associated kinase 4	Irak4	Mus musculus	459
Q9D6R2	IDH3A_MOUSE	Isocitrate dehydrogenase [NAD] subunit alpha, mitochondrial	Idh3a	Mus musculus	366
Q9JJ57	KCIP1_MOUSE	Kv channel-interacting protein 1	Kcnip1 Kchip1	Mus musculus	227
Q6PHZ8	KCIP4_MOUSE	Kv channel-interacting protein 4	Kcnip4 Calp Kchip4	Mus musculus	250
Q8BLK3	LSAMP_MOUSE	Limbic system-associated membrane protein	Lsamp	Mus musculus	34
Q9ESE1	LRBA_MOUSE	Lipopolysaccharide-responsive and beige-like anchor protein	Lrba Bgl, Lba	Mus musculus	2856
P16125	LDHB_MOUSE	L-lactate dehydrogenase B chain	Ldhb Ldh-2, Ldh2	Mus musculus	334
P14152	MDHC_MOUSE	Malate dehydrogenase, cytoplasmic	Mdh1 Mor2	Mus musculus	334
P08249	MDHM_MOUSE	Malate dehydrogenase, mitochondrial	Mdh2 Mor1	Mus musculus	338
A2AG50	MA7D2_MOUSE	MAP7 domain-containing protein 2	Map7d2 Mtap7d2	Mus musculus	781
**P97772**	**GRM1_MOUSE**	**Metabotropic glutamate receptor 1**	**Grm1 Gprc1a Mglur1**	**Mus musculus**	**1199**
Q3UVX5	GRM5_MOUSE	Metabotropic glutamate receptor 5	Grm5 Gprc1e, Mglur5	Mus musculus	1203
Q9QYR6	MAP1A_MOUSE	Microtubule-associated protein 1A (MAP-1A)	Map1a Mtap1 Mtap1a	Mus musculus	2776
P27546	MAP4_MOUSE	Microtubule-associated protein 4 (MAP-4)	Map4 Mtap4	Mus musculus	1125
Q7TSJ2	MAP6_MOUSE	Microtubule-associated protein 6 (MAP-6)	Map6 Mtap6	Mus musculus	906
Q9CR62	M2OM_MOUSE	Mitochondrial 2-oxoglutarate/malate carrier protein	Slc25a11	Mus musculus	314
P53986	MOT1_MOUSE	Monocarboxylate transporter 1	Slc16a1 Mct1	Mus musculus	493

A6H6E2	MMRN2_MOUSE	Multimerin-2	Mmrn2	Mus musculus	943
P04370	MBP_MOUSE	Myelin basic protein	Mbp Shi	Mus musculus	250
P60202	MYPR_MOUSE	Myelin proteolipid protein	Plp1 Plp	Mus musculus	277
Q61885	MOG_MOUSE	Myelin-oligodendrocyte glycoprotein	Mog	Mus musculus	246
O35682	MYADM_MOUSE	Myeloid-associated differentiation marker	Myadm Mug	Mus musculus	320
P26645	MARCS_MOUSE	Myristoylated alanine-rich C-kinase substrate	Marcks Macs	Mus musculus	309
Q91VD9	NDUS1_MOUSE	NADH-ubiquinone oxidoreductase 75 kDa subunit, mitochondrial	Ndufs1	Mus musculus	727
P13595	NCAM1_MOUSE	Neural cell adhesion molecule 1	Ncam1 Ncam	Mus musculus	1115
Q64322	NPDC1_MOUSE	Neural proliferation differentiation and control protein 1	Npdc1 Npdc-1	Mus musculus	332
Q9EPN1	NBEA_MOUSE	Neurobeachin	Nbea Lyst2	Mus musculus	2936
P19246	NFH_MOUSE	Neurofilament heavy polypeptide	Nefh Kiaa0845, Nfh	Mus musculus	1090
P08551	NFL_MOUSE	Neurofilament light polypeptide	Nefl Nf68, Nfl	Mus musculus	543
P08553	NFM_MOUSE	Neurofilament medium polypeptide	Nefm Nef3 Nfm	Mus musculus	848
Q99K10	NLGN1_MOUSE	Neuroligin-1	Nlgn1 Kiaa1070	Mus musculus	843
Q69ZK9	NLGN2_MOUSE	Neuroligin-2	Nlgn2 Kiaa1366	Mus musculus	836
P06837	NEUM_MOUSE	Neuromodulin (Axonal membrane protein GAP-43)	Gap43 Basp2	Mus musculus	227
**P35802**	**GPM6A_MOUSE**	**Neuronal membrane glycoprotein M6-a**	**Gpm6a M6a**	**Mus musculus**	**278**
Q9Z1S5	SEPT3_MOUSE	Neuronal-specific septin-3	Septin3 Sep3 Sept3	Mus musculus	350
P84075	HPCA_MOUSE	Neuron-specific calcium-binding protein hippocalcin	Hpca	Mus musculus	193
P97300	NPTN_MOUSE	Neuroplastin	Nptn Sdfr1 Sdr1	Mus musculus	397
Q9Z0J4	NOS1_MOUSE	Nitric oxide synthase, brain	Nos1	Mus musculus	1429
P70353	NFYC_MOUSE	Nuclear transcription factor Y subunit gamma	Nfyc	Mus musculus	335
B2RXT3	B2RXT3_MOUSE	Oxoglutarate dehydrogenase	Ogdhl	Mus musculus	1010
Q9D7Y7	Q9D7Y7_MOUSE	Peptidase S1 domain-containing protein	2210010C04Rik	Mus musculus	247
P99029	PRDX5_MOUSE	Peroxiredoxin-5, mitochondrial	Prdx5 Prdx6	Mus musculus	210
**Q8VEM8**	**MPCP_MOUSE**	**Phosphate carrier protein, mitochondrial**	**Slc25a3**	**Mus musculus**	**357**
Q99L43	CDS2_MOUSE	Phosphatidate cytidylyltransferase 2	Cds2	Mus musculus	444
Q9ES52	SHIP1_MOUSE	Phosphatidylinositol 3,4,5-trisphosphate 5-phosphatase 1	Inpp5d 7a33, Ship, Ship1	Mus musculus	1191

E9Q3L2	PI4KA_MOUSE	Phosphatidylinositol 4-kinase alpha	Pi4ka Pik4 Pik4ca	Mus musculus	2105
Q3T9Y0	Q3T9Y0_MOUSE	Phosphatidylinositol-4,5-bisphosphate 3-kinase	Pik3cd	Mus musculus	1047
Q7TME0	PLPR4_MOUSE	Phospholipid phosphatase-related protein type 4	Plppr4 D3Bwg0562e Kiaa0455 Lppr4 Php1 Prg1	Mus musculus	766
Q68FH0	PKP4_MOUSE	Plakophilin-4	Pkp4 Armrp	Mus musculus	1190
G5E829	AT2B1_MOUSE	Plasma membrane calcium-transporting ATPase 1	Atp2b1	Mus musculus	1220
Q9R0K7	AT2B2_MOUSE	Plasma membrane calcium-transporting ATPase 2	Atp2b2 Pmca2	Mus musculus	1198
Q9CY58	PAIRB_MOUSE	Plasminogen activator inhibitor 1 RNA-binding protein	Serbp1 Pairbp1	Mus musculus	407
P0CG50	UBC_MOUSE	Polyubiquitin-C	Ubc	Mus musculus	734
Q63959	KCNC3_MOUSE	Potassium voltage-gated channel subfamily C member 3	Kcnc3	Mus musculus	769
Q9Z0V2	KCND2_MOUSE	Potassium voltage-gated channel subfamily D member 2	Kcnd2 Kiaa1044 MNCb-7013	Mus musculus	630
Q9Z0V1	KCND3_MOUSE	Potassium voltage-gated channel subfamily D member 3	Kcnd3	Mus musculus	655
O88704	HCN1_MOUSE	Potassium/sodium hyperpolarization-activated cyclic nucleotide-gated channel 1	Hcn1 Bcng1 Hac2	Mus musculus	910
P67778	PHB1_MOUSE	Prohibitin 1	Phb1 Phb	Mus musculus	272
O35129	PHB2_MOUSE	Prohibitin-2	Phb2 Bap, Bcap37, Rea	Mus musculus	299
O35449	PRRT1_MOUSE	Proline-rich transmembrane protein 1	Prrt1 Ng5	Mus musculus	306
Q9Z1R9	Q9Z1R9_MOUSE	Protease, serine 1 (trypsin 1)	Prss1 Trygn16, trypsinogen	Mus musculus	246
Q8BQN5	FA78B_MOUSE	Protein FAM78B	Fam78b	Mus musculus	261
Q61644	PACN1_MOUSE	Protein kinase C and casein kinase substrate in neurons protein 1	Pacsin1 Pacsin	Mus musculus	441
**P63318**	**KPCG_MOUSE**	**Protein kinase C gamma type**	**Prkcg Pkcc, Pkcg, Prkcc**	**Mus musculus**	**697**
O55125	NIPS1_MOUSE	Protein NipSnap homolog 1	Nipsnap1	Mus musculus	284
Q3UH99	SHSA6_MOUSE	Protein shisa-6	Shisa6 Gm879	Mus musculus	525
Q3UUD2	SPRY3_MOUSE	Protein sprouty homolog 3 (Spry-3)	Spry3	Mus musculus	288
Q80WQ2	VAC14_MOUSE	Protein VAC14 homolog	Vac14 D8Wsu151e	Mus musculus	782
P23506	PIMT_MOUSE	Protein-L-isoaspartate(D-aspartate) O-methyltransferase (PIMT)	Pcmt1	Mus musculus	227
F8VPK8	F8VPK8_MOUSE	Protocadherin 9	Pcdh9	Mus musculus	1237
Q91XX1	Q91XX1_MOUSE	Protocadherin gamma C3	Pcdhgc3	Mus musculus	934
Q68FL4	SAHH3_MOUSE	Putative adenosylhomocysteinase 3	Ahcyl2	Mus musculus	613

P35486	ODPA_MOUSE	Pyruvate dehydrogenase E1 component subunit alpha, somatic form, mitochondrial	Pdha1 Pdha-1	Mus musculus	390
P52480	KPYM_MOUSE	Pyruvate kinase PKM	Pkm Pk3 Pkm2 Pykm	Mus musculus	531
P60764	RAC3_MOUSE	Ras-related C3 botulinum toxin substrate 3	Rac3	Mus musculus	192
Q99JI6	RAP1B_MOUSE	Ras-related protein Rap-1b	Rap1b	Mus musculus	184
O54829	RGS7_MOUSE	Regulator of G-protein signaling 7	Rgs7	Mus musculus	469
Q7M6X3	Q7M6X3_MOUSE	Regulator of sex-limitation candidate 8	Zfp759 BC028265, Rslcan8	Mus musculus	721
Q6ZQ82	RHG26_MOUSE	Rho GTPase-activating protein 26	Arhgap26 Kiaa0621	Mus musculus	814
**Q8BW86**	**ARG33_MOUSE**	**Rho guanine nucleotide exchange factor 33**	**Arhgef33 Gm941**	**Mus musculus**	**850**
Q80SW1	SAHH2_MOUSE	S-adenosylhomocysteine hydrolase-like protein 1	Ahcyl1 Irbit	Mus musculus	530
Q64518	AT2A3_MOUSE	Sarcoplasmic/endoplasmic reticulum calcium ATPase 3	Atp2a3	Mus musculus	999
Q9Z0I6	Q9Z0I6_MOUSE	Schlafen 2	Slfn2 OTTMUSCASG00059410, OTTMUSPWKG00059397	Mus musculus	378
Q9QUR8	SEM7A_MOUSE	Semaphorin-7A	Sema7a Cd108 Semal Semk1	Mus musculus	664
E9Q1G8	E9Q1G8_MOUSE	Septin	Septin7 Sept7	Mus musculus	437
Q8C1B7	SEP11_MOUSE	Septin-11	Septin11 D5Ertd606e Sept11	Mus musculus	431
P28661	SEPT4_MOUSE	Septin-4	Septin4 Bh5 Gm11492 Pnutl2 Sep4 Sept4	Mus musculus	478
Q9Z2Q6	SEPT5_MOUSE	Septin-5	Septin5 Pnutl1, Sept5	Mus musculus	369
Q9R1T4	SEPT6_MOUSE	Septin-6	Septin6 Kiaa0128 Sept6	Mus musculus	434
O55131	SEPT7_MOUSE	Septin-7	Septin7 Cdc10, Sept7	Mus musculus	436
Q6P1F6	2ABA_MOUSE	Serine/threonine-protein phosphatase 2A 55 kDa regulatory subunit B alpha isoform	Ppp2r2a	Mus musculus	447
P63328	PP2BA_MOUSE	Serine/threonine-protein phosphatase 2B catalytic subunit alpha isoform	Ppp3ca Calna	Mus musculus	521
P48453	PP2BB_MOUSE	Serine/threonine-protein phosphatase 2B catalytic subunit beta isoform	Ppp3cb Calnb	Mus musculus	525
P48455	PP2BC_MOUSE	Serine/threonine-protein phosphatase 2B catalytic subunit gamma isoform	Ppp3cc Calnc	Mus musculus	513
P63087	PP1G_MOUSE	Serine/threonine-protein phosphatase PP1-gamma catalytic subunit	Ppp1cc	Mus musculus	323
D3YZU1	SHAN1_MOUSE	SH3 and multiple ankyrin repeat domains protein 1 (Shank1)	Shank1	Mus musculus	2167

Q80Z38	SHAN2_MOUSE	SH3 and multiple ankyrin repeat domains protein 2 (Shank2)	Shank2 Cortbp1 Kiaa1022	Mus musculus	1476
B1ATV3	B1ATV3_MOUSE	Short transient receptor potential channel 3	Trpc3 CT7-314H19.1-001	Mus musculus	910
Q61143	TRPC6_MOUSE	Short transient receptor potential channel 6	Trpc6 Trp6, Trrp6	Mus musculus	930
Q91V61	SFXN3_MOUSE	Sideroflexin-3	Sfxn3	Mus musculus	321
Q925N0	SFXN5_MOUSE	Sideroflexin-5	Sfxn5	Mus musculus	342
Q14BI1	Q14BI1_MOUSE	Slc24a2 protein	Slc24a2	Mus musculus	711
P31648	SC6A1_MOUSE	Sodium- and chloride-dependent GABA transporter 1	Slc6a1 Gabt1, Gat-1, Gat1	Mus musculus	599
P31650	S6A11_MOUSE	Sodium- and chloride-dependent GABA transporter 3 (GAT-3)	Slc6a11 Gabt3 Gabt4 Gat-4 Gat3 Gat4	Mus musculus	627
Q62467	Q62467_MOUSE	Sodium channel protein	Scn7a	Mus musculus	1681
P70414	NAC1_MOUSE	Sodium/calcium exchanger 1	Slc8a1 Ncx	Mus musculus	970
Q8K596	NAC2_MOUSE	Sodium/calcium exchanger 2	Slc8a2 Ncx2	Mus musculus	921
Q8VDN2	AT1A1_MOUSE	Sodium/potassium-transporting ATPase subunit alpha-1	Atp1a1	Mus musculus	1023
**Q6PIE5**	**AT1A2_MOUSE**	**Sodium/potassium-transporting ATPase subunit alpha-2**	**Atp1a2**	**Mus musculus**	**1020**
**Q6PIC6**	**AT1A3_MOUSE**	**Sodium/potassium-transporting ATPase subunit alpha-3**	**Atp1a3**	**Mus musculus**	**1013**
**P14094**	**AT1B1_MOUSE**	**Sodium/potassium-transporting ATPase subunit beta-1**	**Atp1b1 Atp4b**	**Mus musculus**	**304**
P14231	AT1B2_MOUSE	Sodium/potassium-transporting ATPase subunit beta-2	Atp1b2	Mus musculus	290
Q61609	S20A1_MOUSE	Sodium-dependent phosphate transporter 1	Slc20a1 Glvr1 Pit1	Mus musculus	681
Q5DTL9	S4A10_MOUSE	Sodium-driven chloride bicarbonate exchanger	Slc4a10 Kiaa4136 Ncbe	Mus musculus	1118
P55012	S12A2_MOUSE	Solute carrier family 12 member 2	Slc12a2 Nkcc1	Mus musculus	1205
**Q91V14**	**S12A5_MOUSE**	**Solute carrier family 12 member 5**	**Slc12a5 Kcc2 Kiaa1176**	**Mus musculus**	**1138**
P16546	SPTN1_MOUSE	Spectrin alpha chain, non-erythrocytic 1	Sptan1 Spna2, Spta2	Mus musculus	2472
Q68FG2	Q68FG2_MOUSE	Spectrin beta chain	Sptbn2 Spnb3	Mus musculus	2388
Q62261	SPTB2_MOUSE	Spectrin beta chain, non-erythrocytic	Sptbn1 Elf, Spnb-2, Spnb2, Sptb2	Mus musculus	2363
P38647	GRP75_MOUSE	Stress-70 protein, mitochondrial (75 kDa glucose-regulated protein)	Hspa9 Grp75 Hsp74 Hspa9a	Mus musculus	679
Q8K2B3	SDHA_MOUSE	Succinate dehydrogenase [ubiquinone] flavoprotein subunit, mitochondrial	Sdha	Mus musculus	664
Q9WUM5	SUCA_MOUSE	Succinate--CoA ligase [ADP/GDP-forming] subunit alpha, mitochondrial	Suclg1	Mus musculus	346

A2ANU3	SYNG1_MOUSE	Synapse differentiation-inducing gene protein 1	Syndig1 Tmem90b	Mus musculus	258
O88935	SYN1_MOUSE	Synapsin-1	Syn1 Syn-1	Mus musculus	706
Q9JIS5	SV2A_MOUSE	Synaptic vesicle glycoprotein 2A	Sv2a Kiaa0736 Sv2	Mus musculus	742
P60879	SNP25_MOUSE	Synaptosomal-associated protein 25	Snap25 Snap	Mus musculus	206
P46096	SYT1_MOUSE	Synaptotagmin-1	Syt1	Mus musculus	421
**P46097**	**SYT2_MOUSE**	**Synaptotagmin-2**	**Syt2**	**Mus musculus**	**422**
Q99N50	SYTL2_MOUSE	Synaptotagmin-like protein 2	Sytl2 Slp2	Mus musculus	950
Q8BVI5	STX16_MOUSE	Syntaxin-16	Stx16	Mus musculus	326
P61264	STX1B_MOUSE	Syntaxin-1B	Stx1b Stx1b1 Stx1b2	Mus musculus	288
O08599	STXB1_MOUSE	Syntaxin-binding protein 1	Stxbp1	Mus musculus	594
Q9Z1A9	TBCD8_MOUSE	TBC1 domain family member 8	Tbc1d8 Hblp1 Vrp	Mus musculus	1134
P01831	THY1_MOUSE	Thy-1 membrane glycoprotein	Thy1 Thy-1	Mus musculus	162
Q9QUI0	RHOA_MOUSE	Transforming protein RhoA	Rhoa Arha, Arha2	Mus musculus	193
Q01853	TERA_MOUSE	Transitional endoplasmic reticulum ATPase	Vcp	Mus musculus	806
Q9CQG6	TM147_MOUSE	Transmembrane protein 147	Tmem147	Mus musculus	224
P17751	TPIS_MOUSE	Triosephosphate isomerase	Tpi1	Mus musculus	249
Q8BNV1	TRM2A_MOUSE	tRNA (uracil-5-)-methyltransferase homolog A	Trmt2a Htf9-c Htf9c	Mus musculus	574
P68369	TBA1A_MOUSE	Tubulin alpha-1A chain	Tuba1a Tuba1	Mus musculus	451
P68373	TBA1C_MOUSE	Tubulin alpha-1C chain	Tuba1c Tuba6	Mus musculus	449
P05214	TBA3_MOUSE	Tubulin alpha-3 chain	Tuba3a Tuba3; Tuba3b Tuba7	Mus musculus	450
P68368	TBA4A_MOUSE	Tubulin alpha-4A chain	Tuba4a Tuba4	Mus musculus	448
Q9CVR0	Q9CVR0_MOUSE	Tubulin beta chain (Fragment)	Tubb4b Tubb2c Tubb2c1	Mus musculus	291
Q9CWF2	TBB2B_MOUSE	Tubulin beta-2B chain	Tubb2b	Mus musculus	445
Q9ERD7	TBB3_MOUSE	Tubulin beta-3 chain	Tubb3	Mus musculus	450
Q9D6F9	TBB4A_MOUSE	Tubulin beta-4A chain	Tubb4a Tubb4	Mus musculus	444
P68372	TBB4B_MOUSE	Tubulin beta-4B chain	Tubb4b Tubb2c	Mus musculus	445
P99024	TBB5_MOUSE	Tubulin beta-5 chain	Tubb5	Mus musculus	444
Q922F4	TBB6_MOUSE	Tubulin beta-6 chain	Tubb6	Mus musculus	447

Q8CIV8	TBCE_MOUSE	Tubulin-specific chaperone E	Tbce	Mus musculus	524
P63024	VAMP3_MOUSE	Vesicle-associated membrane protein 3 (VAMP-3)	Vamp3 Syb3	Mus musculus	103
P46460	NSF_MOUSE	Vesicle-fusing ATPase	Nsf Skd2	Mus musculus	744
O08547	SC22B_MOUSE	Vesicle-trafficking protein SEC22b	Sec22b Sec22l1	Mus musculus	215
P62761	VISL1_MOUSE	Visinin-like protein 1	Vsnl1 Visl1	Mus musculus	191
Q60932	VDAC1_MOUSE	Voltage-dependent anion-selective channel protein 1 (VDAC-1)	Vdac1 Vdac5	Mus musculus	296
Q60930	VDAC2_MOUSE	Voltage-dependent anion-selective channel protein 2 (VDAC-2)	Vdac2 Vdac6	Mus musculus	295
P62956	CCG7_MOUSE	Voltage-dependent calcium channel gamma-7 subunit	Cacng7	Mus musculus	275
Q6PHS9	CA2D2_MOUSE	Voltage-dependent calcium channel subunit alpha-2/delta-2	Cacna2d2 Kiaa0558	Mus musculus	1154
Q8R0S4	CACB4_MOUSE	Voltage-dependent L-type calcium channel subunit beta-4	Cacnb4 Cacnlb4	Mus musculus	519
P97445	CAC1A_MOUSE	Voltage-dependent P/Q-type calcium channel subunit alpha-1A	Cacna1a Caca1a, Cach4, Cacn3, Cacnl1a4, Ccha1a	Mus musculus	2368
Q5SUG4	Q5SUG4_MOUSE	Voltage-dependent T-type calcium channel subunit alpha	Cacna1g	Mus musculus	2295
O88427	CAC1H_MOUSE	Voltage-dependent T-type calcium channel subunit alpha-1H	Cacna1h Kiaa1120	Mus musculus	2365
P62482	KCAB2_MOUSE	Voltage-gated potassium channel subunit beta-2	Kcnab2 Ckbeta2, I2rf5, Kcnb3	Mus musculus	367
Q9Z1G4	VPP1_MOUSE	V-type proton ATPase 116 kDa subunit a 1	Atp6v0a1 Atp6n1	Mus musculus	839
P50516	VATA_MOUSE	V-type proton ATPase catalytic subunit A	Atp6v1a Atp6a1 Atp6a2 Atp6v1a1	Mus musculus	617
P62814	VATB2_MOUSE	V-type proton ATPase subunit B, brain isoform	Atp6v1b2 Atp6b2, Vat2	Mus musculus	511
P51863	VA0D1_MOUSE	V-type proton ATPase subunit d 1	Atp6v0d1 Atp6d	Mus musculus	351
