# Supplementary material for: Protein Networks Associated with Native Metabotropic Glutamate 1 Receptors (mGlu1) in the Mouse Cerebellum
Source: Cells. 2023 May 5;12(9):1325. doi: 10.3390/cells12091325 (PMC10177021; doi:10.3390/cells12091325)
Supplement: Supplementary file 1 [file cells-12-01325-s001.zip › Table S3.pdf]

Supplementary Table S3: list of proteins identified in 4 out of 6 immunoprecipitation replicate experiments

Proteins identified in six replicate experiments are shown in bold.

Entry	Entry name	Protein names	Gene names	Organism	Length
P68510	1433F_MOUSE	14-3-3 protein eta	Ywhah	Mus musculus	246
**P61982**	**1433G_MOUSE**	**14-3-3 protein gamma**	**Ywhag**	**Mus musculus**	**247**
**P68254**	**1433T_MOUSE**	**14-3-3 protein theta**	**Ywhaq**	**Mus musculus**	**245**
**P16330**	**CN37_MOUSE**	**2',3'-cyclic-nucleotide 3'-phosphodiesterase**	**Cnp Cnp1**	**Mus musculus**	**420**
**P48962**	**ADT1_MOUSE**	**ADP/ATP translocase 1**	**Slc25a4 Aac1 Anc1 Ant1**	**Mus musculus**	**298**
P51881	ADT2_MOUSE	ADP/ATP translocase 2	Slc25a5 Aac2 Ant2	Mus musculus	298
**Q91VR2**	**ATPG_MOUSE**	**ATP synthase subunit gamma, mitochondrial**	**Atp5f1c Atp5c1**	**Mus musculus**	**298**
**Q6WVG3**	**KCD12_MOUSE**	**BTB/POZ domain-containing protein KCTD12**	**Kctd12 Pfet1**	**Mus musculus**	**327**
**P28652**	**KCC2B_MOUSE**	**Calcium/calmodulin-dependent protein kinase type II subunit beta**	**Camk2b Camk2d**	**Mus musculus**	**542**
Q0VF55	Q0VF55_MOUSE	Calcium-transporting ATPase	Atp2b3	Mus musculus	1220
Q1RLL3	CPNE9_MOUSE	Copine-9	Cpne9	Mus musculus	553
Q62425	NDUA4_MOUSE	Cytochrome c oxidase subunit NDUFA4	Ndufa4	Mus musculus	82
O08553	DPYL2_MOUSE	Dihydropyrimidinase-related protein 2	Dpysl2 Crmp2 Ulip2	Mus musculus	572
**P56564**	**EAA1_MOUSE**	**Excitatory amino acid transporter 1**	**Slc1a3 Eaat1 Gmt1**	**Mus musculus**	**543**
P43006	EAA2_MOUSE	Excitatory amino acid transporter 2	Slc1a2 Eaat2 Glt1	Mus musculus	572
**O35544**	**EAA4_MOUSE**	**Excitatory amino acid transporter 4**	**Slc1a6 Eaat4**	**Mus musculus**	**561**
**Q61625**	**GRID2_MOUSE**	**Glutamate receptor ionotropic, delta-2**	**Grid2**	**Mus musculus**	**1007**
P16858	G3P_MOUSE	Glyceraldehyde-3-phosphate dehydrogenase	Gapdh Gapd	Mus musculus	333
**P62874**	**GBB1_MOUSE**	**Guanine nucleotide-binding protein G(I)/G(S)/G(T) subunit beta-1**	**Gnb1**	**Mus musculus**	**340**
P62880	GBB2_MOUSE	Guanine nucleotide-binding protein G(I)/G(S)/G(T) subunit beta-2	Gnb2	Mus musculus	340
**P18872**	**GNAO_MOUSE**	**Guanine nucleotide-binding protein G(o) subunit alpha**	**Gnao1 Gna0 Gnao**	**Mus musculus**	**354**
P21279	GNAQ_MOUSE	Guanine nucleotide-binding protein G(q) subunit alpha	Gnaq	Mus musculus	359
**Q99JP6**	**HOME3_MOUSE**	**Homer protein homolog 3 (Homer-3)**	**Homer3**	**Mus musculus**	**356**
**P11881**	**ITPR1_MOUSE**	**Inositol 1,4,5-trisphosphate receptor type 1**	**Itpr1 Insp3r Pcd6 Pcp1**	**Mus musculus**	**2749**
**Q7TNC9**	**I5P1_MOUSE**	**Inositol polyphosphate-5-phosphatase A**	**Inpp5a**	**Mus musculus**	**412**
**P97772**	**GRM1_MOUSE**	**Metabotropic glutamate receptor 1**	**Grm1 Gprc1a Mglur1**	**Mus musculus**	**1199**

**P35802**	**GPM6A_MOUSE**	**Neuronal membrane glycoprotein M6-a**	**Gpm6a M6a**	**Mus musculus**	**278**
P99029	PRDX5_MOUSE	Peroxiredoxin-5, mitochondrial	Prdx5 Prdx6	Mus musculus	210
**Q8VEM8**	**MPCP_MOUSE**	**Phosphate carrier protein, mitochondrial**	**Slc25a3**	**Mus musculus**	**357**
Q9ES52	SHIP1_MOUSE	Phosphatidylinositol 3,4,5-trisphosphate 5-phosphatase 1	Inpp5d 7a33, Ship, Ship1	Mus musculus	1191
G5E829	AT2B1_MOUSE	Plasma membrane calcium-transporting ATPase 1	Atp2b1	Mus musculus	1220
O35449	PRRT1_MOUSE	Proline-rich transmembrane protein 1	Prrt1 Ng5	Mus musculus	306
**P63318**	**KPCG_MOUSE**	**Protein kinase C gamma type**	**Prkcg Pkcc, Pkcg, Prkcc**	**Mus musculus**	**697**
P35486	ODPA_MOUSE	Pyruvate dehydrogenase E1 component subunit alpha, somatic form, mitochondrial	Pdha1 Pdha-1	Mus musculus	390
**Q8BW86**	**ARG33_MOUSE**	**Rho guanine nucleotide exchange factor 33**	**Arhgef33 Gm941**	**Mus musculus**	**850**
Q64518	AT2A3_MOUSE	Sarcoplasmic/endoplasmic reticulum calcium ATPase 3	Atp2a3	Mus musculus	999
B1ATV3	B1ATV3_MOUSE	Short transient receptor potential channel 3	Trpc3 CT7-314H19.1-001	Mus musculus	910
**P31648**	**SC6A1_MOUSE**	**Sodium- and chloride-dependent GABA transporter 1**	**Slc6a1 Gabt1, Gat-1, Gat1**	**Mus musculus**	**599**
**Q6PIE5**	**AT1A2_MOUSE**	**Sodium/potassium-transporting ATPase subunit alpha-2**	**Atp1a2**	**Mus musculus**	**1020**
**Q6PIC6**	**AT1A3_MOUSE**	**Sodium/potassium-transporting ATPase subunit alpha-3**	**Atp1a3**	**Mus musculus**	**1013**
**P14094**	**AT1B1_MOUSE**	**Sodium/potassium-transporting ATPase subunit beta-1**	**Atp1b1 Atp4b**	**Mus musculus**	**304**
**Q91V14**	**S12A5_MOUSE**	**Solute carrier family 12 member 5**	**Slc12a5 Kcc2 Kiaa1176**	**Mus musculus**	**1138**
**P46097**	**SYT2_MOUSE**	**Synaptotagmin-2**	**Syt2**	**Mus musculus**	**422**
P61264	STX1B_MOUSE	Syntaxin-1B	Stx1b Stx1b1 Stx1b2	Mus musculus	288
P01831	THY1_MOUSE	Thy-1 membrane glycoprotein	Thy1 Thy-1	Mus musculus	162
P68369	TBA1A_MOUSE	Tubulin alpha-1A chain	Tuba1a Tuba1	Mus musculus	451
Q60932	VDAC1_MOUSE	Voltage-dependent anion-selective channel protein 1 (VDAC-1)	Vdac1 Vdac5	Mus musculus	296
