# Supplementary material for: Protein Networks Associated with Native Metabotropic Glutamate 1 Receptors (mGlu1) in the Mouse Cerebellum
Source: Cells. 2023 May 5;12(9):1325. doi: 10.3390/cells12091325 (PMC10177021; doi:10.3390/cells12091325)
Supplement: Supplementary file 1 [file cells-12-01325-s001.zip › Table S4.pdf]

Supplementary Table S4: list of nodes in the constructed network.

Uniprot ID	Degree	BetweennessCentrality	function	hgnc	Human Readable Label	mgd	NCBI ID	protein name	refseq	Taxonomy ID	uniprot
P14094	124	0.3361077165052403	transporter	Atp1b1	ATP1B1	Atp1b1	NP_033851.1	Sodium/potassium-transporting ATPase subunit beta-1	NP_033851	10090	Atp4b | AT1B1_MOUSE | Atp1b1 | Sodium/potassium-dependent ATPase subunit beta-1 | P14094 | ATP1B1
P68254	83	0.18682280687152655	adapter protein	Ywhaq	1433T_MOUSE	Ywhaq	NP_035869.1	14-3-3 protein theta	NP_035869	10090	1433T_RAT | YWHAQ | P68254 | 1433T_MOUSE
Q61625	57	0.16001714825525085	receptor	Grid2	GRID2	Grid2	NP_032193.1	Glutamate receptor ionotropic, delta-2	NP_032193	10090	GRID2 | Q61625 | GRID2_MOUSE
P11881	56	0.13729652374789117	channel	Itpr1	ITPR1	Itpr1	NP_034715.3	Inositol 1,4,5-trisphosphate receptor type 1	NP_034715	10090	ITPR1 | ITPR1_MOUSE | P11881
P62874	56	0.11240173375428424	G protein	Gnb1	GBB1_MOUSE	Gnb1	NP_001153488.1	Guanine nucleotide-binding protein G(I)/G(S)/G(T) subunit beta-1	NP_001153488 | NP_001153489 | NP_032168 | XP_017175466	10090	GBB1_MOUSE | Q3TQ70_MOUSE | GBB1_HUMAN | GNB1 | GBB1_CANLF | GBB1_BOVIN | P62874
P48962	49	0.1128998818290124	transporter	Slc25a4	ADT1_MOUSE	Slc25a4	NP_031476.3	ADP/ATP translocase 1	NP_031476	10090	ADT1_MOUSE | P48962 | SLC25A4
P61982	40	0.07186521534746794	adapter protein		YWHAG	Ywhag	NP_061359.2	14-3-3 protein gamma	NP_061359	10090	P61982 | YWHAG
A4QPG1	39	0.10140222041480593			Grid2					10090	A4QPG1 | Grid2
P35802	31	0.06635542584265702	membrane protein	Gpm6a	GPM6A	Gpm6a	NP_705809.1	Neuronal membrane glycoprotein M6-a	NP_001240685 | NP_001240683 | NP_705809	10090	GPM6A | Gpm6a | P35802 | M6a | GPM6A_MOUSE
Q66JR6	31	0.04957039235097093			14-3-3 protein tau					10090	Q3UJN5 | 14-3-3 protein tau | Q66JR6 | Q3TW69 | Ywhaq | Q5SP76 | P35216 | Q5U423
P28652	30	0.05782591730136208	kinase	Camk2b	KCC2B_MOUSE	Camk2b	NP_001167524.1	Calcium/calmodulin-dependent protein kinase type II subunit beta	NP_001167524 | NP_031621 | NP_001167525	10090	CAMK2B | P28652 | KCC2B_MOUSE
Q8VEM8	29	0.04764486119423986	transporter	Slc25a3	SLC25A3	Slc25a3	NP_598429.1	Phosphate carrier protein, mitochondrial	NP_598429	10090	SLC25A3 | Q8VEM8 | MPCP_MOUSE
P63318	26	0.03422663860689253	kinase	Prkcg	PRKCG	Prkcg	NP_035232.1	Protein kinase C gamma type	NP_001278363 | NP_035232	10090	PRKCG | KPCG_MOUSE | P63318
Q91VR2	24	0.04983414566712905	enzyme	Atp5c1	ATP5C1	Atp5c1	NP_065640.2	ATP synthase subunit gamma, mitochondrial	NP_065640 | NP_001106209	10090	ATPG_MOUSE | ATP synthase F1 subunit gamma | Atp5c1 | ATP5C1 | Atp5f1c | Q91VR2 | F-ATPase gamma subunit
Q5SVH9	24	0.06592777122935567			Camk2d					10090	Camk2d | Q5SVH9 | Camk2b
Q9UN99	24	0.04154497544237766			Ywhag					10090	Q9UN99 | O70457 | P35214 | Q9UDP2 | Q3UFD6 | Ywhag | Q4FK44
P97772	21	0.02920451277506344	receptor	Grm1	GRM1	Grm1	NP_058672.1	Metabotropic glutamate receptor 1	XP_006512612 | NP_001107805 | NP_058672	10090	GRM1_MOUSE | P97772 | GRM1
Q6PIC6	21	0.03421047167845455	transporter	Atp1a3	ATP1A3	Atp1a3	NP_001361556.1	Sodium/potassium-transporting ATPase subunit alpha-3	NP_659170 | NP_001277398	10090	Q6PIC6 | Atp1a3 | ATP1A3 | AT1A3_MOUSE | Sodium pump subunit alpha-3 | Na(+)/K(+) ATPase alpha(III) subunit
Q6PIE5	20	0.037454243245629563	transporter	Atp1a2	ATP1A2	Atp1a2	NP_848492.1	Sodium/potassium-transporting ATPase subunit alpha-2	NP_848492	10090	AT1A2_MOUSE | Q6PIE5 | ATP1A2
P18872	20	0.027446395721940433	G protein	Gnao1	GNAO1	Gnao1	NP_034438.1	Guanine nucleotide-binding protein G(o) subunit alpha	NP_034438 | NP_001106855	10090	GNAO1 | GNAO_MOUSE | Q543S2_MOUSE | P18872
P05697	20	0.026579604107885048			Prkcg					10090	Prkcg | Prkcc | Pkcg | P05697 | Pkcc
P16330	16	0.024310272360282995	enzyme	Cnp	CNP	Cnp	NP_001139790.1	2',3'-cyclic-nucleotide 3'-phosphodiesterase	NP_001139790 | NP_034053	10090	P16330 | CNP | CN37_MOUSE
Q99LG5	16	0.03224664768299682			Pcd6					10090	P20943 | Pcd6 | Pcp1 | Insp3r | Protein PCD-6 | IP3 receptor isoform 1 | Purkinje cell protein 1 | Type 1 inositol 1,4,5-trisphosphate receptor | Inositol 1,4,5-trisphosphate-binding protein P400 | Q99LG5 | Itpr1
P62259	15	0.08543085316441744		Ywhae	YWHAE	Ywhae			NP_033562	10090	YWHAE | 1433E_BOVIN | 1433E_SHEEP | 1433E_MOUSE | 1433E_HUMAN | 1433E_RAT | P62259
Q9CQV8	15	0.09203087261446743		Ywhab	YWHAB				NP_061223	10090	Q9CQV8 | YWHAB | 1433B_MOUSE
Q99JP6	14	0.019394356915620768	scaffolding protein	Homer3	HOME3_MOUSE	Homer3	NP_001139625.1	Homer protein homolog 3 (Homer-3)	NP_036114 | NP_001139625	10090	HOME3_MOUSE | Q99JP6 | HOMER3
Q62108	13	0.06368238161233371		Dlg4	DLG4	Dlg4			NP_001103222 | NP_031890	10090	DLG4_MOUSE | Q62108 | DLG4
Q80V62	12	0.04890034508147782		Fancd2	FACD2_MOUSE				NP_001028416	10090	FACD2_MOUSE | FANCD2 | Q80V62
P63101	12	0.05654219937747675		Ywhaz	1433Z_RAT	Ywhaz			NP_001240735 | NP_001240734 | NP_035870 | NP_001240736 | XP_011243654	10090	P63101 | 1433Z_RAT | 1433Z_MOUSE | YWHAZ
Q91WJ1	12	0.0614957538274458			Psd95					10090	Psd95 | Dlg4 | Q91WJ1 | Dlgh4 | Q5NCV7 | Q5NCV6 | Postsynaptic density protein 95 | Synapse-associated protein 90 | Q5NCV5
F6SEU4	11	0.05120158299565012		Syngap1	SYNGAP1				NP_001268420	10090	F6SEU4 | SYNGAP1 | Neuronal RasGAP | Syngap1 | SYGP1_MOUSE | Synaptic Ras GTPase-activating protein 1
Q62164	11	0.023032094450811336			ADP,ATP carrier protein 1					10090	ADP,ATP carrier protein 1 | ADP,ATP carrier protein, heart/skeletal muscle isoform T1 | Aac1 | Anc1 | Ant1 | Q62164 | Adenine nucleotide translocator 1 | Slc25a4 | Solute carrier family 25 member 4
S6CCV8	10	0.01748348168289159			Shank3					10090	F8S0X5 | F8S0X6 | Shank3 | F8S0X3 | Kiaa1650 | F8S0X2 | S6BMD3 | SPANK-2 | F8S0X0 | Proline-rich synapse-associated protein 2 | Prosap2 | Q69ZD8 | S6CCV8 | Q9JJZ3
Q923F3	10	0.01643456624738367			Cnp					10090	Q61424 | Cnp | Q923F3 | Q91V42 | Cnp1 | Q8C7C9
Q542V7	9	0.008226264907979721			Slc25a3					10090	Slc25a3 | Q542V7 | Solute carrier family 25 member 3 | Phosphate transport protein
Q9Z215	9	0.008445458296789572			Homer3					10090	Q9Z215 | D3Z6N9 | Homer3
P04697	9	0.005191142048730618			Gnb1					10090	P04697 | P04901 | Gnb1 | Transducin beta chain 1
P56564	8	0.010447592798163239	transporter	Slc1a3	SLC1A3	Slc1a3	NP_683740.1	Excitatory amino acid transporter 1	NP_683740	10090	P56564 | SLC1A3 | EAA1_MOUSE
Q01097	8	0.014044325647096225			GRIN2B					10090	GRIN2B | Q01097
P83510	8	0.016100462421150534			Kiaa0551					10090	Kiaa0551 | P83510 | TNIK | Tnik
Q6R891	7	0.020968662830087383		Ppp1r9b	NEB2_MOUSE				NP_758465	10090	Q6R891 | NEB2_MOUSE
Q80UZ8	7	0.008114976440219693			Atp1a2					10090	Atp1a2 | Na(+)/K(+) ATPase alpha(+) subunit | Sodium pump subunit alpha-2 | Q80UZ8
P18873	7	0.0054576801485057965			Gnao					10090	Gnao | Gnao1 | Gna0 | P18873
P46097	6	0.009627346084531481	synaptic protein	Syt2	SYT2	Syt2	NP_001342655.1	Synaptotagmin-2	NP_033333	10090	SYT2 | P46097 | SYT2_MOUSE
Q91V14	6	0.007569430121122557	transporter	Slc12a5	SLC12A5	Slc12a5	NP_001342409.1	Solute carrier family 12 member 5	NP_065066	10090	Q91V14 | SLC12A5 | S12A5_MOUSE
P63044	6	0.004973574025165796		Vamp2	VAMP2	Vamp2			NP_033523	10090	VAMP2_RAT | Q8CHR4_MOUSE | P63044 | VAMP2_MOUSE | VAMP2
Q3UHD9	6	0.008960857557498024			AGAP2					10090	Q3UHD9 | AGAP2
P35922	6	0.007498716510140642			FMR1					10090	Fmr-1 | Fragile X mental retardation protein 1 homolog | FMR1 | Fmr1 | P35922
Q9D415	6	0.008196007964519005			DLGAP1					10090	Q9D415 | DLGAP1
Q4ACU6	6	0.01036095969513773			SHAN3_MOUSE	Shank3			NP_067398	10090	SHAN3_MOUSE | Q4ACU6 | SHANK3
P51637	6	0.03437269993244297		Cav3	CAV3	Cav3			NP_031643	10090	M-caveolin | CAV3 | Cav3 | P51637 | CAV3_MOUSE
ENSMUSG00000029104	6	0.00580167347724501			ENSMUSG00000029104	Htt			NP_034544	10090	
Q64357	6	0.009402193889132679			Syb2					10090	Syb2 | Synaptobrevin-2 | Vamp2 | Q64357
Q9EPV6	6	0.006621872937748263			Mglur1					10090	Mglur1 | Q6AXG4 | Gprc1a | Grm1 | Q9EPV6
Q8C0G0	6	0.0051788668332182625			SAP90/PSD-95-associated protein 1					10090	Guanylate kinase-associated protein | Q6P6N4 | Q6XBF4 | Q8BZL7 | Dlgap1 | Kiaa4162 | Q8C0G0 | Q8BZQ1 | PSD-95/SAP90-binding protein 1 | Gkap | Q52KF6 | Q5DTK5 | SAP90/PSD-95-associated protein 1
Q61767	5	0.010699955442027738			HSD3B4					10090	HSD3B4 | Q61767
P0CG50	5	0.008480760871426912		Ubc	RL40_MOUSE	Ubc			NP_062613	10090	RL40_MOUSE | RS27A_MOUSE | UBC | P0CG50 | UBC_MOUSE | UBB_MOUSE
P35438	5	0.0030738113410227856			GRIN1	Grin1			NP_001171128 | NP_001171127 | NP_032195	10090	P35438 | GRIN1
Q80TI5	5	0.007039067640826307			K-Cl cotransporter 2					10090	A2A5L0 | K-Cl cotransporter 2 | Q80TI5 | Kcc2 | Slc12a5 | Kiaa1176 | Q3UHQ2 | Q9Z0M7 | Electroneutral potassium-chloride cotransporter 2 | Neuronal K-Cl cotransporter | Q7TQC9
Q5DU45	5	0.010990723401389573			Centaurin-gamma-1					10090	Centaurin-gamma-1 | Q5DU45 | Centg1 | Phosphatidylinositol 3-kinase enhancer | Agap2 | Kiaa0167
O35157	4	0.006008128392898336			NCX1					10090	O35157 | Solute carrier family 8 member 1 | O35157_MOUSE | NCX1 | Slc8a1 | SLC8A1 | Na(+)/Ca(2+)-exchange protein 1
Q08460	4	0.004901576713822539			KCNMA1					10090	Q08460 | KCNMA1
O70263	4	0.0022860782282105117		Lnx1	LNX1				XP_006504317	10090	LNX1 | O70263 | LNX1_MOUSE
Q921E6	4	0.0030017686105746092		Eed	EED	Eed			NP_068676	10090	EED_MOUSE | EED | Q921E6
P04925	4	0.00513840350454403		Prnp	PRNP	Prnp			NP_035300 | NP_001265185	10090	PRNP | PRIO_MOUSE | P04925
Q80YT7	4	2.7962463832949616E-4			PDE4DIP					10090	Q80YT7 | PDE4DIP
Q80YA9	4	7.666194975573024E-4			CNKSR2					10090	CNKSR2 | Q80YA9
135137524	4	0.007329860378390052			135137524				XP_006532473	10090	
231643127	4	0.010067477805759121			231643127				NP_067398	10090	
235841583	4	0.009736006239626782			235841583				XP_006530013	10090	
Q99P53	4	0.003642337930379473			Gmt1					10090	Gmt1 | High-affinity neuronal glutamate transporter | Slc1a3 | Q99P53 | Eaat1 | Glial high affinity glutamate transporter | Sodium-dependent glutamate/aspartate transporter 1 | Solute carrier family 1 member 3
Q9R196	4	0.003883361578443761			BK channel					10090	BK channel | BKCA alpha | Q64703 | Q9R196 | Kcnma1 | Kcnma | Maxi K channel | Calcium-activated potassium channel, subfamily M subunit alpha-1 | Q8VHF1 | Slo-alpha | KCa1.1 | K(VCA)alpha | Slowpoke homolog | Slo1
Q9JMF2	4	0.0045097510381887735			Rd3					10090	Rd3 | Q3SYJ8 | Q9JMF2 | Q9CRS3 | Retinal degeneration protein 3
Q80TP2	4	4.971534703356104E-4			Kiaa0902					10090	Kiaa0902 | CNK homolog protein 2 | Cnksr2 | Q80TP2
Q8K240	4	7.727350981406097E-4			Phosphodiesterase 4D-interacting protein					10090	Phosphodiesterase 4D-interacting protein | Pde4dip | Q80U00 | Q8K240 | Kiaa0454 | Q05CH5 | C4IXU1
Q6WVG3	3	3.3547072861254436E-4	synaptic protein	Kctd12	KCD12_MOUSE		NP_808383.3	BTB/POZ domain-containing protein KCTD12		10090	KCD12_MOUSE | Q6WVG3 | KCTD12
Q9EPK7	3	8.279710536726966E-4			XPO7					10090	Q9EPK7 | XPO7
O88704	3	3.8354959500846384E-4			HCN1					10090	O88704 | HCN1
Q6AXH7	3	0.0043410462299390265			EZH2					10090	Q6AXH7 | Ezh2 | EZH2
Q8VDN2	3	0.003939411799935705		Atp1a1	ATP1A1	Atp1a1			NP_659149	10090	AT1A1_MOUSE | ATP1A1 | Q8VDN2
Q8BVF7	3	0.003129740927088343			APH1A	Aph1a			NP_666216 | NP_666246	10090	Q8BVF7 | APH1A
Q8C7N7	3	0.003129740927088343		Aph1b	APH1B	Aph1b			NP_808251	10090	APH1B | APH1B_MOUSE | Q8C7N7
Q8CG73	3	0.0028359171198198262		Rpgrip1l	FTM_MOUSE	Rpgrip1l			NP_775607	10090	FTM_MOUSE | Q8CG73 | RPGRIP1L
Q9Z2Y3	3	7.295375896643593E-4			HOMER1	Homer1			NP_671705 | NP_036112 | NP_687036 | NP_001271118	10090	HOMER1 | Q9Z2Y3
Q7TMY8	3	1.1588026042918769E-4			HUWE1					10090	Q7TMY8 | HUWE1
Q99KR7	3	0.008206097291537482		Ppif	Cyclophilin F				NP_598845	10090	Cyclophilin F | Cyclophilin D | Rotamase F | PPIF | Q99KR7 | Ppif | PPIF_MOUSE
Q9EP53	3	0.0028468042305390346			TSC1					10090	TSC1 | Q9EP53
Q91VA7	3	0.04845681477014898			IDH3B					10090	Q91VA7 | IDH3B | Idh3b
P54254	3	0.004427503080988539		Atxn1	ATX1_MOUSE				XP_006516944 | NP_033150	10090	ATX1_MOUSE | P54254
188672145	3	7.295375896643593E-4			188672145				XP_006517731	10090	
155620333	3	0.004019715717174139			155620333				XP_006513620	10090	
P08129	3	0.004160185139621601		Ppp1ca	PP1A_HUMAN				NP_114074	10090	PP1A_HUMAN | P08129 | PP1A_MOUSE | PP1A_RAT | PP1A_RABIT
ENSMUSG00000030209	3	2.973565004791682E-4			ENSMUSG00000030209	Grin2b			NP_032197	10090	
Q3T9E3	3	5.122319458887107E-4			Predominantly fetal expressed T1 domain					10090	Predominantly fetal expressed T1 domain | Pfetin | Q3T9E3 | Pfet1 | Kctd12
A2AI18	3	0.003182728495000575			N-methyl-D-aspartate receptor subunit NR1					10090	N-methyl-D-aspartate receptor subunit NR1 | Glutamate [NMDA] receptor subunit zeta-1 | Q8CFS4 | A2AI15 | Glurz1 | A2AI18 | Grin1

Q8R0E1	3	0.003450478729836599			Inositol polyphosphate-binding protein					10090	Inositol polyphosphate-binding protein | Q8R0E1 | Synaptotagmin II | Syt2
Q8CEF5	3	0.001581966459231819			Ranbp16					10090	Ranbp16 | Q8CEF5 | Q80TS9 | Q3TP94 | Q8CB42 | Xpo7 | Q8C9M7 | Kiaa0745 | Ran-binding protein 16 | Q8BSK5 | Q8CBL8
Q9D613	3	0.0015357708497792852			Hyperpolarization-activated cation channel 2					10090	Hyperpolarization-activated cation channel 2 | Q9D613 | Bcng1 | O54899 | Hac2 | Brain cyclic nucleotide-gated channel 1 | Hcn1
Q80VR7	3	0.00263378507075684			Kvlqt1					10090	Kvlqt1 | Q7TNZ1 | Kcna9 | O88702 | Q3U4H1 | IKs producing slow voltage-gated potassium channel subunit alpha KvLQT1 | Q80VR7 | Voltage-gated potassium channel subunit Kv7.1 | Kcnq1 | Q7TPL7 | KQT-like 1
Q9Z1Z4	3	8.933495010902704E-4			Flavoprotein subunit of complex II					10090	Q3V4B1 | Q3UKP7 | Flavoprotein subunit of complex II | Q3UH25 | Q9Z1Z4 | Sdha | Q0QF19 | Q921P5
Q64180	3	0.0020942613801371245			Acact					10090	Acact | Q64180 | Acyl-coenzyme A:cholesterol acyltransferase 1 | Soat1 | Cholesterol acyltransferase 1 | Q5XK32
B1ASZ9	3	0.0022256641142919817			Plasmalemmal porin					10090	Q5SVC6 | B1ASZ9 | Plasmalemmal porin | Outer mitochondrial membrane protein porin 1 | Voltage-dependent anion-selective channel protein 5 | Vdac1 | Vdac5
Q8CIE8	3	0.0021640795415307328			2-oxoglutarate dehydrogenase complex component E2					10090	2-oxoglutarate dehydrogenase complex component E2 | Dlst | Dihydrolipoamide succinyltransferase component of 2-oxoglutarate dehydrogenase complex | Q8CIE8 | Q4FK55 | Q3UEA0 | E2K
G3X958	3	0.0021640795415307328			Nphp8					10090	Nphp8 | RPGR-interacting protein 1-like protein | Nephrocystin-8 | Rpgrip1l | Ftm | G3X958
Q9R0P2	3	3.156896422135477E-4			Upstream regulatory element-binding protein 1					10090	Upstream regulatory element-binding protein 1 | Q9R0P2 | HECT-type E3 ubiquitin transferase HUWE1 | Q8BNJ7 | Q8VD14 | E3Histone | Q4G2Z1 | Q6NS61 | Ureb1 | Kiaa0312 | Q8CFH2 | HECT, UBA and WWE domain-containing protein 1 | Q5BMM7 | Huwe1 | A2AFQ1 | Q921M5
Q924U7	3	0.002417339597943618			Kiaa0243					10090	Kiaa0243 | A2AHW1 | Q80U55 | Q7TS92 | Tsc1 | Tuberous sclerosis 1 protein homolog | Q924U7 | Q3UHF2
Q9EPL2	2	1.328921321746954E-4			CLSTN1					10090	Q9EPL2 | CLSTN1
P27656	2	3.192926666728026E-4		Lipc	LIPC	Lipc			NP_032306	10090	LIPC | LIPC_MOUSE | P27656
Q8BL48	2	3.796178017485011E-4			UNK					10090	UNK | Q8BL48
Q91X05	2	5.159678997073054E-4		Nphs2	NPHS2				NP_569723	10090	NPHS2 | Q91X05 | PODO_MOUSE
O35526	2	5.63960654362749E-4		Stx1a	STX1A	Stx1a			NP_058081	10090	Neuron-specific antigen HPC-1 | O35526 | STX1A | STX1A_MOUSE | Stx1a
P62196	2	0.0		Psmc5	PRS8_MOUSE	Psmc5			NP_032976	10090	PRS8_MOUSE | PSMC5 | P62196
Q9QXA3	2	3.0438973158174274E-4			FAT1					10090	Q9QXA3 | FAT1 | Fat1 | mfat1 | Q9QXA3_MOUSE | Fath
Q9QWW1	2	0.0		Homer2	HOME2_MOUSE	Homer2			NP_001157558 | NP_001157559 | NP_036113	10090	HOME2_MOUSE | HOMER2 | Q9QWW1
P16054	2	0.0027166580245734074		Prkce	PRKCE				NP_035234	10090	P16054 | PRKCE | Prkce | Pkce | Pkcea | nPKC-epsilon | KPCE_MOUSE
P63213	2	0.0		Gng2	GBG2_MOUSE	Gng2			NP_001272840 | NP_034445 | NP_001033726 | XP_006518625 | NP_001272837 | NP_0	10090	GBG2_MOUSE | P63213 | GNG2
Q63ZW7	2	4.145941934111325E-5			INADL					10090	Q63ZW7 | INADL
Q99KI0	2	0.0010176897179051615		Aco2	ACO2	Aco2			NP_542364	10090	Q99KI0 | ACON_MOUSE | ACO2
Q7TMB8	2	2.5325868756466162E-5			CYFIP1					10090	CYFIP1 | Q7TMB8
O08785	2	4.606630859486616E-4		Clock	CLOCK	Clock			NP_001276755 | NP_031741	10090	O08785 | CLOCK_MOUSE | Clock | CLOCK
P08226	2	0.004658086928440904			Apoe					10090	Apoe | APOE | P08226
P0DI97	2	0.004658086928440904			NRX1B_MOUSE	Nrxn1			NP_796258 | NP_064648	10090	NRX1B_MOUSE | NRXN1 | Nrxn1 | P0DI97 | Neurexin I-beta | Q9CS84
P51881	2	0.0		Slc25a5	ADT2_MOUSE				NP_031477	10090	P51881 | Q545A2_MOUSE | ADT2_MOUSE | SLC25A5
Q07079	2	0.004658086928440904			Igfbp5					10090	Igfbp5 | IGFBP5 | Igfbp-5 | Q07079
Q3TS89	2	0.004658086928440904			Dipk2a					10090	Dipk2a | Q3TS89 | 1190002N15RIK | 1190002N15Rik
Q3UGZ9	2	0.004658086928440904			Nrxn2					10090	Nrxn2 | NRXN2 | Q3UGZ9
P22682	2	4.7658835860747955E-4			CBL					10090	P22682 | CBL
P10637	2	4.7658835860747955E-4			MAPT	Mapt			NP_034968 | NP_001033698 | NP_001272384	10090	P10637 | MAPT | TAU_MOUSE
P39689	2	0.0039010017618825907		Cdkn1a	CDN1A_MOUSE	Cdkn1a			NP_001104569 | NP_031695	10090	CDN1A_MOUSE | Q564P6_MOUSE | CDKN1A | Cip1 | Melanoma differentiation-associated protein | CDK-interacting protein 1 | P39689 | Cdkn1a | Waf1 | p21
Q64010	2	4.7658835860747955E-4			CRK	Crk			NP_001264148 | NP_598417 | NP_001264150	10090	Q64010 | CRK
Q9ERR1	2	4.7658835860747955E-4		Ndel1	NDEL1				NP_076157	10090	NDEL1_MOUSE | Q9ERR1 | NDEL1
O54957	2	0.0039010017618825907			36 kDa phospho-tyrosine adapter protein					10090	36 kDa phospho-tyrosine adapter protein | p36-38 | LAT | Lat | O54957
Q61337	2	0.0039010017618825907		Bad	Bcl2 antagonist of cell death	Bad			NP_001272382 | NP_031548	10090	Bcl2 antagonist of cell death | Bcl-2-binding component 6 | Q61337 | Bad | BAD | BAD_MOUSE | Bbc6 | Bcl-xL/Bcl-2-associated death promoter
Q5S006	2	4.7658835860747955E-4		Lrrk2	LRRK2	Lrrk2			NP_080006	10090	LRRK2 | LRRK2_MOUSE | Q5S006
Q60787	2	4.7658835860747955E-4			LCP2					10090	Q60787 | LCP2
O35626	2	9.882644038354195E-4		Rasd1	Rasd1	Rasd1			NP_033052	10090	Rasd1 | RASD1_MOUSE | RASD1 | Dexras1 | O35626
P35569	2	0.002105425340453376			IRS1					10090	Irs-1 | IRS1 | Irs1 | P35569
167151324	2	4.484998245303427E-4			167151324				XP_011246613	10090	
238940625	2	4.484998245303427E-4			238940625				XP_006532209	10090	
P56391	2	6.478385048457153E-4		Cox6b1	CX6B1_MOUSE				NP_079904	10090	CX6B1_MOUSE | P56391
5337594	2	9.997560490095526E-4		Kcnma1	5337594				XP_006518668	10090	
P14231	2	0.0		Atp1b2	AT1B2_MOUSE				NP_038201	10090	P14231 | AT1B2_MOUSE
Q8VCE0	2	0.0		Atp1a3	Q8VCE0				XP_011248821	10090	Q8VCE0_MOUSE | Q8VCE0
P32300	2	0.0		Oprd1	OPRD_MOUSE				NP_038650	10090	OPRD_MOUSE | P32300
G3X9V4	2	7.295375896643593E-4		Grin2b	G3X9V4				NP_032197	10090	G3X9V4 | G3X9V4_MOUSE
D3YZU1	2	0.004065245397989031		Shank1	Shank1				NP_001029287	10090	Shank1 | SHAN1_MOUSE | D3YZU1
250930020	2	0.0014170413083502763			250930020				XP_006501375	10090	
167105844	2	0.008761166098010935			167105844				XP_006540281	10090	
P35486	2	0.0022890109355996695		Pdha1	Pdha1				NP_032836	10090	Pdha1 | Pdha-1 | ODPA_MOUSE | P35486 | PDHE1-A type I
264645899	2	0.00284489919492349			264645899				XP_006527639	10090	
P11798-2	2	0.0013050455995104314		Camk2a	Camk2a				NP_033922	10090	P11798-2 | Camk2a
P42859	2	0.0028174114063899414			HD					10090	P42859 | HD_MOUSE | Htt | Huntington disease protein homolog | Hd | Hdh
1542574	2	4.959676994833306E-4		Htt	1542574				NP_034544	10090	
210062929	2	0.0			210062929				XP_006509742	10090	
B2RRX1	2	9.95993278025317E-4		Actb	ACTB_CHICK				NP_031419	10090	ACTB_CHICK | ACTB_HUMAN | ACTB_HORSE | ACTB_MOUSE | B2RRX1 | ACTB_BOVIN
Q0PHV7	2	4.606630859486616E-4		Dact3	Dapper antagonist of catenin 3	Dact3			NP_001075124	10090	Dapper antagonist of catenin 3 | DACT3_MOUSE | Q0PHV7 | Dact3
119302390	2	0.0017607992495595837		Dlgap1	119302390				XP_006524238	10090	
Q61019	2	0.0017607992495595837		Gnai3	GNAI3_MOUSE				NP_034436	10090	Q61019 | GNAI3_MOUSE
E9Q238	2	0.0039010017618825907		Kank1	Kank1					10090	Kank1 | E9Q238 | E9Q238_MOUSE
ENSMUSG00000039943	2	0.0			ENSMUSG00000039943	Plcb4			NP_038857	10090	
ENSMUSG00000024589	2	4.7658835860747955E-4			ENSMUSG00000024589	Nedd4l			NP_001107858 | NP_114087	10090	
Q921I6	2	4.7658835860747955E-4			SH3B4_MOUSE	Sh3bp4			NP_598577	10090	SH3B4_MOUSE | Q921I6
Q3V3Y4	2	7.526196531982062E-4			Hdac4					10090	Q3TRZ9 | Hdac4 | Q3V3Y4 | Q3U2J3
O70298	2	0.0027729253223540045			Kiaa1684					10090	p130Cas-associated protein | O70298 | Kiaa1684 | P140 | SNAP-25-interacting protein | p140Cap | Srcin1
Q62125	2	0.0022720365887003205			HGF/SF receptor					10090	HGF/SF receptor | Proto-oncogene c-Met | Tyrosine-protein kinase Met | Met | Q62125 | Scatter factor receptor
Q9DCB2	2	0.0015004499859020755			Glutamate [NMDA] receptor subunit epsilon-2					10090	Glutamate [NMDA] receptor subunit epsilon-2 | Q9DCB2 | N-methyl D-aspartate receptor subtype 2B | Grin2b
Q9JL73	2	5.099948035309669E-4			Hdac5					10090	Hdac5 | Q9JL73 | Histone deacetylase mHDA1
Q9EPT6	2	1.6487007789791486E-4			Nudel					10090	Nudel | Q9EPT6 | Q9D0Q4 | Ndel1 | Protein mNudE-like
Q9EPL2-PRO_0000323600	2	6.545044354193273E-4			Cs1					10090	Cs1 | Clstn1 | Cstn1 | Q9EPL2-PRO_0000323600 | Kiaa0911 | Alcadein-alpha
Q9CZY7	2	0.0012328960824722335			Tmem173					10090	Q3TAV5 | Tmem173 | Q3TYP5 | Transmembrane protein 173 | Q9CZY7 | Sting1 | Endoplasmic reticulum interferon stimulator | Sting | Q3TZY8 | Mita | Mediator of IRF3 activation | Eris | Q8C227 | Q8C5Q3 | Q8K393 | Mpys | Q3UJW3 | A7YGY9
Q921K5	2	3.921817397717415E-4			Cox15					10090	Q3URB2 | Q921K5 | Q8VDN0 | Cox15
Q9D7M9	2	3.921817397717415E-4			Para-hydroxybenzoate--polyprenyltransferase					10090	Para-hydroxybenzoate--polyprenyltransferase | E9QPH1 | Q9D7M9 | Coq2
Q5EBQ1	2	1.4038421372749454E-4			Protein kinase C inhibitor protein 1					10090	Protein kinase C inhibitor protein 1 | Q5EBQ1 | P97286 | Q3TSF1 | P70197 | Ywhaz | P35215 | SEZ-2
O88558	2	8.577280264694067E-5			Sra1					10090	Q3U7Q7 | Q99LY1 | Q7TSZ5 | Sra1 | Specifically Rac1-associated protein 1 | Q8CE85 | Q80VN6 | Q5DU50 | O88558 | Cyfip1 | Shyc | Kiaa0068
let-7d-5p_mouse_rna	2	1.6098186981753162E-4			let-7d-5p_mouse_rna					10090	
Q9Z1G2	2	8.475431836182029E-4			Ppp1a					10090	P22802 | P08129 | Q3U7G7 | Ppp1a | P20653 | Ppp1ca | Q9Z1G2
Q91Z09	2	0.006782382137737366			Atp1a1					10090	Atp1a1 | Q91Z09 | Sodium pump subunit alpha-1
P08228	2	1.6487007789791486E-4			Sod1					10090	P08228 | Sod1
Q8K062	2	1.6487007789791486E-4			Lrrk2					10090	Q8BWG7 | Q8CI84 | Q8BZJ6 | Lrrk2 | Q8K062 | E9QNJ2
Q64010-1	2	1.6487007789791486E-4			Proto-oncogene c-Crk					10090	Proto-oncogene c-Crk | Q64010-1 | Crko | p38 | Crk
Q922M0	2	1.6487007789791486E-4			SH2 domain-containing leukocyte protein of 76 kDa					10090	Q922M0 | SH2 domain-containing leukocyte protein of 76 kDa | Lcp2 | SLP-76 tyrosine phosphoprotein
Q8CEA1	2	1.6487007789791486E-4			Signal transduction protein CBL					10090	Q8CEA1 | Q3U527 | Signal transduction protein CBL | Cbl | RING-type E3 ubiquitin transferase CBL | Proto-oncogene c-Cbl | Casitas B-lineage lymphoma proto-oncogene
Q99LD3	2	1.6487007789791486E-4			Dihydrolipoamide dehydrogenase					10090	Dihydrolipoamide dehydrogenase | Q3UWP7 | Q3TG55 | Q99LD3 | Dld | Q3U5W5
Q9D1G1	1	0.0			RAB1B					10090	Q9D1G1 | RAB1B
Q8C437	1	0.0			PEX5L					10090	Q8C437 | PEX5L
P70414	1	0.0			SLC8A1					10090	SLC8A1 | P70414
P28661	1	0.0			SEPT4	Sept4			NP_001271321 | NP_001271323 | NP_035259 | NP_001271327	10090	P28661 | SEPT4 | SEPT4_MOUSE
P56371	1	0.0			RAB4A					10090	RAB4A | P56371
Q8K245	1	0.0		Uvrag	UVRAG				NP_848750	10090	UVRAG | UVRAG_MOUSE | Q8K245
P50516	1	0.0			ATP6V1A					10090	ATP6V1A | P50516
P52927	1	0.0			HMGA2					10090	P52927 | HMGA2

<table>
<tr><td></td><td></td><td></td><td></td><td></td><td></td><td></td><td></td><td></td><td></td><td></td><td></td></tr>
<tr><td>Q99JY9</td><td>1</td><td>0.0</td><td></td><td></td><td>ACTR3</td><td>Actr3</td><td></td><td></td><td>NP_076224|NP_001192315|NP_001192314</td><td>10090</td><td>ACTR3|Q99JY9</td></tr>
<tr><td>P25801</td><td>1</td><td>0.0</td><td></td><td></td><td>LMO2</td><td></td><td></td><td></td><td></td><td>10090</td><td>LMO2|P25801</td></tr>
<tr><td>P60879</td><td>1</td><td>0.0</td><td></td><td>Snap25</td><td>SNP25_MOUSE</td><td>Snap25</td><td></td><td></td><td>NP_001277985|NP_035558</td><td>10090</td><td>SNAP25|SNP25_MOUSE|P60879</td></tr>
<tr><td>Q8BP00</td><td>1</td><td>0.0</td><td></td><td></td><td>IQCB1</td><td>Iqcb1</td><td></td><td></td><td>NP_796102</td><td>10090</td><td>IQCB1|Q8BP00</td></tr>
<tr><td>P97449</td><td>1</td><td>0.0</td><td></td><td></td><td>ANPEP</td><td></td><td></td><td></td><td></td><td>10090</td><td>ANPEP|P97449</td></tr>
<tr><td>Q6PDS3</td><td>1</td><td>0.0</td><td></td><td></td><td>SARM1</td><td></td><td></td><td></td><td></td><td>10090</td><td>SARM1|Q6PDS3</td></tr>
<tr><td>P63011</td><td>1</td><td>0.0</td><td></td><td></td><td>RAB3A</td><td></td><td></td><td></td><td></td><td>10090</td><td>RAB3A|P63011</td></tr>
<tr><td>Q9Z1L4</td><td>1</td><td>0.0</td><td></td><td>Rs1</td><td>RS1</td><td></td><td></td><td></td><td></td><td>10090</td><td>RS1|Rs1|Q9Z1L4|X-linked juvenile retinoschisis protein homolog|Xlrs1|XLRS1_MOUSE|Rs1h</td></tr>
<tr><td>Q9WV18</td><td>1</td><td>0.0</td><td></td><td>Gabbr1</td><td>GABBR1</td><td>Gabbr1</td><td></td><td></td><td>NP_062312</td><td>10090</td><td>GABBR1|GABR1_MOUSE|Q9WV18</td></tr>
<tr><td>O55222</td><td>1</td><td>0.0</td><td></td><td></td><td>ILK</td><td></td><td></td><td></td><td></td><td>10090</td><td>O55222|ILK</td></tr>
<tr><td>Q9QXD6</td><td>1</td><td>0.0</td><td></td><td></td><td>FBP1</td><td>Fbp1</td><td></td><td></td><td>NP_062268</td><td>10090</td><td>Q9QXD6|FBP1</td></tr>
<tr><td>O70589</td><td>1</td><td>0.0</td><td></td><td></td><td>CASK</td><td></td><td></td><td></td><td></td><td>10090</td><td>O70589|CASK</td></tr>
<tr><td>P23819</td><td>1</td><td>0.0</td><td></td><td></td><td>GRIA2</td><td></td><td></td><td></td><td></td><td>10090</td><td>GRIA2|P23819</td></tr>
<tr><td>P06151</td><td>1</td><td>0.0</td><td></td><td></td><td>LDHA</td><td>Ldha</td><td></td><td></td><td>NP_034829|NP_001129541</td><td>10090</td><td>LDHA|P06151</td></tr>
<tr><td>Q8VDU0</td><td>1</td><td>0.0</td><td></td><td></td><td>GPSM2</td><td>Gpsm2</td><td></td><td></td><td>NP_083798</td><td>10090</td><td>GPSM2|Q8VDU0</td></tr>
<tr><td>P12660</td><td>1</td><td>0.0</td><td></td><td></td><td>PCP2</td><td>Pcp2</td><td></td><td></td><td>NP_032816|NP_001123276|NP_001123275</td><td>10090</td><td>PCP2|P12660|PCP2_MOUSE</td></tr>
<tr><td>P12023</td><td>1</td><td>0.0</td><td></td><td>App</td><td>APP</td><td>App</td><td></td><td></td><td>NP_031497|NP_001185753|NP_001185752|NP_001185755|NP_001185754</td><td>10090</td><td>APP|A4_MOUSE|P12023</td></tr>
<tr><td>P97428</td><td>1</td><td>0.0</td><td></td><td>Rgs16</td><td>RGS16</td><td>Rgs16</td><td></td><td></td><td>NP_035397</td><td>10090</td><td>RGS16_MOUSE|RGS16|P97428</td></tr>
<tr><td>Q9JKB3</td><td>1</td><td>0.0</td><td></td><td>Ybx3</td><td>YBX3</td><td>Ybx3</td><td></td><td></td><td>NP_620817|NP_035863</td><td>10090</td><td>Q9JKB3|YBOX3_MOUSE|YBX3</td></tr>
<tr><td>P38647</td><td>1</td><td>0.0</td><td></td><td>Hspa9</td><td>GRP75_MOUSE</td><td></td><td></td><td></td><td>NP_034611</td><td>10090</td><td>HSPA9|P38647|GRP75_MOUSE</td></tr>
<tr><td>Q9D1Q6</td><td>1</td><td>0.0</td><td></td><td>Erp44</td><td>ERP44</td><td>Erp44</td><td></td><td></td><td>NP_083848</td><td>10090</td><td>Q9D1Q6|ERP44|ERP44_MOUSE</td></tr>
<tr><td>P60670</td><td>1</td><td>0.0</td><td></td><td></td><td>NPLOC4</td><td>Nploc4</td><td></td><td></td><td>NP_001181952|NP_955763</td><td>10090</td><td>NPLOC4|P60670</td></tr>
<tr><td>P70362</td><td>1</td><td>0.0</td><td></td><td>Ufd1</td><td>UFD1L</td><td>Ufd1l</td><td></td><td></td><td>NP_035802</td><td>10090</td><td>UFD1L|P70362|UFD1_MOUSE</td></tr>
<tr><td>Q8BFZ9</td><td>1</td><td>0.0</td><td></td><td>Erlin2</td><td>ERLN2_MOUSE</td><td>Erlin2</td><td></td><td></td><td>NP_705820</td><td>10090</td><td>ERLN2_MOUSE|Q8BFZ9|ERLIN2</td></tr>
<tr><td>Q8CBG9</td><td>1</td><td>0.0</td><td></td><td></td><td>RNF170</td><td>Rnf170</td><td></td><td></td><td>NP_084241</td><td>10090</td><td>RN170_MOUSE|Q8CBG9|RNF170</td></tr>
<tr><td>Q9ES00</td><td>1</td><td>0.0</td><td></td><td>Ube4b</td><td>UBE4B</td><td>Ube4b</td><td></td><td></td><td>NP_071305</td><td>10090</td><td>Q9ES00|UBE4B|UBE4B_MOUSE</td></tr>
<tr><td>Q01853</td><td>1</td><td>0.0</td><td></td><td>Vcp</td><td>VCP</td><td>Vcp</td><td></td><td></td><td>NP_033529</td><td>10090</td><td>Q01853|VCP|TERA_MOUSE</td></tr>
<tr><td>Q60932</td><td>1</td><td>0.0</td><td></td><td>Vdac1</td><td>VDAC1</td><td></td><td></td><td></td><td></td><td>10090</td><td>VDAC1_MOUSE|Q60932|VDAC1</td></tr>
<tr><td>Q9QUQ5</td><td>1</td><td>0.0</td><td></td><td></td><td>TRPC4</td><td>Trpc4</td><td></td><td></td><td>NP_001240612|NP_001240611|NP_058680</td><td>10090</td><td>Q9QUQ5|TRPC4|TRPC4_MOUSE</td></tr>
<tr><td>Q8BPN8</td><td>1</td><td>0.0</td><td></td><td></td><td>DMXL2</td><td>Dmxl2</td><td></td><td></td><td>NP_766359</td><td>10090</td><td>Q8BPN8|DMXL2</td></tr>
<tr><td>Q9JKS4</td><td>1</td><td>0.0</td><td></td><td>Ldb3</td><td>LDB3</td><td>Ldb3</td><td></td><td></td><td>NP_001034165|NP_036048|NP_001034163|NP_001034162|NP_001034161|NP_0</td><td>10090</td><td>Q9JKS4|LDB3|LDB3_MOUSE</td></tr>
<tr><td>Q8BH60</td><td>1</td><td>0.0</td><td></td><td>Gopc</td><td>GOPC</td><td>Gopc</td><td></td><td></td><td>NP_444417|NP_001186201</td><td>10090</td><td>GOPC|Q8BH60|GOPC_MOUSE</td></tr>
<tr><td>Q9R171</td><td>1</td><td>0.0</td><td></td><td>Cbln1</td><td>CBLN1</td><td>Cbln1</td><td></td><td></td><td>NP_062600</td><td>10090</td><td>Q9R171|CBLN1_MOUSE|CBLN1</td></tr>
<tr><td>Q9WU22</td><td>1</td><td>0.0</td><td></td><td>Ptpn4</td><td>PTN4_MOUSE</td><td>Ptpn4</td><td></td><td></td><td>NP_064317</td><td>10090</td><td>Q9WU22|PTN4_MOUSE|PTPN4</td></tr>
<tr><td>Q0QWG9</td><td>1</td><td>0.0</td><td></td><td></td><td>GRID2IP</td><td>Grid2ip</td><td></td><td></td><td>NP_001152793|NP_579933</td><td>10090</td><td>Q0QWG9|GRID2IP</td></tr>
<tr><td>B0V2N1</td><td>1</td><td>0.0</td><td></td><td></td><td>PTPRS</td><td></td><td></td><td></td><td></td><td>10090</td><td>B0V2N1|PTPRS</td></tr>
<tr><td>O88531</td><td>1</td><td>0.0</td><td></td><td></td><td>PPT1</td><td></td><td></td><td></td><td></td><td>10090</td><td>PPT1|O88531</td></tr>
<tr><td>O88597</td><td>1</td><td>0.0</td><td></td><td>Becn1</td><td>BECN1</td><td>Becn1</td><td></td><td></td><td>NP_062530</td><td>10090</td><td>BECN1|O88597|BECN1_MOUSE</td></tr>
<tr><td>P10126</td><td>1</td><td>0.0</td><td></td><td></td><td>EEF1A1</td><td></td><td></td><td></td><td></td><td>10090</td><td>EEF1A1|P10126</td></tr>
<tr><td>P11214</td><td>1</td><td>0.0</td><td></td><td></td><td>PLAT</td><td></td><td></td><td></td><td></td><td>10090</td><td>P11214|PLAT</td></tr>
<tr><td>P14106</td><td>1</td><td>0.0</td><td></td><td></td><td>P14106</td><td></td><td></td><td></td><td></td><td>10090</td><td>C1QB|P14106</td></tr>
<tr><td>P16858</td><td>1</td><td>0.0</td><td></td><td></td><td>GAPDH</td><td></td><td></td><td></td><td></td><td>10090</td><td>P16858|GAPDH</td></tr>
<tr><td>P20152</td><td>1</td><td>0.0</td><td></td><td></td><td>VIM</td><td></td><td></td><td></td><td></td><td>10090</td><td>P20152|VIM</td></tr>
<tr><td>P49312</td><td>1</td><td>0.0</td><td></td><td></td><td>HNRNPA1</td><td></td><td></td><td></td><td></td><td>10090</td><td>P49312|HNRNPA1</td></tr>
<tr><td>P54320</td><td>1</td><td>0.0</td><td></td><td></td><td>ELN</td><td></td><td></td><td></td><td></td><td>10090</td><td>P54320|ELN</td></tr>
<tr><td>P62908</td><td>1</td><td>0.0</td><td></td><td></td><td>RPS3</td><td></td><td></td><td></td><td></td><td>10090</td><td>P62908|RPS3</td></tr>
<tr><td>Q5F226</td><td>1</td><td>0.0</td><td></td><td></td><td>FAT2</td><td></td><td></td><td></td><td></td><td>10090</td><td>Q5F226|FAT2</td></tr>
<tr><td>Q60963</td><td>1</td><td>0.0</td><td></td><td></td><td>PLA2G7</td><td></td><td></td><td></td><td></td><td>10090</td><td>Q60963|PLA2G7</td></tr>
<tr><td>Q64332</td><td>1</td><td>0.0</td><td></td><td></td><td>SYN2</td><td></td><td></td><td></td><td></td><td>10090</td><td>Q64332|SYN2</td></tr>
<tr><td>Q80XN0</td><td>1</td><td>0.0</td><td></td><td></td><td>BDH1</td><td></td><td></td><td></td><td></td><td>10090</td><td>BDH1|Q80XN0</td></tr>
<tr><td>Q91V61</td><td>1</td><td>0.0</td><td></td><td></td><td>SFXN3</td><td></td><td></td><td></td><td></td><td>10090</td><td>SFXN3|Q91V61</td></tr>
<tr><td>Q925N0</td><td>1</td><td>0.0</td><td></td><td></td><td>SFXN5</td><td></td><td></td><td></td><td></td><td>10090</td><td>Q925N0|SFXN5</td></tr>
<tr><td>Q99JR1</td><td>1</td><td>0.0</td><td></td><td></td><td>SFXN1</td><td></td><td></td><td></td><td></td><td>10090</td><td>SFXN1|Q99JR1</td></tr>
<tr><td>Q9CS84</td><td>1</td><td>0.0</td><td></td><td></td><td>NRXN1</td><td></td><td></td><td></td><td></td><td>10090</td><td>NRXN1|Q9CS84</td></tr>
<tr><td>Q9CWF2</td><td>1</td><td>0.0</td><td></td><td></td><td>TUBB2B</td><td></td><td></td><td></td><td></td><td>10090</td><td>TUBB2B|Q9CWF2</td></tr>
<tr><td>Q9D6E4</td><td>1</td><td>0.0</td><td></td><td></td><td>TTC9B</td><td></td><td></td><td></td><td></td><td>10090</td><td>Q9D6E4|TTC9B</td></tr>
<tr><td>Q9D6M3</td><td>1</td><td>0.0</td><td></td><td></td><td>SLC25A22</td><td></td><td></td><td></td><td></td><td>10090</td><td>Q9D6M3|SLC25A22</td></tr>
<tr><td>Q9DB41</td><td>1</td><td>0.0</td><td></td><td></td><td>SLC25A18</td><td></td><td></td><td></td><td></td><td>10090</td><td>SLC25A18|Q9DB41</td></tr>
<tr><td>Q9QWI6</td><td>1</td><td>0.0</td><td></td><td></td><td>SRCIN1</td><td></td><td></td><td></td><td></td><td>10090</td><td>Q9QWI6|SRCIN1</td></tr>
<tr><td>Q9WUR2</td><td>1</td><td>0.0</td><td></td><td></td><td>ECI2</td><td></td><td></td><td></td><td></td><td>10090</td><td>Q9WUR2|ECI2</td></tr>
<tr><td>P15806</td><td>1</td><td>0.0</td><td></td><td></td><td>TFE2_MOUSE</td><td>Tcf3</td><td></td><td></td><td>NP_001157624|NP_001157623|NP_001157625|NP_001157619|NP_035678|NP_0</td><td>10090</td><td>TFE2_MOUSE|P15806|TCF3</td></tr>
<tr><td>P43404</td><td>1</td><td>0.0</td><td></td><td></td><td>ZAP70</td><td></td><td></td><td></td><td></td><td>10090</td><td>P43404|ZAP70</td></tr>
<tr><td>P23804</td><td>1</td><td>0.0</td><td></td><td>Mdm2</td><td>MDM2</td><td>Mdm2</td><td></td><td></td><td>NP_001275515|NP_034916</td><td>10090</td><td>P23804|MDM2_MOUSE|MDM2</td></tr>
<tr><td>Q9D1F4</td><td>1</td><td>0.0</td><td></td><td>Akt1s1</td><td>AKT1S1</td><td>Akt1s1</td><td></td><td></td><td>NP_080546|NP_001277623|NP_001240849</td><td>10090</td><td>AKTS1_MOUSE|AKT1S1|Q9D1F4</td></tr>
<tr><td>Q8K4Q0</td><td>1</td><td>0.0</td><td></td><td></td><td>RPTOR</td><td></td><td></td><td></td><td></td><td>10090</td><td>RPTOR|RPTOR_MOUSE|Q8K4Q0</td></tr>
<tr><td>Q8CH72</td><td>1</td><td>0.0</td><td></td><td>Trim32</td><td>TRI32_MOUSE</td><td>Trim32</td><td></td><td></td><td>XP_006538299|NP_444314|NP_001155254</td><td>10090</td><td>TRI32_MOUSE|Q8CH72|TRIM32</td></tr>
<tr><td>P11688</td><td>1</td><td>0.0</td><td></td><td>Itga5</td><td>ITA5_MOUSE</td><td></td><td></td><td></td><td>NP_034707</td><td>10090</td><td>P11688|ITGA5|ITA5_MOUSE</td></tr>
<tr><td>P23242</td><td>1</td><td>0.0</td><td></td><td>Gja1</td><td>CXA1_MOUSE</td><td></td><td></td><td></td><td>NP_034418</td><td>10090</td><td>CXA1_MOUSE|GJA1|P23242</td></tr>
<tr><td>Q3U1F9</td><td>1</td><td>0.0</td><td></td><td></td><td>PAG1</td><td></td><td></td><td></td><td></td><td>10090</td><td>PAG1|Q3U1F9</td></tr>
<tr><td>Q80XE1</td><td>1</td><td>0.0</td><td></td><td></td><td>RIC8B</td><td></td><td></td><td></td><td></td><td>10090</td><td>RIC8B|Q80XE1</td></tr>
<tr><td>P35991</td><td>1</td><td>0.0</td><td></td><td>Btk</td><td>BTK</td><td>Btk</td><td></td><td></td><td>NP_038510</td><td>10090</td><td>P35991|BTK_MOUSE|BTK</td></tr>
<tr><td>Q99MK8</td><td>1</td><td>0.0</td><td></td><td>Grk2</td><td>ADRBK1</td><td>Adrbk1</td><td></td><td></td><td>NP_001277747|NP_570933</td><td>10090</td><td>ADRBK1|ARBK1_MOUSE|Q99MK8</td></tr>
<tr><td>P20263</td><td>1</td><td>0.0</td><td></td><td>Pou5f1</td><td>POU5F1</td><td>Pou5f1</td><td></td><td></td><td>NP_038661|NP_001239381</td><td>10090</td><td>PO5F1_MOUSE|P20263|POU5F1</td></tr>
<tr><td>O88485</td><td>1</td><td>0.0</td><td></td><td></td><td>DYNC1I1</td><td>Dync1i1</td><td></td><td></td><td>NP_034193|NP_001177955|NP_001177956|NP_001177954|NP_001177952</td><td>10090</td><td>O88485|DYNC1I1</td></tr>
<tr><td>P68040</td><td>1</td><td>0.0</td><td></td><td></td><td>GNB2L1</td><td>Gnb2l1</td><td></td><td></td><td>NP_032169</td><td>10090</td><td>P68040|GNB2L1</td></tr>
<tr><td>P48678</td><td>1</td><td>0.0</td><td></td><td></td><td>LMNA</td><td>Lmna</td><td></td><td></td><td>NP_062263|NP_001104572|NP_001002011</td><td>10090</td><td>LMNA|P48678</td></tr>
<tr><td>Q05512</td><td>1</td><td>0.0</td><td></td><td></td><td>MARK2</td><td>Mark2</td><td></td><td></td><td>NP_031954|NP_001073859|NP_001073858|NP_001073857</td><td>10090</td><td>Q05512|MARK2</td></tr>
<tr><td>Q91XL2</td><td>1</td><td>0.0</td><td></td><td></td><td>LNX2</td><td></td><td></td><td></td><td></td><td>10090</td><td>Q91XL2|LNX2</td></tr>
<tr><td>Q03141</td><td>1</td><td>0.0</td><td></td><td></td><td>MARK3</td><td>Mark3</td><td></td><td></td><td>NP_073712|NP_067491</td><td>10090</td><td>MARK3|Q03141</td></tr>
<tr><td>Q9CPU7</td><td>1</td><td>0.0</td><td></td><td></td><td>FBXO32</td><td>Fbxo32</td><td></td><td></td><td>NP_080622</td><td>10090</td><td>Q9CPU7|FBXO32</td></tr>
<tr><td>Q923S6</td><td>1</td><td>0.0</td><td></td><td></td><td>NEURL1</td><td>Neurl1a</td><td></td><td></td><td>NP_001156952|NP_067335</td><td>10090</td><td>NEURL1|Q923S6</td></tr>
<tr><td>P11416</td><td>1</td><td>0.0</td><td></td><td></td><td>RARA</td><td>Rara</td><td></td><td></td><td>NP_001169999|NP_001170774|NP_001170773|NP_033050</td><td>10090</td><td>P11416|RARA|RARA_MOUSE</td></tr>
<tr><td>O35618</td><td>1</td><td>0.0</td><td></td><td></td><td>MDM4</td><td>Mdm4</td><td></td><td></td><td>NP_032601</td><td>10090</td><td>O35618|MDM4</td></tr>
<tr><td>Q7TPH6</td><td>1</td><td>0.0</td><td></td><td></td><td>MYCBP2</td><td></td><td></td><td></td><td></td><td>10090</td><td>Q7TPH6|MYCBP2</td></tr>
<tr><td>P11798</td><td>1</td><td>0.0</td><td></td><td>Camk2a</td><td>KCC2A_MOUSE</td><td>Camk2a</td><td></td><td></td><td>NP_033922|NP_803126|NP_001273738</td><td>10090</td><td>KCC2A_MOUSE|CAMK2A|P11798</td></tr>
<tr><td>Q61584</td><td>1</td><td>0.0</td><td></td><td></td><td>FXR1</td><td></td><td></td><td></td><td></td><td>10090</td><td>FXR1|Q61584</td></tr>
<tr><td>Q5SQX6</td><td>1</td><td>0.0</td><td></td><td></td><td>CYFIP2</td><td></td><td></td><td></td><td></td><td>10090</td><td>CYFIP2|Q5SQX6</td></tr>
<tr><td>P42337</td><td>1</td><td>0.0</td><td></td><td></td><td>PIK3CA</td><td>Pik3ca</td><td></td><td></td><td>NP_032865</td><td>10090</td><td>PIK3CA|PK3CA_MOUSE|P42337</td></tr>
<tr><td>Q99N57</td><td>1</td><td>0.0</td><td></td><td></td><td>RAF1</td><td>Raf1</td><td></td><td></td><td>NP_084056</td><td>10090</td><td>RAF1_MOUSE|Q99N57|RAF1</td></tr>
<tr><td>Q61037</td><td>1</td><td>0.0</td><td></td><td></td><td>TSC2</td><td></td><td></td><td></td><td></td><td>10090</td><td>Q61037|TSC2</td></tr>
<tr><td>Q9WV31</td><td>1</td><td>0.0</td><td></td><td>Arc</td><td>ARC</td><td>Arc</td><td></td><td></td><td>NP_001263613|NP_061260</td><td>10090</td><td>Q9WV31|ARC|ARC_MOUSE</td></tr>
<tr><td>Q9JJ28</td><td>1</td><td>0.0</td><td></td><td>Flii</td><td>FLII</td><td>Flii</td><td></td><td></td><td>NP_071292</td><td>10090</td><td>Q9JJ28|FLII|FLII_MOUSE</td></tr>
<tr><td>A2CG49</td><td>1</td><td>0.0</td><td></td><td></td><td>KALRN</td><td></td><td></td><td></td><td></td><td>10090</td><td>A2CG49|KALRN</td></tr>
<tr><td>URS00002620A7_10090</td><td>1</td><td>0.0</td><td></td><td></td><td>URS00002620A7_10090</td><td></td><td></td><td></td><td></td><td>10090</td><td></td></tr>
<tr><td>2675810</td><td>1</td><td>0.0</td><td></td><td>Septin4</td><td>2675810</td><td></td><td></td><td></td><td>XP_006532556</td><td>10090</td><td></td></tr>
<tr><td>241045695</td><td>1</td><td>0.0</td><td></td><td></td><td>241045695</td><td></td><td></td><td></td><td>XP_006515387</td><td>10090</td><td></td></tr>
<tr><td>186118204</td><td>1</td><td>0.0</td><td></td><td></td><td>186118204</td><td></td><td></td><td></td><td>XP_017168713</td><td>10090</td><td></td></tr>
<tr><td>P12960</td><td>1</td><td>0.0</td><td></td><td>Cntn1</td><td>CNTN1_MOUSE</td><td></td><td></td><td></td><td>NP_031753|XP_017171911</td><td>10090</td><td>CNTN1_MOUSE|P12960</td></tr>
<tr><td>P31648</td><td>1</td><td>0.0</td><td></td><td>Slc6a1</td><td>SC6A1_MOUSE</td><td></td><td></td><td></td><td>NP_848818</td><td>10090</td><td>P31648|SC6A1_MOUSE</td></tr>
<tr><td>P99028</td><td>1</td><td>0.0</td><td></td><td>Uqcrh</td><td>QCR6_MOUSE</td><td></td><td></td><td></td><td>NP_079917</td><td>10090</td><td>P99028|QCR6_MOUSE</td></tr>
<tr><td>129119046</td><td>1</td><td>0.0</td><td></td><td></td><td>129119046</td><td></td><td></td><td></td><td>XP_006536971</td><td>10090</td><td></td></tr>
<tr><td>P60202</td><td>1</td><td>0.0</td><td></td><td>Plp1</td><td>MYPR_MOUSE</td><td></td><td></td><td></td><td>XP_017173919</td><td>10090</td><td>P60202|MYPR_MOUSE</td></tr>
<tr><td>Q4VA93</td><td>1</td><td>0.0</td><td></td><td>Prkca</td><td>Q4VA93</td><td></td><td></td><td></td><td>NP_035231</td><td>10090</td><td>Q4VA93|Q4VA93_MOUSE</td></tr>
<tr><td>Q61555</td><td>1</td><td>0.0</td><td></td><td>Fbn2</td><td>FBN2_MOUSE</td><td></td><td></td><td></td><td>NP_034311</td><td>10090</td><td>Q61555|FBN2_MOUSE</td></tr>
</table>

Q62277	1	0.0		Syp	SYPH_MOUSE				NP_033331	10090	SYPH_MOUSE|Q62277
Q5PR73	1	0.0		Diras2	DIRA2_MOUSE				NP_001019645	10090	DIRA2_MOUSE|Q5PR73
P05063	1	0.0		Aldoc	ALDOC_MOUSE				XP_006532096	10090	P05063|ALDOC_MOUSE
3866751	1	0.0		Syt2	3866751				NP_033333	10090	
102409979	1	0.0			102409979				XP_006499116	10090	
Q9DCS9	1	0.0		Ndufb10	NDUBA_MOUSE				NP_080960	10090	Q9DCS9|NDUBA_MOUSE
226510573	1	0.0			226510573				XP_006501641	10090	
P43024	1	0.0		Cox6a1	CX6A1_MOUSE				NP_031774	10090	CX6A1_MOUSE|P43024
A2ASQ1	1	0.0			AGRIN_MOUSE					10090	AGRIN_MOUSE|A2ASQ1
Q6PDS3-1	1	0.0		Sarm1	SARM1_MOUSE					10090	Q6PDS3-1|Q6PDS3|SARM1_MOUSE
Q8C8R3	1	0.0		Ank2	ANK2_MOUSE					10090	Q8C8R3|ANK2_MOUSE
P63028	1	0.0		Tpt1	TCTP_MOUSE				NP_033455	10090	TCTP_MOUSE|P63028
Q9Z0S9	1	0.0		Rabac1	PRAF1_MOUSE				NP_034391	10090	PRAF1_MOUSE|Q9Z0S9
Q6ZQ38	1	0.0		Cand1	CAND1_MOUSE				NP_082270	10090	CAND1_MOUSE|Q6ZQ38
106628779	1	0.0			106628779				XP_006523998	10090	
238678357	1	0.0			238678357				XP_006496558	10090	
E9PUL5	1	0.0		Prrt2	PRRT2_MOUSE				NP_001096033	10090	E9PUL5|PRRT2_MOUSE
P97492	1	0.0		Rgs14	RGS14_MOUSE				NP_058038	10090	RGS14_MOUSE|P97492
152568194	1	0.0			152568194				NP_038568	10090	
Q4ACU6-1	1	0.0		Shank3	SHAN3_MOUSE					10090	SHAN3_MOUSE|Q4ACU6|Q4ACU6-1
2005137	1	0.0		Gopc	2005137				NP_444417	10090	
172964819	1	0.0			172964819				XP_006504712	10090	
Q61627	1	0.0		Grid1	GRID1_MOUSE				NP_032192	10090	GRID1_MOUSE|Q61627
Q80Z38	1	0.0		Shank2	SHAN2_MOUSE					10090	Q80Z38|SHAN2_MOUSE
Q61626	1	0.0		Grik5	GRIK5_MOUSE	Grik5			NP_032194	10090	Q61626|GRIK5_MOUSE
262192267	1	0.0		Grid2ip	262192267				NP_579933	10090	
Q60591	1	0.0		Nfatc2	NFAC2_MOUSE				NP_035029	10090	NFAC2_MOUSE|Q60591
Q5D052	1	0.0		Homer1	Q5D052				NP_036112	10090	Q5D052_MOUSE|Q5D052
Q8BX57-1	1	0.0		Pxk	PXK_MOUSE					10090	Q8BX57|PXK_MOUSE|Q8BX57-1
Q8BX57-2	1	0.0		Pxk	Q8BX57-2					10090	Q8BX57-2
Q99LD8	1	0.0		Ddah2	DDAH2_MOUSE				XP_006524675	10090	Q99LD8|DDAH2_MOUSE
A2AAJ9	1	0.0			OBSCN_MOUSE					10090	OBSCN_MOUSE|A2AAJ9
P31725	1	0.0		S100a9	P31725				NP_001268781|NP_033140	10090	P31725|S10A9_MOUSE
36091432	1	0.0		Aqp4	36091432				XP_006525603	10090	
Q9QYG0	1	0.0		Ndrg2	NDRG2_MOUSE				XP_006534716	10090	Q9QYG0|NDRG2_MOUSE
4875525	1	0.0		Alcam	4875525				XP_006521777	10090	
Q9CPQ8	1	0.0		Atp5l	ATP5L_MOUSE				NP_038823	10090	Q9CPQ8|ATP5L_MOUSE
P62897	1	0.0		Cycs	CYC_MOUSE				NP_031834	10090	CYC_MOUSE|P62897
Q9D385	1	0.0		Arl2bp	AR2BP_MOUSE				NP_077153	10090	AR2BP_MOUSE|Q9D385
Q9CR16	1	0.0		Ppid	PPID_MOUSE				NP_080628	10090	PPID_MOUSE|Q9CR16
Q9CPQ1	1	0.0		Cox6c	COX6C_MOUSE				NP_444301	10090	COX6C_MOUSE|Q9CPQ1
Q06185	1	0.0		Atp5k	ATP5I_MOUSE				NP_031533	10090	Q06185|ATP5I_MOUSE
Q9D0M3	1	0.0		Cyc1	CY1_MOUSE				NP_079843	10090	CY1_MOUSE|Q9D0M3
P47857	1	0.0		Pfkm	PFKAM_MOUSE				XP_006520665	10090	PFKAM_MOUSE|P47857
Q91WS0	1	0.0		Cisd1	CISD1_MOUSE				NP_598768	10090	Q91WS0|CISD1_MOUSE
Q9D6R2	1	0.0		Idh3a	IDH3A_MOUSE				NP_083849	10090	Q9D6R2|IDH3A_MOUSE
Q9CQA3	1	0.0		Sdhb	SDHB_MOUSE				NP_075863	10090	Q9CQA3|SDHB_MOUSE
Q9CR68	1	0.0		Uqcrfs1	UCRI_MOUSE				NP_079986	10090	Q9CR68|UCRI_MOUSE
Q03265	1	0.0		Atp5a1	ATPA_MOUSE				NP_031531	10090	ATPA_MOUSE|Q03265
P12787	1	0.0		Cox5a	COX5A_MOUSE				NP_031773	10090	COX5A_MOUSE|P12787
Q60931	1	0.0		Vdac3	VDAC3_MOUSE				NP_035826	10090	Q60931|VDAC3_MOUSE
A2AKU9	1	0.0		Atp5c1	A2AKU9				XP_006497373	10090	A2AKU9|A2AKU9_MOUSE
Q9CQQ7	1	0.0		Atp5pb	AT5F1_MOUSE				XP_006500997	10090	AT5F1_MOUSE|Q9CQQ7
P56480	1	0.0		Atp5b	ATPB_MOUSE				NP_058054	10090	ATPB_MOUSE|P56480
Q62425	1	0.0		Ndufa4	NDUA4_MOUSE				NP_035016	10090	NDUA4_MOUSE|Q62425
P08249	1	0.0		Mdh2	MDHM_MOUSE				NP_032643	10090	MDHM_MOUSE|P08249
Q4FK74	1	0.0		Atp5d	ATPD_MOUSE				NP_079589	10090	Q4FK74|ATPD_MOUSE|Q4FK74_MOUSE
Q9DB77	1	0.0		Uqcrc2	QCR2_MOUSE				NP_080175	10090	QCR2_MOUSE|Q9DB77
O35857	1	0.0		Timm44	TIM44_MOUSE				NP_035722	10090	O35857|TIM44_MOUSE
Q8VDM4	1	0.0		Psmd2	PSMD2_MOUSE				NP_598862	10090	Q8VDM4|PSMD2_MOUSE
Q5FWK3	1	0.0		Arhgap1	RHG01_MOUSE				XP_006499309	10090	RHG01_MOUSE|Q5FWK3
E9Q401	1	0.0		Ryr2	RYR2_MOUSE				NP_076357	10090	RYR2_MOUSE|E9Q401
Q80SW1	1	0.0		Ahcyl1	SAHH2_MOUSE				NP_663517	10090	Q80SW1|SAHH2_MOUSE
9759065	1	0.0		Xntrpc	9759065				NP_035774	10090	
Q3U182	1	0.0		Crtc2	CRTC2_MOUSE	Crtc2			NP_083157	10090	CRTC2_MOUSE|Q3U182
Q80VH0	1	0.0		Bank1	BANK1_MOUSE				NP_001028522	10090	Q80VH0|BANK1_MOUSE
204075563	1	0.0			204075563				XP_006533197	10090	
140340193	1	0.0			140340193				XP_006501360	10090	
O35425	1	0.0		Bok	BOK_MOUSE					10090	O35425|BOK_MOUSE
P70227	1	0.0		Itpr3	ITPR3_MOUSE				NP_542120	10090	ITPR3_MOUSE|P70227
Q9CPV7	1	0.0		Zdhhc6	ZDHC6_MOUSE					10090	ZDHC6_MOUSE|Q9CPV7
Q4VAE6	1	0.0		Rhoa	RHOA_MOUSE				NP_058082|XP_006511696	10090	Q4VAE6|Q4VAE6_MOUSE|RHOA_MOUSE
356523274	1	0.0			356523274				NP_084241	10090	
192649431	1	0.0			192649431				XP_006498778	10090	
P28651	1	0.0		Car8	CAH8_MOUSE	Car8			NP_031618	10090	P28651|CAH8_MOUSE
207886107	1	0.0			207886107				XP_006543910	10090	
Q91UZ1	1	0.0		Plcb4	Q91UZ1				NP_038857	10090	Q91UZ1|Q91UZ1_MOUSE
P68134	1	0.0		Acta1	ACTS_MOUSE				NP_033736|NP_001258970	10090	ACTS_MOUSE|ACTS_PIG|P68134|ACTS_BOVIN|ACTS_CHICK|ACTS_HUMAN
Q91X78	1	0.0		Erlin1	ERLN1_MOUSE				XP_006527061	10090	Q91X78|ERLN1_MOUSE
126421116	1	0.0			126421116				XP_017174982	10090	
P61264	1	0.0		Stx1b	STX1B_MOUSE				NP_077725	10090	P61264|STX1B_MOUSE
P05132	1	0.0		Prkaca	KAPCA_MOUSE				NP_032880	10090	KAPCA_MOUSE|P05132
Q8BNY6	1	0.0		Ncs1	NCS1_MOUSE				NP_062655	10090	NCS1_MOUSE|Q8BNY6
A2AI17	1	0.0		Grin1	A2AI17				XP_006497788	10090	A2AI17|A2AI17_MOUSE
Q9JJA9	1	0.0		Tamalin	GRASP_MOUSE				NP_062391	10090	Q9JJA9|GRASP_MOUSE
Q9QWW1-2	1	0.0		Homer2	Q9QWW1-2				XP_133550	10090	Q9QWW1-2
E9Q4F9	1	0.0		Homer2	E9Q4F9				XP_006540972	10090	E9Q4F9_MOUSE|E9Q4F9
Q3UVX5	1	0.0		Grm5	GRM5_MOUSE				XP_006507246	10090	GRM5_MOUSE|Q3UVX5
P58389	1	0.0		Ptpa	PTPA_MOUSE				NP_620087	10090	PTPA_MOUSE|P58389
P09671	1	0.0		Sod2	SODM_MOUSE				NP_038699	10090	P09671|SODM_MOUSE
Q64337	1	0.0		Sqstm1	SQSTM_MOUSE				NP_035148	10090	Q64337|SQSTM_MOUSE
237476405	1	0.0		Xylb	237476405				XP_006511973	10090	
208576339	1	0.0		Mrpl58	208576339				NP_081005	10090	
P19783	1	0.0		Cox4i1	COX41_MOUSE				NP_001280488	10090	COX41_MOUSE|P19783
207514938	1	0.0		Elavl2	207514938				XP_006502849	10090	
Q80ZK9	1	0.0		Wdtc1	WDTC1_MOUSE				NP_955010	10090	WDTC1_MOUSE|Q80ZK9
Q8R5L1	1	0.0		C1qbp	Q8R5L1				NP_031599	10090	Q8R5L1_MOUSE|Q8R5L1
Q60900	1	0.0		Elavl3	ELAV3_MOUSE				NP_034617	10090	Q60900|ELAV3_MOUSE
Q8R4A3	1	0.0		Dact1	DACT1_MOUSE	Dact1			NP_001177395|NP_067507	10090	Q8R4A3|DACT1_MOUSE
Q9WTL8	1	0.0		Arntl	BMAL1_MOUSE					10090	Q9WTL8|BMAL1_MOUSE
Q7TN08	1	0.0		Dact2	DACT2_MOUSE	Dact2			NP_766414	10090	DACT2_MOUSE|Q7TN08
Q9DBX2	1	0.0		Pdcl	PHLP_MOUSE				NP_080452	10090	Q9DBX2|PHLP_MOUSE
Q9JMF3	1	0.0		Gng13	GBG13_MOUSE	Gng13			NP_071867	10090	Q9JMF3|GBG13_MOUSE
1640682	1	0.0		Dync1i1	1640682				XP_017176869	10090	
P25388	1	0.0		Rack1	RACK1_CHICK				NP_032169	10090	RACK1_CHICK|RACK1_BOVIN|RACK1_MOUSE|RACK1_PIG|RACK1_HUMAN|P25388

Q6NZM9	1	0.0		Hdac4	HDAC4_MOUSE				NP_997108	10090	HDAC4_MOUSE|Q6NZM9
Q9DAS9	1	0.0		Gng12	GBG12_MOUSE				XP_006505605	10090	Q9DAS9|GBG12_MOUSE
P21279	1	0.0		Gnaq	GNAQ_MOUSE				NP_032165	10090	GNAQ_MOUSE|Q3UHH5_MOUSE|P21279
P61953	1	0.0		Gng11	GBG11_MOUSE				NP_079607	10090	GBG11_MOUSE|P61953
B2RSH2	1	0.0		Gnai1	GNAI1_MOUSE				NP_034435	10090	GNAI1_MOUSE|B2RSH2
P28658	1	0.0		Atxn10	ATX10_MOUSE				NP_058539	10090	P28658|ATX10_MOUSE
P21278	1	0.0		Gna11	GNA11_MOUSE				NP_034431	10090	GNA11_MOUSE|P21278
114182826	1	0.0		Kctd12	114182826				NP_808383	10090	
Q64321	1	0.0		Zbtb7b	ZBT7B_MOUSE				NP_033591	10090	ZBT7B_MOUSE|Q64321
P29798	1	0.0		Gng3	GBG3_BOVIN				NP_034446	10090	P29798|GBG3_BOVIN|GBG3_HUMAN|GBG3_MOUSE
200950923	1	0.0		Cnksr2	200950923				XP_011246128	10090	
94230255	1	0.0			94230255				XP_006513688	10090	
P08752	1	0.0		Gnai2	GNAI2_MOUSE				NP_032164	10090	GNAI2_MOUSE|P08752
Q80ZX8	1	0.0		Spag1	SPAG1_MOUSE				NP_036161	10090	Q80ZX8|SPAG1_MOUSE
P27601	1	0.0		Gna13	GNA13_MOUSE				NP_034433	10090	GNA13_MOUSE|P27601
Q8VDC0	1	0.0		Lars2	SYLM_MOUSE				NP_694808	10090	Q8VDC0|SYLM_MOUSE
Q8VBY2	1	0.0		Camkk1	KKCC1_MOUSE				NP_061371	10090	Q8VBY2|KKCC1_MOUSE
Q14CH7	1	0.0		Aars2	SYAM_MOUSE				NP_941010	10090	Q14CH7|SYAM_MOUSE
Q3U2G2	1	0.0		Hspa4	Q3U2G2				NP_032326	10090	Q3U2G2|Q3U2G2_MOUSE
Q9EPU0	1	0.0		Upf1	RENT1_MOUSE				XP_006509667	10090	Q9EPU0|RENT1_MOUSE
Q6ZQ58	1	0.0		Larp1	LARP1_MOUSE				NP_082727	10090	LARP1_MOUSE|Q6ZQ58
G3X9J0	1	0.0		Sipa1l3	SI1L3_MOUSE				NP_001074497	10090	G3X9J0|SI1L3_MOUSE
226583887	1	0.0			226583887				XP_017169361	10090	
O35929	1	0.0		Rem1	REM1_MOUSE	Rem1			NP_033073	10090	O35929|REM1_MOUSE
Q9EPK5	1	0.0		Wwtr1	WWTR1_MOUSE				NP_598545	10090	Q9EPK5|WWTR1_MOUSE
5118417	1	0.0		Hnrnpk	5118417				XP_017170885	10090	
180542602	1	0.0			180542602				XP_006522424	10090	
Q8BFP9	1	0.0		Pdk1	PDK1_MOUSE				NP_766253	10090	Q8BFP9|PDK1_MOUSE
419877456	1	0.0			419877456				XP_006500876	10090	
H7BX26	1	0.0		Cep170	H7BX26				NP_001093107	10090	H7BX26_MOUSE|H7BX26
Q3UJU9	1	0.0		Rmdn3	RMD3_MOUSE				NP_001028308	10090	Q3UJU9|RMD3_MOUSE
266785558	1	0.0			266785558				XP_006501092	10090	
Q04447	1	0.0		Ckb	KCRB_MOUSE				NP_067248	10090	Q04447|KCRB_MOUSE
O08579	1	0.0		Emd	EMD_MOUSE				NP_031953	10090	EMD_MOUSE|O08579
Q61081	1	0.0		Cdc37	CDC37_MOUSE				NP_058022	10090	Q61081|CDC37_MOUSE
P10107	1	0.0		Anxa1	ANXA1_MOUSE				NP_034860	10090	P10107|ANXA1_MOUSE
O35841	1	0.0		Api5	API5_MOUSE				NP_031492	10090	O35841|API5_MOUSE
F6ZDS4	1	0.0		Tpr	TPR_MOUSE				NP_598541	10090	TPR_MOUSE|F6ZDS4
P68510	1	0.0		Ywhah	1433F_BOVIN				NP_035868	10090	P68510|1433F_BOVIN|1433F_MOUSE|1433F_RAT
439433617	1	0.0		Braf	439433617				NP_647455	10090	
1684059	1	0.0		Crk	1684059				XP_006532188	10090	
P11499	1	0.0		Hsp90ab1	HS90B_MOUSE				NP_032328	10090	HS90B_MOUSE|P11499|Q71LX8_MOUSE
O70591	1	0.0		Pfdn2	PFD2_MOUSE				NP_035200	10090	PFD2_MOUSE|O70591
Q8K596	1	0.0		Slc8a2	NAC2_MOUSE				NP_683748	10090	NAC2_MOUSE|Q8K596
P70265	1	0.0		Pfkfb2	P70265					10090	F262_MOUSE|P70265
Q8R081	1	0.0		Hnrnpl	HNRPL_MOUSE				NP_796275	10090	HNRPL_MOUSE|Q8R081
179561655	1	0.0			179561655				XP_006496631	10090	
121784890	1	0.0			121784890				XP_006526716	10090	
P07901	1	0.0		Hsp90aa1	HS90A_MOUSE				NP_034610	10090	P07901|HS90A_MOUSE
254200261	1	0.0			254200261				XP_006498286	10090	
144711816	1	0.0			144711816				XP_006526432	10090	
140398994	1	0.0			140398994				XP_006538545	10090	
Q8VEK3	1	0.0		Hnrnpu	HNRPU_MOUSE				NP_058085	10090	Q8VEK3|HNRPU_MOUSE
A8IP69	1	0.0		Ywhag	1433G_MOUSE				NP_061359	10090	A8IP69_MOUSE|1433G_MOUSE|A8IP69
Q9JKF1	1	0.0		Iqgap1	IQGA1_MOUSE				NP_057930	10090	Q9JKF1|IQGA1_MOUSE
175186800	1	0.0			175186800				XP_006510099	10090	
ENSMUSG00000054640	1	0.0			ENSMUSG00000054640	Slc8a1			NP_001106269|NP_001273613|NP_035536	10090	
Q9JIP7	1	0.0			Q9JIP7	Slc15a1			NP_444309	10090	Q9JIP7|S15A1_MOUSE
Q99JW5	1	0.0			Q99JW5	Epcam			NP_032558	10090	Q99JW5
P23611	1	0.0			P23611	Irf8			NP_032346	10090	P23611
P22091	1	0.0			TAL1_MOUSE	Tal1			NP_001274317|NP_035657	10090	P22091|TAL1_MOUSE
Q9JKN6	1	0.0			Q9JKN6	Nova1			NP_067336	10090	Q9JKN6
Q6Q899	1	0.0			Q6Q899	Ddx58			NP_766277	10090	Q6Q899
Q9QZH6	1	0.0			Q9QZH6	Ecsit			NP_036159|NP_001240827|NP_001240826	10090	Q9QZH6
Q9DC51	1	0.0			Q9DC51	Gnai3			NP_034436	10090	Q9DC51
Q8C985	1	0.0			NRX3B_MOUSE	Nrxn3			NP_766132|NP_001185516|NP_001239003	10090	Q8C985|NRX3B_MOUSE|Q6P9K9
ENSMUSG00000033981	1	0.0			ENSMUSG00000033981	Gria2			NP_001077275|NP_001034284|NP_038568	10090	
Q9R244	1	0.0			TRPC2_MOUSE	Xndc1			NP_001103367|NP_035774|NP_001273618|NP_001273619	10090	Q9R244|TRPC2_MOUSE
ENSMUSG00000009995	1	0.0			ENSMUSG00000009995	Taz			NP_001277667|NP_001229545|NP_001229544|NP_852657|NP_001167018	10090	
ENSMUSG00000032702	1	0.0			ENSMUSG00000032702	Kank1			NP_852069	10090	
ENSMUSG00000002496	1	0.0			ENSMUSG00000002496	Tsc2			NP_001034452|NP_001273645|NP_001273647|NP_035777	10090	
P08505	1	0.0			P08505	Il6			NP_112445	10090	P08505
Q60769	1	0.0			TNAP3_MOUSE	Tnfaip3			NP_033423|NP_001159874	10090	TNAP3_MOUSE|Q60769
P30882	1	0.0			P30882	Ccl5			NP_038681	10090	P30882
Q9WV17	1	0.0			Gabbr1					10090	Q6PGJ2|Gabbr1|Q9WV16|Q9WV17|Q9WU48|Q9WV15
Q8C6U5	1	0.0			Dnajc11					10090	Q8C1Z4|Dnajc11|Q8C6U5|A2A8C9|Q8BP83
Q7TPR4	1	0.0			F-actin cross-linking protein					10090	F-actin cross-linking protein|Q7TPR4|Alpha-actinin cytoskeletal isoform|Actn1|Non-muscle alpha-actinin-1
A2AIC3	1	0.0			Snap					10090	Q9BR45|A2AIC2|A2AIC3|P36974|Q96FM2|Snap|P70558|Q8IXK3|P13795|P70557|Snap25|Synaptosomal-associated 25 kDa protein|Super protein
Q9EQF8	1	0.0			Msy4					10090	Msy4|Q9EQF7|Q9EQF8|DNA-binding protein A|Ybx3|Csda|Cold shock domain-containing protein A|Q80WG4|Y-box protein 3
B7ZNT6	1	0.0			Z-band alternatively spliced PDZ-motif protein					10090	Z-band alternatively spliced PDZ-motif protein|Q811P5|Protein cypher|Q9D130|Q811P2|Ldb3|Q811P3|Q811P4|Q9WVH1|Q9WVH2|Q6A038|Q9JKS3|B2RSB0|Protein oracle|B7ZNT6|Q9R0Z1|Kiaa0613
Q3UEH1	1	0.0			Fructose-1,6-bisphosphatase isozyme 3					10090	Fructose-1,6-bisphosphatase isozyme 3|Q3UEH1|Fbp|Liver FBPase|P97323|D-fructose-1,6-bisphosphate 1-phosphohydrolase 1|Fbp1|Fbp3
Q78KK2	1	0.0			ILK2					10090	p59ILK|ILK-1|ILK-2|ILK2|Ilk|Q78KK2|ILK1|59 kDa serine/threonine-protein kinase|Beta-integrin-linked kinase
Q8VI44	1	0.0			Flii					10090	Flii|Q8K095|Q8VI44|Fli1|Fliih
Q9CQE6	1	0.0			Anti-silencing function protein 1 homolog A					10090	Anti-silencing function protein 1 homolog A|Q9CQE6|Asf1a
Q9ET11	1	0.0			CFTR-associated ligand					10090	CFTR-associated ligand|PDZ protein interacting specifically with TC10|Q920R1|Q8BSV4|Gopc|Q9ET11|Cal
Q99J03	1	0.0			Becn1					10090	Becn1|Q99J03|Coiled-coil myosin-like BCL2-interacting protein
G5E8E7	1	0.0			Testis-enriched protein tyrosine phosphatase					10090	G5E8E7|Testis-enriched protein tyrosine phosphatase|Ptpn4
Q64330	1	0.0			Double minute 2 protein					10090	Double minute 2 protein|RING-type E3 ubiquitin transferase Mdm2|Q64330|Q91XK7|Q61040|Mdm2|p53-binding protein Mdm2|Oncoprotein Mdm2
A2RT52	1	0.0			Akt1s1					10090	Akt1s1|A2RT52|Pras
Q9D4H3	1	0.0			Rptor					10090	Rptor|Q8C9W9|Q9D4H3|p150 target of rapamycin (TOR)-scaffold protein|Q8CDY8|Q8CBY4|Raptor
Q91WH1	1	0.0			Craf					10090	Q58E75|Q91WH1|Craf|Proto-oncogene c-RAF|Q99N58|Raf-1|Raf1|Q3UR68|Q9QUU8
Q0VGQ5	1	0.0			Pik3ca					10090	Q0VGQ5|Pik3ca|Phosphoinositide-3-kinase catalytic alpha polypeptide|Phosphatidylinositol 4,5-bisphosphate 3-kinase 110 kDa catalytic subunit alpha|Serine/threonine protein kinase PIK3CA
P97723	1	0.0			Tsc2					10090	Q9WUF6|Q61007|Q61008|Tsc2|P97723|P97724|P97725|P97727|Tuberous sclerosis 2 protein homolog
Q3U0N1	1	0.0			Rab1b					10090	Rab1b|Q3U0N1
Q9JMB9	1	0.0			PEX2-related protein					10090	PEX2-related protein|PEX5-like protein|Peroxin-5-related protein|Pex5r|Pex2|Tetratricopeptide repeat-containing Rab8b-interacting protein|Q9JMB9|Pex5l|Q8CA31|Q9DAA1|Pxr2
Q9Z216	1	0.0			Homer1					10090	Q9Z216|Homer1|Vesl1|VASP/Ena-related gene up-regulated during seizure and LTP 1|Q9Z0E9|Q8K3E1|Q8K4M8
Q8BRI9	1	0.0			Sterile alpha and TIR motif-containing protein 1					10090	Q5SYG5|Sterile alpha and TIR motif-containing protein 1|Q5SYG6|NADP(+) hydrolase SARM1|Sarm1|Q8BRI9|Q6A054|Q6SZW0|Kiaa0524
Q3TSL4	1	0.0			Rab3a					10090	Rab3a|P05713|Q3TSL4

Q3TDB4	1	0.0			PRA1 family protein 1					10090	Pra1 | Praf1 | PRA1 family protein 1 | Prenylin | Q3TDB4 | Rabac1 | Pra
Q9R1S0	1	0.0			Eppb9					10090	Q9R1S0 | B9d1 | Eppb9 | Endothelial precursor cells protein B9
Q3TSQ6	1	0.0			Rab4a					10090	Rab4a | Rab4 | Q3TSQ6
Q8CHX2	1	0.0			Atp6v1a					10090	Q3U5W3 | Q3US31 | Atp6v1a | Q3TKS0 | Atp6a1 | Atp6v1a1 | Q3U777 | Q8CHX2 | Atp6a2 | Vacuolar proton pump subunit alpha | V-ATPase 69 kDa subunit | Q3UDZ9
Q99K32	1	0.0			App					10090	App | Alzheimer disease amyloid A4 protein homolog | P97487 | Amyloidogenic glycoprotein | P97942 | Amyloid precursor protein | AD1 | Q99K32 | A4 | Amyloid-beta (A4) precursor protein | Alzheimer disease amyloid protein | Amyloid-beta A4 protein | ABPP
Q3UQW0	1	0.0			Hmgic					10090	Hmgic | Q3UQW0 | Hmga2 | High mobility group AT-hook protein 2
Q9D6D3	1	0.0			Gria2					10090	Q9D6D3 | Gria2 | Q8BG69 | Q61604 | Q8BXU3 | Q61605 | GluR-B | AMPA-selective glutamate receptor 2 | Glur2 | Glutamate receptor ionotropic, AMPA 2 | GluR-K2
Q3UW92	1	0.0			Calcium/calmodulin-dependent serine protein kinase					10090	O70588 | Calcium/calmodulin-dependent serine protein kinase | Q3UW92 | A2ADP8 | Lin-2 | A2ADP9 | Cask | A2ADQ4
Q8CCU3	1	0.0			Dmxl2					10090	B0V2P4 | Dmxl2 | Q3TNY5 | Q8CCU3 | Kiaa0856 | Rabconnectin-3 | Q69ZX5
Q9JK89	1	0.0			Thymus-expressed novel gene 3 protein					10090	Thymus-expressed novel gene 3 protein | Thyex3 | Dapper antagonist of catenin 1 | Q9JK89 | MDpr1 | Q8BP49 | Dact1 | Frodo homolog | Q80VG9
Q8BHZ7	1	0.0			Dapper antagonist of catenin 2					10090	Q6PDF4 | Dapper antagonist of catenin 2 | Q8BHZ7 | Frd2 | Dpr2 | Dact2
lncrna_ kcnq1ot1	1	0.0			lncrna_ kcnq1ot1					10090	
Q9CY43	1	0.0			Dystrobrevin-binding protein 1					10090	Q80ZN4 | Q3TWK1 | Dystrobrevin-binding protein 1 | Q9CY43 | Dysbindin-1 | Bloc1s8 | Hermansky-Pudlak syndrome 7 protein homolog | Sdy | Dtnbp1 | Q6WXQ1 | Biogenesis of lysosome-related organelles complex 1 subunit 8
Q9ES15	1	0.0			Arc					10090	Arc | Activity-regulated gene 3.1 protein | Q9ES15
Q6ZWN4	1	0.0			Camk2a					10090	Q6ZWN4 | Camk2a | Q61284
Q9R181	1	0.0			Cyfip2					10090	Q9R181 | Q3UHS8 | Cyfip2 | Pir121 | Q8CHA9 | p53-inducible protein 121 | Q3UH21 | Q924D3 | Kiaa1168 | Q8BSW0
Q9QWF1	1	0.0			Neurl					10090	Q91ZT6 | Neurl | RING-type E3 ubiquitin transferase NEURL1 | Q9QWF1 | Neurl1 | Neuralized-like protein 1A | Q9CWF1 | Neurl1a | Neuralized1
Q9WUA7	1	0.0			Fxr1					10090	Fxr1 | Q9R1E3 | Q9R1E2 | Fxr1h | Q8VCU4 | Q9R1E5 | Q9R1E4 | Q9WUA7 | Q9WUA8 | Q9WUA9
E9PUJ6	1	0.0			Mycbp2					10090	Mycbp2 | Protein associated with Myc | E9PUJ6 | Phr1 | Q6PCM8 | Pam/highwire/rpm-1 protein | Myc-binding protein 2 | Protein Magellan | Pam
O35668-2	1	0.0			Hap1					10090	O35668-2 | Hap1 | Short
O35668-1	1	0.0			Long					10090	O35668-1 | Long | Hap1
Q3TA52	1	0.0			Crtc2					10090	Crtc2 | Q3TA52 | Torc2 | Q8BH09 | Transducer of regulated cAMP response element-binding protein 2
A2AJ84	1	0.0			Ncs1					10090	Ncs1 | Frequenin homolog | Freq | A2AJ84
Q7TSZ0	1	0.0			Hspa9a					10090	Q9CQ05 | Hspa9a | Q3UVN1 | p66 MOT | 75 kDa glucose-regulated protein | Hsp74 | Q3V015 | Q3TW93 | Hspa9 | Mortalin | Peptide-binding protein 74 | Q7TSZ0 | Grp75 | Heat shock 70 kDa protein 9
Q61311	1	0.0			Aac2					10090	Aac2 | ADP,ATP carrier protein 2 | Ant2 | Slc25a5 | Adenine nucleotide translocator 2 | Solute carrier family 25 member 5 | Q61311
loc97081_mouse_prote	1	0.0			loc97081_mouse_prote					10090	
Q4JFC7	1	0.0			Ptprs					10090	Q64503 | Ptprs | Q3TXC9 | Receptor-type tyrosine-protein phosphatase sigma | Q5XJV4 | Q4JFC7 | Q7TT17 | PTPNU-3 | Q3TEC3 | Q64699
Q8BGG3	1	0.0			Sfxn5					10090	Q8BGG3 | Sfxn5
P47933	1	0.0			Rps3					10090	Rps3 | P17073 | P47933
Q8R0C8	1	0.0			Bdh1					10090	Q8BK53 | Q3UJS9 | Bdh1 | Q3UL45 | 3-hydroxybutyrate dehydrogenase | Bdh | Q8R0C8
Q8BYN8	1	0.0			Ttc9b					10090	Q8BYN8 | Ttc9b
Q3U793	1	0.0			Q3U793					10090	Q3U793 | C1qb
Q14AI5	1	0.0			Slc25a18					10090	Slc25a18 | Gc2 | Solute carrier family 25 member 18 | Q14AI5 | B2RPU3 | Glutamate/H(+) symporter 2
Q3TG26	1	0.0			Tubb2b					10090	Tubb2b | Q3TG26
A6H6A8	1	0.0			Gapdh					10090	Q3TUI2 | Q4V783 | Q3THM2 | Q3UMT2 | Q569X5 | Q5U410 | Q0QEU0 | Gapdh | Q569X2 | Peptidyl-cysteine S-nitrosylase GAPDH | A6H6A8 | Gapd
Q3V269	1	0.0			Hnrnpa1					10090	Hnrnpa1 | Single-strand-binding protein | Topoisomerase-inhibitor suppressed | Hnrpa1 | Fli-2 | P97312 | Helix-destabilizing protein | Q3V269 | hnRNP core protein A1 | HDP-1 | Tis
Q99KU5	1	0.0			Eef1a1					10090	Q61511 | Q8BMB8 | Eef1a1 | Eukaryotic elongation factor 1 A-1 | Q99KU5 | Q8BVS8 | Elongation factor Tu | Q6ZWN2 | Eef1a
Q8C1Z2	1	0.0			Sfxn3					10090	Q8C1Z2 | Q544M7 | Sfxn3
Q8CCH1	1	0.0			Vim					10090	O08704 | Vim | Q8CCH1
Q9CZG4	1	0.0			Sfxn1					10090	Q3UB44 | Q9CZG4 | Sfxn1
Q9D785	1	0.0			Eci2					10090	Q99M61 | Q9D785 | Eci2 | Peroxisomal 3,2-trans-enoyl-CoA isomerase | Delta(3),delta(2)-enoyl-CoA isomerase | Peci | Dodecenoyl-CoA isomerase
Q9QVT5	1	0.0			Brain protein D3					10090	Brain protein D3 | Precerebellin | P28655 | Cbln1 | Q9QVT5
Q91VP2	1	0.0			Plat					10090	Q6P7U0 | Plat | Q91VP2
G3UWZ9	1	0.0			Neurexin-1-alpha					10090	Q8CHE6 | Neurexin-1-alpha | G3UWZ9 | O88722 | Nrxn1 | Q80Y87 | Kiaa0578 | Neurexin I-alpha
Q9QWV7	1	0.0			Synapsin II					10090	Synapsin II | Q6NZR0 | Syn2 | Q9QWV7
Q3TRY1	1	0.0			Solute carrier family 25 member 22					10090	Solute carrier family 25 member 22 | Gc1 | Q3TRY1 | Slc25a22 | Glutamate/H(+) symporter 1
Q69ZY7	1	0.0			FAT tumor suppressor homolog 2					10090	Q3V1D4 | FAT tumor suppressor homolog 2 | Q69ZY7 | Fat2 | Fath2 | Kiaa0811
Q921T4	1	0.0			PAF 2-acylhydrolase					10090	PAF 2-acylhydrolase | Q921T4 | 1-alkyl-2-acetylglycerophosphocholine esterase | Pafah | Q8BKM3 | 2-acetyl-1-alkylglycerophosphocholine esterase | LDL-associated phospholipase A2 | Pla2g7
Q8C9L8	1	0.0			Eln					10090	Q8C9L8 | Eln | Tropoelastin
Q9WTY3	1	0.0			Palmitoyl-protein hydrolase 1					10090	Palmitoyl-protein hydrolase 1 | Ppt | Q9D681 | Q9WTY3 | Ppt1 | Cln1
Q8K1A1	1	0.0			Dynamin-related protein 1					10090	Dynamin-related protein 1 | Q8BQ64 | Q8CGD0 | Dynamin family member proline-rich carboxyl-terminal domain less | Q8BNQ5 | Q8K1A1 | Dnm1l | Drp1
Q3U509	1	0.0			Protein DJ-1					10090	Q3THB9 | O88306 | Q3U509 | Protein DJ-1 | Parkinsonism-associated deglycase | Park7 | Protein/nucleic acid deglycase DJ-1 | Maillard deglycase
Q8CHJ3	1	0.0			Zap70					10090	Zap70 | Syk-related tyrosine kinase | Zap-70 | P97455 | Q8CHJ3 | 70 kDa zeta-chain associated protein | Q80VV2 | Srk
Q9EQF3	1	0.0			Cbp					10090	Cbp | Q8BFS1 | Pag | Transmembrane phosphoprotein Cbp | Csk-binding protein | Q9EQF3 | Pag1
Q8CE05	1	0.0			Cxn-43					10090	Cxn-43 | Gja1 | Q8CE05 | Gap junction 43 kDa heart protein | Q544I7 | Connexin-43
E9QN40	1	0.0			Itga5					10090	E9QN40 | Itga5 | Integrin alpha-F | VLA-5 | Fibronectin receptor subunit alpha | CD49 antigen-like family member E
A2AKD7	1	0.0			Snta1					10090	A2AKD7 | Snta1
Q99K63	1	0.0			Lap1					10090	Q99K63 | Lap1 | Q91YH8 | Lap-1 | Alanyl aminopeptidase | Anpep | Membrane protein p161 | Microsomal aminopeptidase | Aminopeptidase M
Q9DC56	1	0.0			Actr3					10090	Actr3 | Q9DC56 | Actin-like protein 3 | Arp3
O35989	1	0.0			Ether-a-go-go-related gene potassium channel 1					10090	Ether-a-go-go-related gene potassium channel 1 | O35221 | Voltage-gated potassium channel subunit Kv11.1 | Merg1 | O35220 | Erg | O35989 | Kcnh2
Q99242	1	0.0			Calcium channel, L type, alpha-1 polypeptide, isoform 1, cardiac muscle					10090	Calcium channel, L type, alpha-1 polypeptide, isoform 1, cardiac muscle | Voltage-gated calcium channel subunit alpha Cav1.2 | Cchl1a1 | MELC-CC | Cach2 | Cacna1c | Cacn2 | Cacnl1a1 | Q99242 | Q04476 | Mouse brain class C | Q61242
Q542K7	1	0.0			Rps23					10090	Q542K7 | P39028 | Rps23
P32233	1	0.0			Translation factor GTPase DRG1					10090	Translation factor GTPase DRG1 | Neural precursor cell expressed developmentally down-regulated protein 3 | Nedd-3 | Nedd3 | Drg1 | Drg | P32233
Q9CX86	1	0.0			Hnrnpa0					10090	Q9CX86 | Hnrnpa0 | Hnrpa0
P13641	1	0.0			snRNP core protein D1					10090	snRNP core protein D1 | Snrpd1 | Q3UYM0 | Sm-D autoantigen | P13641
Q9DBR5	1	0.0			aarF domain-containing protein kinase 3					10090	Q8K0M1 | Q9DBR5 | aarF domain-containing protein kinase 3 | Q9D657 | Coenzyme Q protein 8A | Q9JHR3 | Coq8a | Adck3 | Chaperone activity of bc1 complex-like | Cabc1
Q3UL55	1	0.0			GAP SH3 domain-binding protein 2					10090	Q3UL55 | GAP SH3 domain-binding protein 2 | G3bp2 | Q9R1B8
P02468	1	0.0			Laminin-9 subunit gamma					10090	Laminin-9 subunit gamma | Laminin-3 subunit gamma | S-laminin subunit gamma | Laminin-4 subunit gamma | Laminin-2 subunit gamma | Laminin-11 subunit gamma | Laminin B2 chain | Laminin-1 subunit gamma | Laminin-7 subunit gamma | Lamb-2 | Lamc-1 | Laminin-10 subunit gamma | Laminin-8 subunit gamma | Lamc1 | P02468 | Laminin-6 subunit gamma
Q08857	1	0.0			PAS IV					10090	Q08857 | PAS IV | Platelet glycoprotein IV | Cd36 | PAS-4 | Glycoprotein IIIb
Q9Z1T0	1	0.0			Abcp					10090	Abcp | Bcrp1 | Urate exporter | Breast cancer resistance protein 1 homolog | ATP-binding cassette sub-family G member 2 | Q9R004 | Q9Z1T0 | Abcg2
Q64360	1	0.0			Tnnt2					10090	Tnnt2 | Q64360 | Cardiac muscle troponin T | Q64377
Q64235	1	0.0			Acadm					10090	Acadm | Q64235
Q3U987	1	0.0			Aspartyl-tRNA synthetase					10090	Aspartyl-tRNA synthetase | Q3U987 | Dars | Dars1
Q58EA4	1	0.0			Rpl24					10090	P38663 | Rpl24 | Q58EA4
U2af65	1	0.0			U2af65					10090	U2 snRNP auxiliary factor large subunit | U2af2 | U2 auxiliary factor 65 kDa subunit | U2af65
Q3UYY7	1	0.0			Erythrocyte ankyrin					10090	Q61302 | Q61303 | Erythrocyte ankyrin | Q3TZ35 | Ank-1 | P70440 | P97446 | P97941 | Q3UYY7 | Ank1 | Q78E45 | Q3UHP2 | Q3UH42
Q61336	1	0.0			Bap					10090	O89075 | Bap | Q61336 | Bcap37 | Repressor of estrogen receptor activity | Phb2 | B-cell receptor-associated protein BAP37 | Rea
Q8R073	1	0.0			Ndufs3					10090	Q8R073 | Ndufs3 | Complex I-30kD | Q8BTZ3 | NADH-ubiquinone oxidoreductase 30 kDa subunit
O35479	1	0.0			RNA binding motif protein, X chromosome retrogene					10090	RNA binding motif protein, X chromosome retrogene | O35479 | Rbmxl1 | Rbmxrt | Heterogeneous nuclear ribonucleoprotein G-like 1
P09541	1	0.0			MLC1EMB					10090	Myl4 | Myla | MLC1EMB | P09541 | Mlc1a | Myosin light chain 1, atrial/fetal isoform
P02383	1	0.0			Rps26					10090	Rps26 | P02383 | P70394 | Q06722
P17426	1	0.0			Plasma membrane adaptor HA2/AP2 adaptin alpha A subunit					10090	Plasma membrane adaptor HA2/AP2 adaptin alpha A subunit | Alpha-adaptin A | Adaptor-related protein complex 2 subunit alpha-1 | Adtaa | Adaptor protein complex AP-2 subunit alpha-1 | Ap2a1 | 100 kDa coated vesicle protein A | Alpha1-adaptin | Clathrin assembly protein complex 2 alpha-A large chain | Clapa1 | P17426
Q9EQK1	1	0.0			IDP					10090	Q8C2R9 | IDP | Q9EQK1 | Idh2 | Oxalosuccinate decarboxylase | NADP(+)-specific ICDH | ICD-M
Q6P919	1	0.0			Tufm					10090	Q6P919 | Q497E7 | Tufm
Q9DCM8	1	0.0			Phospholipase A1					10090	Phospholipase A1 | Q05956 | Q542L4 | Q9D3M9 | Q9DCM8 | Lpl
Q9JJI8	1	0.0			Rpl38					10090	Q9JJI8 | Rpl38
P18760	1	0.0			Cfl1					10090	Cfl1 | P18760 | Cofilin, non-muscle isoform
Q8K2K9	1	0.0			Suclg2					10090	Q3TJQ5 | Q3T9B8 | Q66JT3 | Suclg2 | Q3TK63 | GTP-specific succinyl-CoA synthetase subunit beta | Succinyl-CoA synthetase beta-G chain | Q8K2K9 | Q80VV1 | Q7TMY3

Q99K47	1	0.0			Fga					10090	Fga | Q99K47
Q8BHF8	1	0.0			Hnrpa3					10090	Q8BHF8 | Hnrpa3 | Hnrnpa3
P60335	1	0.0			Pcbp1					10090	Pcbp1 | Heterogeneous nuclear ribonucleoprotein E1 | P60335 | Alpha-CP1
Q91YT0	1	0.0			Ndufv1					10090	Ndufv1 | Complex I-51kD | NADH-ubiquinone oxidoreductase 51 kDa subunit | Q91YT0
Q3TIT9	1	0.0			Acaa2					10090	Q3TIT9 | Acaa2 | Acetyl-CoA acetyltransferase | Acyl-CoA hydrolase, mitochondrial | Acetyl-CoA acyltransferase | Beta-ketothiolase | Q8JZR8 | Mitochondrial 3-oxoacyl-CoA thiolase
Q9D617	1	0.0			Fundc2					10090	Q80YD9 | Fundc2 | Hepatitis C virus core-binding protein 6 | Hcbp6 | Q9D617 | Q80VT2 | Q8BSM8
Q96J62	1	0.0			Hnrnpk					10090	Q60577 | Q922Y7 | Q15671 | Q07244 | Q96J62 | Hnrnpk | Hnrpk | Q8BGQ8
Q9DCI8	1	0.0			Succinyl-CoA synthetase subunit alpha					10090	Q9DBA3 | Succinyl-CoA synthetase subunit alpha | Suclg1 | Q8C2C3 | Q9DCI8
Q9CZ98	1	0.0			Q9CZ98					10090	Q99LX2 | U2 snRNP auxiliary factor small subunit | Q9CZ98 | U2af1 | Q564E4 | U2 auxiliary factor 35 kDa subunit
Q3TNA8	1	0.0			Complex I-49kD					10090	Complex I-49kD | Ndufs2 | Q3UJX7 | Q99L23 | Q3U5Y9 | Q3TNA8 | NADH-ubiquinone oxidoreductase 49 kDa subunit
P53986	1	0.0			Slc16a1					10090	Slc16a1 | Mct1 | Solute carrier family 16 member 1 | P53986
Q80W28	1	0.0			Ant4					10090	Q80W28 | Ant4 | Sperm flagellar energy carrier protein | Solute carrier family 25 member 31 | Sfec | ADP,ATP carrier protein 4 | Slc25a31 | Aac4 | Adenine nucleotide translocator 4
Q9QYB6	1	0.0			Addl					10090	Addl | Q9QYB6 | Q9D2M5 | Adducin-like protein 70 | Q8JZT6 | Add3
Q9R0Y0	1	0.0			Rbmxrt					10090	Rbmxrt | Hnrnpg | Hnrpg | Rbmxp1 | Q8C2U6 | Q9R0Y0 | Rbmx | Heterogeneous nuclear ribonucleoprotein G
Q8VEF4	1	0.0			Mitochondrial matrix protein P1					10090	Q3KQP2 | Mitochondrial matrix protein P1 | Q8C2C7 | P97602 | P19226 | P19227 | Q8VEF4 | Chaperonin 60 | Hsp60 | HSP-65 | 60 kDa chaperonin | Q3UIP0 | Heat shock protein 60 | Hspd1
Q99KS3	1	0.0			Ilf2					10090	Q3U083 | Q8CCY9 | Q99KS3 | Ilf2 | Nf45 | Q5RKG0 | Nuclear factor of activated T-cells 45 kDa
Q9D834	1	0.0			Microsomal glutathione S-transferase III					10090	Microsomal glutathione S-transferase III | Mgst3 | Q9D834 | Glutathione peroxidase MGST3 | Q3UXC5
Q9DBY6	1	0.0			PAI1 RNA-binding protein 1					10090	Q8BHS2 | SERPINE1 mRNA-binding protein 1 | Q9CSN0 | Q91WP3 | Q8BHU0 | Serbp1 | Q9DBY6 | PAI1 RNA-binding protein 1 | Pairbp1
Q99KQ6	1	0.0			Tacc2					10090	Q3TN02 | Q811U0 | Tacc2 | Q99KQ6 | Q8BQD4 | MNCb-3527
Q7TMM9	1	0.0			Tubb2a					10090	Tubb2a | Tubb2 | Q7TMM9
Q9D0N4	1	0.0			5mp1					10090	5mp1 | Q9D0N4 | Basic leucine zipper and W2 domain-containing protein 2 | Bzw2
B2RRB4	1	0.0			63-kDa cytoskeleton-linking membrane protein					10090	B2RRB4 | Q8R3F2 | 63-kDa cytoskeleton-linking membrane protein | Q8BTK8 | Ckap4
Q9CSA2	1	0.0			DEAH box protein 9					10090	O35931 | DEAH box protein 9 | Ddx9 | RNA helicase A | Q9CSA2 | Dhx9 | Nuclear DNA helicase II | Q5FWY1 | O54703 | Q6R5F7
Q8K1E9	1	0.0			Lactation elevated protein 1					10090	Q8K1E9 | Lactation elevated protein 1 | Lace1 | Q5EBH5 | Afg1l
Q3TIQ0	1	0.0			Eukaryotic translation initiation factor 2 subunit alpha					10090	Q3TIQ0 | Eukaryotic translation initiation factor 2 subunit alpha | Eif2s1 | Eif2a
Q3UPS6	1	0.0			3-methylcrotonyl-CoA carboxylase non-biotin-containing subunit					10090	3-methylcrotonyl-CoA carboxylase non-biotin-containing subunit | 3-methylcrotonyl-CoA:carbon dioxide ligase subunit beta | Mccc2 | 3-methylcrotonyl-CoA carboxylase 2 | Q3UPS6
Q9CT12	1	0.0			Eukaryotic translation initiation factor 2 subunit beta					10090	Q9CT12 | Eukaryotic translation initiation factor 2 subunit beta | Eif2s2 | Q9CSH6
O55131	1	0.0			CDC10 protein homolog					10090	Sept7 | O55131 | Septin7 | CDC10 protein homolog | Cdc10
O09042	1	0.0			Apolipoprotein A1					10090	Apolipoprotein A1 | Apoa1 | Q8BPD5 | O09042 | O08855
P80315	1	0.0			CCT-delta					10090	P80315 | CCT-delta | A45 | Cct4 | Cctd
Q8BKZ9	1	0.0			Dihydrolipoamide dehydrogenase-binding protein of pyruvate dehydrogenase complex					10090	Dihydrolipoamide dehydrogenase-binding protein of pyruvate dehydrogenase complex | Pdhx | Q8BKZ9 | Lipoyl-containing pyruvate dehydrogenase complex component X
Q99M42	1	0.0			Frg1					10090	Frg1 | FSHD region gene 1 protein | Q99M42
Q3TX86	1	0.0			Lrrc59					10090	Q3TLC7 | Q3TJ35 | Q3TWT9 | Lrrc59 | Q3TX86
E9Q105	1	0.0			RNA helicase p68					10090	RNA helicase p68 | DEAD box RNA helicase DEAD1 | Tnz2 | DEAD box protein 5 | Ddx5 | E9Q105
Q60697	1	0.0			Csma					10090	Q60697 | Csma | E9QQ50 | Q62162 | eIF3 p185 | eIF-3-theta | Centrosomin | Eif3a | p162 | eIF3 p180 | Eif3s10 | Eif3 | eIF3 p167 | Eukaryotic translation initiation factor 3 subunit 10
Q91WP2	1	0.0			Kiaa4192					10090	Q5SVX9 | Q91WP2 | Q6PFZ2 | Q8K0K7 | Q5SVX7 | Kiaa4192 | 2-oxoglutarate dehydrogenase, mitochondrial | Q3UDM7 | Q80Y57 | Q8K2Z3 | Alpha-ketoglutarate dehydrogenase | Ogdh | Q5DTI4 | Q8R3M2 | Thiamine diphosphate (ThDP)-dependent 2-oxoglutarate dehydrogenase
Q76MZ3	1	0.0			Ppp2r1a					10090	Ppp2r1a | PP2A subunit A isoform R1-alpha | PP2A subunit A isoform PR65-alpha | Q76MZ3
Q3THM9	1	0.0			Rps16					10090	Rps16 | Q3THM9
Q2NLC5	1	0.0			Mimt44					10090	Mimt44 | Timm44 | Tim44 | Q2NLC5
Q9DCI7	1	0.0			2,4-dienoyl-CoA reductase [NADPH]					10090	2,4-dienoyl-CoA reductase [NADPH] | Decr1 | Q9DCI7
Q9EQP2	1	0.0			Ehd4					10090	Ehd4 | PAST homolog 2 | Past2 | Q9EQP2
Q9CSZ0	1	0.0			PRP6 homolog					10090	Q8R3M8 | PRP6 homolog | Prpf6 | Q9CSZ0 | Q8CIK9 | U5 snRNP-associated 102 kDa protein | Q3ULJ7 | Q542P0 | Q99JN1
P70659	1	0.0			Erh					10090	P70659 | Q14259 | Erh
Q8BWW1	1	0.0			Eif3s7					10090	Eif3s7 | Eukaryotic translation initiation factor 3 subunit 7 | Q8BLH0 | Q8BWW1 | eIF3 p66 | Eif3d | eIF-3-zeta
Q3TWD3	1	0.0			Samm50					10090	Q3TIL3 | Samm50 | Q3TWD3 | Q3TTG7
Q91VJ6	1	0.0			Rpl3					10090	Rpl3 | O89071 | J1 protein | Q91VJ6
Q6P5D0	1	0.0			Dpysl2					10090	Q6P5D0 | Dpysl2 | Ulip2 | Crmp2 | Unc-33-like phosphoprotein 2
Q9CQE8	1	0.0			RTRAF					10090	Q9CQE8 | RTRAF
Q9JLE3	1	0.0			Erythrocyte adducin subunit alpha					10090	Q9JLE3 | Erythrocyte adducin subunit alpha | Add1
Q3TIW6	1	0.0			Scot					10090	Scot | Oxct1 | 3-oxoacid CoA-transferase 1 | Q3TIW6 | Somatic-type succinyl-CoA:3-oxoacid CoA-transferase | Succinyl-CoA:3-oxoacid CoA transferase | Q9CV92 | Oxct
Q61560	1	0.0			Tfrc					10090	Tfrc | Q61560 | Trfr
Q9QYJ0	1	0.0			Dnaja2					10090	Dnaja2 | mDj3 | Q9QYJ0
P70404	1	0.0			Idh3g					10090	Idh3g | NAD(+)-specific ICDH subunit gamma | Isocitric dehydrogenase subunit gamma | P70404
Q8CFP5	1	0.0			Map4					10090	Q3UUH5 | Q80YQ5 | Q8CFP5 | Q05BJ2 | Q7TPC6 | Q7TPD4 | Q3UY36 | Q3UIS2 | Map4 | Mtap4
Q9D004	1	0.0			Eef1g					10090	Q9D004 | Q9CRT5 | Eef1g | Q920C5 | Q9CSU3 | eEF-1B gamma
Q6A045	1	0.0			Thioredoxin domain-containing protein 4					10090	Thioredoxin domain-containing protein 4 | Q3TVI1 | Erp44 | Kiaa0573 | Q6A045 | Txndc4
Q505P4	1	0.0			Aco2					10090	Q3UDK9 | Q3ULG9 | Q3UNH7 | Q505P4 | Aco2 | Citrate hydro-lyase
