# Supplementary material for: Protein Networks Associated with Native Metabotropic Glutamate 1 Receptors (mGlu1) in the Mouse Cerebellum
Source: Cells. 2023 May 5;12(9):1325. doi: 10.3390/cells12091325 (PMC10177021; doi:10.3390/cells12091325)
Supplement: Supplementary file 1 [file cells-12-01325-s001.zip › Table S5.pdf]

Supplementary Table S5. Pathway enrichment analysis of network proteins.

Pathway source and name are displayed in columns 1 and 2; protein numbers and percentage are shown in columns 3 and 4; p-values and p values corrected for multiple comparisons are in columns 5 and 6; NCBI gene IDs are shown in column 7 and fold enrichment in column 8.

Category	Term	Count	%	PValue	FDR	Genes	Fold Enrichment
KEGG_PATHWAY	mmu04724:Glutamatergic synapse	12	27,27273	1,68E-13	1,66E-11	14816, 18752, 13385, 58234, 14681, 224997, 14688, 14812, 26558, 14810, 20512, 16438	27,3011378
REACTOME_PATHWAY	R-MMU-6794362~Protein-protein interactions at synapses	7	15,90909	2,36E-07	2,67E-05	14816, 13385, 58234, 224997, 14812, 26558, 14810	25,0450337
KEGG_PATHWAY	mmu04713:Circadian entrainment	7	15,90909	1,50E-06	3,72E-05	18752, 14681, 14688, 12323, 14812, 14810, 16438	18,3632653
REACTOME_PATHWAY	R-MMU-5578775~Ion homeostasis	6	13,63636	1,99E-06	1,28E-04	20541, 12323, 98660, 11931, 232975, 16438	27,2467949
KEGG_PATHWAY	mmu04720:Long-term potentiation	6	13,63636	4,63E-06	7,30E-05	14816, 18752, 12323, 14812, 14810, 16438	23,0226013
KEGG_PATHWAY	mmu05022:Pathways of neurodegeneration - multiple diseases	11	25	5,90E-06	7,30E-05	14816, 22190, 18752, 19122, 13385, 11739, 11949, 12323, 14812, 14810, 16438	6,00412496
REACTOME_PATHWAY	R-MMU-6794361~Neurexins and neuroligins	5	11,36364	7,00E-06	3,96E-04	14816, 13385, 58234, 224997, 26558	38,0869176
REACTOME_PATHWAY	R-MMU-112315~Transmission across Chemical Synapses	8	18,18182	1,34E-05	6,20E-04	18752, 22318, 13385, 14688,	9,49302066

						12323, 14812, 14810, 20512	
KEGG_PATHWAY	mmu04022:cGMP-PKG signaling pathway	7	15,90909	3,98E-05	3,94E-04	20541, 11739, 16531, 98660, 11931, 232975, 16438	10,4023121
KEGG_PATHWAY	mmu04730:Long-term depression	5	11,36364	7,13E-05	5,80E-04	14816, 18752, 14804, 14681, 16438	21,4238095
REACTOME_PATHWAY	R-MMU-438066~Unblocking of NMDA receptors, glutamate binding and activation	4	9,090909	9,33E-05	0,003013	13385, 12323, 14812, 14810	42,9343434
KEGG_PATHWAY	mmu05017:Spinocerebellar ataxia	6	13,63636	1,70E-04	0,001051	14816, 18752, 11739, 14812, 14810, 16438	10,9398176
KEGG_PATHWAY	mmu05017:Spinocerebellar ataxia	6	13,63636	1,70E-04	0,001051	14816, 18752, 11739, 14812, 14810, 16438	10,9398176
REACTOME_PATHWAY	R-MMU-112314~Neurotransmitter receptors and postsynaptic signal transmission	6	13,63636	2,15E-04	0,005713	18752, 13385, 14688, 12323, 14812, 14810	10,4178922
KEGG_PATHWAY	mmu04020:Calcium signaling pathway	7	15,90909	2,43E-04	0,001415	14816, 18752, 20541, 11739, 12323, 14810, 16438	7,49833333
REACTOME_PATHWAY	R-MMU-442755~Activation of NMDA receptors and postsynaptic events	4	9,090909	4,16E-04	0,009506	13385, 12323, 14812, 14810	26,2376543
KEGG_PATHWAY	mmu05020:Prion disease	7	15,90909	4,40E-04	0,002177	12391, 19122, 11739, 11949, 14812, 14810, 16438	6,71492537
REACTOME_PATHWAY	R-MMU-5673001~RAF/MAP kinase cascade	7	15,90909	6,19E-04	0,012718	22190, 240057, 13385, 54401, 12323, 14812, 14810	6,30905428

REACTOME_PATHWAY	R-MMU-5684996~MAPK1/MAPK3 signaling	7	15,90909	6,98E-04	0,013708	22190, 240057, 13385, 54401, 12323, 14812, 14810	6,1678068
REACTOME_PATHWAY	R-MMU-5683057~MAPK family signaling cascades	7	15,90909	0,001073	0,01865	22190, 240057, 13385, 54401, 12323, 14812, 14810	5,68031691
KEGG_PATHWAY	mmu04024:cAMP signaling pathway	6	13,63636	0,001303	0,005158	12323, 98660, 14812, 14810, 11931, 232975	7,01142857
REACTOME_PATHWAY	R-MMU-936837~Ion transport by P-type ATPases	4	9,090909	0,001374	0,022994	12323, 98660, 11931, 232975	17,4917695
KEGG_PATHWAY	mmu04723:Retrograde endocannabinoid signaling	5	11,36364	0,002221	0,008142	14816, 18752, 14681, 14688, 16438	8,68532819
REACTOME_PATHWAY	R-MMU-8849932~Synaptic adhesion-like molecules	3	6,818182	0,003293	0,051319	13385, 14812, 14810	33,734127
KEGG_PATHWAY	mmu04727:GABAergic synapse	4	9,090909	0,004485	0,013876	57138, 18752, 14681, 14688	11,5544141
REACTOME_PATHWAY	R-MMU-399719~Trafficking of AMPA receptors	3	6,818182	0,005419	0,074221	18752, 13385, 12323	26,2376543
REACTOME_PATHWAY	R-MMU-399721~Glutamate binding, activation of AMPA receptors and synaptic plasticity	3	6,818182	0,005419	0,074221	18752, 13385, 12323	26,2376543
KEGG_PATHWAY	mmu04014:Ras signaling pathway	5	11,36364	0,011374	0,030432	18752, 240057, 14688, 14812, 14810	5,46990881
