# Supplementary material for: Protein Networks Associated with Native Metabotropic Glutamate 1 Receptors (mGlu1) in the Mouse Cerebellum
Source: Cells. 2023 May 5;12(9):1325. doi: 10.3390/cells12091325 (PMC10177021; doi:10.3390/cells12091325)
Supplement: Supplementary file 1 [file cells-12-01325-s001.zip › Table S6.pdf]

Supplementary table S6: GO enrichment

FDR q-value is the p-value correction for multiple testing using the Benjamini and Hochberg method.

Enrichment (N, B, n, b) is defined as follows: N - is the total number of genes; B - is the total number of genes associated with a specific GO term

n - is the number of genes in the input list; b - is the number of genes in the intersection; Enrichment = (b/n) / (B/N)

GO Term	Description	P-value	FDR q-value	Enrichment	N	B	n	b	Genes
GO:0048169	regulation of long-term neuronal synaptic plasticity	3,59E-11	3,80E-08	53,62	10870	33	43	7	[Dlg4 - discs, large homolog 4 (drosophila), Shank3 - sh3/ankyrin domain gene 3, Grin1 - glutamate receptor, ionotropic, nmda1 (zeta 1), Syngap1 - synaptic ras gtpase activating protein 1 homolog (rat), Grin2b - glutamate receptor, ionotropic, nmda2b (epsilon 2), Fmr1 - fragile x mental retardation syndrome 1, Camk2b - calcium/calmodulin-dependent protein kinase ii, beta]
GO:0022898	regulation of transmembrane transporter activity	2,93E-10	2,55E-07	13,5	10870	206	43	11	[Prnp - prion protein, Dlg4 - discs, large homolog 4 (drosophila), Shank3 - sh3/ankyrin domain gene 3, Slc25a4 - solute carrier family 25 (mitochondrial carrier, adenine nucleotide translocator), member 4, Atp1b1 - atpase, na+/k+ transporting, beta 1 polypeptide, Htt - huntingtin, Ywhae - tyrosine 3-monooxygenase/tryptophan 5-monooxygenase activation protein, epsilon polypeptide, Atp1a2 - atpase, na+/k+ transporting, alpha 2 polypeptide, Vamp2 - vesicle-associated membrane protein 2, Cav3 - caveolin 3, Fmr1 - fragile x mental retardation syndrome 1]
GO:0051924	regulation of calcium ion transport	3,25E-10	2,68E-07	13,37	10870	208	43	11	[Prnp - prion protein, Grin1 - glutamate receptor, ionotropic, nmda1 (zeta 1), Atp1b1 - atpase, na+/k+ transporting, beta 1 polypeptide, Htt - huntingtin, Ywhae - tyrosine 3-monooxygenase/tryptophan 5-monooxygenase activation protein, epsilon polypeptide, Atp1a2 - atpase, na+/k+ transporting, alpha 2 polypeptide, Gnao1 - guanine nucleotide binding protein, alpha o, Itpr1 - inositol 1,4,5-trisphosphate receptor 1, Homer3 - homer homolog 3 (drosophila), Cav3 - caveolin 3, Fmr1 - fragile x mental retardation syndrome 1]
GO:0050806	positive regulation of synaptic transmission	4,02E-10	3,13E-07	16	10870	158	43	10	[Prkcg - protein kinase c, gamma, Dlg4 - discs, large homolog 4 (drosophila), Shank3 - sh3/ankyrin domain gene 3, Grin1 - glutamate receptor, ionotropic, nmda1 (zeta 1), Syt2 - synaptotagmin ii, Grin2b - glutamate receptor, ionotropic, nmda2b (epsilon 2), Vamp2 - vesicle-associated membrane protein 2, Fmr1 - fragile x mental retardation syndrome 1, Camk2b - calcium/calmodulin-dependent protein kinase ii, beta, Slc1a3 - solute carrier family 1 (glial high affinity glutamate transporter), member 3]
GO:0007215	glutamate receptor signaling pathway	2,73E-09	1,49E-06	45,96	10870	33	43	6	[Grin1 - glutamate receptor, ionotropic, nmda1 (zeta 1), Atp1a3 - atpase, na+/k+ transporting, alpha 3 polypeptide, Homer3 - homer homolog 3 (drosophila), Grin2b - glutamate receptor, ionotropic, nmda2b (epsilon 2), Fmr1 - fragile x mental retardation syndrome 1, Grm1 - glutamate receptor, metabotropic 1]
GO:0007626	locomotory behavior	3,14E-09	1,66E-06	12,96	10870	195	43	10	[Dlg4 - discs, large homolog 4 (drosophila), Cnp - 2',3'-cyclic nucleotide 3' phosphodiesterase, Shank3 - sh3/ankyrin domain gene 3, Atp1a3 - atpase, na+/k+ transporting, alpha 3 polypeptide, Grin1 - glutamate receptor, ionotropic, nmda1 (zeta 1), Kcnma1 - potassium large conductance calcium-activated channel, subfamily m, alpha member 1, Htt - huntingtin, Atp1a2 - atpase, na+/k+ transporting, alpha 2 polypeptide, Gnao1 - guanine nucleotide binding protein, alpha o, Grm1 - glutamate receptor, metabotropic 1]
GO:0051480	regulation of cytosolic calcium ion concentration	8,81E-09	3,84E-06	11,65	10870	217	43	10	[Dlg4 - discs, large homolog 4 (drosophila), Grin1 - glutamate receptor, ionotropic, nmda1 (zeta 1), Htt - huntingtin, Ywhae - tyrosine 3-monooxygenase/tryptophan 5-monooxygenase activation protein, epsilon polypeptide, Atp1a2 - atpase, na+/k+ transporting, alpha 2 polypeptide, Itpr1 - inositol 1,4,5-trisphosphate receptor 1, Grin2b - glutamate receptor, ionotropic, nmda2b (epsilon 2), Cav3 - caveolin 3, Gnb1 - guanine nucleotide binding protein (g protein), beta 1, Grm1 - glutamate receptor, metabotropic 1]
GO:0006874	cellular calcium ion homeostasis	1,01E-08	4,27E-06	9,66	10870	288	43	11	[Dlg4 - discs, large homolog 4 (drosophila), Grin1 - glutamate receptor, ionotropic, nmda1 (zeta 1), Atp1b1 - atpase, na+/k+ transporting, beta 1 polypeptide, Htt - huntingtin, Ywhae - tyrosine 3-monooxygenase/tryptophan 5-monooxygenase activation protein, epsilon polypeptide, Atp1a2 - atpase, na+/k+ transporting, alpha 2 polypeptide, Itpr1 - inositol 1,4,5-trisphosphate receptor 1, Grin2b - glutamate receptor, ionotropic, nmda2b (epsilon 2), Cav3 - caveolin 3, Gnb1 - guanine nucleotide binding protein (g protein), beta 1, Grm1 - glutamate receptor, metabotropic 1]
GO:0050807	regulation of synapse organization	1,81E-08	6,10E-06	10,8	10870	234	43	10	[Prnp - prion protein, Grid2 - glutamate receptor, ionotropic, delta 2, Dlg4 - discs, large homolog 4 (drosophila), Gpm6a - glycoprotein m6a, Shank3 - sh3/ankyrin domain gene 3, Ywhaz - tyrosine 3-monooxygenase/tryptophan 5-monooxygenase activation protein, zeta polypeptide, Grin1 - glutamate receptor, ionotropic, nmda1 (zeta 1), Grin2b - glutamate receptor, ionotropic, nmda2b (epsilon 2), Fmr1 - fragile x mental retardation syndrome 1, Camk2b - calcium/calmodulin-dependent protein kinase ii, beta]
GO:1901214	regulation of neuron death	4,52E-08	1,31E-05	8,35	10870	333	43	11	[Prnp - prion protein, Grid2 - glutamate receptor, ionotropic, delta 2, Prkcg - protein kinase c, gamma, Syngap1 - synaptic ras gtpase activating protein 1 homolog (rat), Grin1 - glutamate receptor, ionotropic, nmda1 (zeta 1), Agap2 - arfgap with gtpase domain, ankyrin repeat and ph domain 2, Kcnma1 - potassium large conductance calcium-activated channel, subfamily m, alpha member 1, Htt - huntingtin, Itpr1 - inositol 1,4,5-trisphosphate receptor 1, Grin2b - glutamate receptor, ionotropic, nmda2b (epsilon 2), Fmr1 - fragile x mental retardation syndrome 1]
GO:1901019	regulation of calcium ion transmembrane transporter activity	1,85E-07	4,28E-05	23,33	10870	65	43	6	[Atp1b1 - atpase, na+/k+ transporting, beta 1 polypeptide, Htt - huntingtin, Ywhae - tyrosine 3-monooxygenase/tryptophan 5-monooxygenase activation protein, epsilon polypeptide, Atp1a2 - atpase, na+/k+ transporting, alpha 2 polypeptide, Cav3 - caveolin 3, Fmr1 - fragile x mental retardation syndrome 1]
GO:0007611	learning or memory	2,63E-07	5,81E-05	9,68	10870	235	43	9	[Prnp - prion protein, Shank3 - sh3/ankyrin domain gene 3, Syngap1 - synaptic ras gtpase activating protein 1 homolog (rat), Atp1a3 - atpase, na+/k+ transporting, alpha 3 polypeptide, Grin1 - glutamate receptor, ionotropic, nmda1 (zeta 1), Htt - huntingtin, Atp1a2 - atpase, na+/k+ transporting, alpha 2 polypeptide, Grin2b - glutamate receptor, ionotropic, nmda2b (epsilon 2), Slc12a5 - solute carrier family 12, member 5]

GO:0007268	chemical synaptic transmission	3,45E-07	7,09E-05	11,56	10870	175	43	8	[Grid2 - glutamate receptor, ionotropic, delta 2, Prkcg - protein kinase c, gamma, Dlg4 - discs, large homolog 4 (drosophila), Grin1 - glutamate receptor, ionotropic, nmda1 (zeta 1), Kcnma1 - potassium large conductance calcium-activated channel, subfamily m, alpha member 1, Grin2b - glutamate receptor, ionotropic, nmda2b (epsilon 2), Slc12a5 - solute carrier family 12, member 5, Dlgap1 - discs, large (drosophila) homolog-associated protein 1]
GO:0008306	associative learning	4,75E-07	9,01E-05	19,96	10870	76	43	6	[Syngap1 - synaptic ras gtpase activating protein 1 homolog (rat), Grin1 - glutamate receptor, ionotropic, nmda1 (zeta 1), Atp1a3 - atpase, na+/k+ transporting, alpha 3 polypeptide, Htt - huntingtin, Atp1a2 - atpase, na+/k+ transporting, alpha 2 polypeptide, Grin2b - glutamate receptor, ionotropic, nmda2b (epsilon 2)]
GO:0007204	positive regulation of cytosolic calcium ion concentration	5,27E-07	9,88E-05	10,93	10870	185	43	8	[Dlg4 - discs, large homolog 4 (drosophila), Grin1 - glutamate receptor, ionotropic, nmda1 (zeta 1), Htt - huntingtin, Itpr1 - inositol 1,4,5-trisphosphate receptor 1, Grin2b - glutamate receptor, ionotropic, nmda2b (epsilon 2), Cav3 - caveolin 3, Gnb1 - guanine nucleotide binding protein (g protein), beta 1, Grm1 - glutamate receptor, metabotropic 1]
GO:0050890	cognition	5,98E-07	1,11E-04	8,78	10870	259	43	9	[Prnp - prion protein, Shank3 - sh3/ankyrin domain gene 3, Syngap1 - synaptic ras gtpase activating protein 1 homolog (rat), Atp1a3 - atpase, na+/k+ transporting, alpha 3 polypeptide, Grin1 - glutamate receptor, ionotropic, nmda1 (zeta 1), Htt - huntingtin, Atp1a2 - atpase, na+/k+ transporting, alpha 2 polypeptide, Grin2b - glutamate receptor, ionotropic, nmda2b (epsilon 2), Slc12a5 - solute carrier family 12, member 5]
GO:0006883	cellular sodium ion homeostasis	6,24E-07	1,14E-04	56,18	10870	18	43	4	[Atp1a3 - atpase, na+/k+ transporting, alpha 3 polypeptide, Atp1b1 - atpase, na+/k+ transporting, beta 1 polypeptide, Atp1a2 - atpase, na+/k+ transporting, alpha 2 polypeptide, Slc1a3 - solute carrier family 1 (glial high affinity glutamate transporter), member 3]
GO:0099537	trans-synaptic signaling	7,28E-07	1,30E-04	10,48	10870	193	43	8	[Grid2 - glutamate receptor, ionotropic, delta 2, Prkcg - protein kinase c, gamma, Dlg4 - discs, large homolog 4 (drosophila), Grin1 - glutamate receptor, ionotropic, nmda1 (zeta 1), Kcnma1 - potassium large conductance calcium-activated channel, subfamily m, alpha member 1, Grin2b - glutamate receptor, ionotropic, nmda2b (epsilon 2), Slc12a5 - solute carrier family 12, member 5, Dlgap1 - discs, large (drosophila) homolog-associated protein 1]
GO:0031644	regulation of neurological system process	7,82E-07	1,36E-04	13,3	10870	133	43	7	[Dlg4 - discs, large homolog 4 (drosophila), Shank3 - sh3/ankyrin domain gene 3, Grin1 - glutamate receptor, ionotropic, nmda1 (zeta 1), Homer3 - homer homolog 3 (drosophila), Fmr1 - fragile x mental retardation syndrome 1, Dlgap1 - discs, large (drosophila) homolog-associated protein 1, Grm1 - glutamate receptor, metabotropic 1]
GO:0034613	cellular protein localization	1,48E-06	2,33E-04	4,37	10870	810	43	14	[Syngap1 - synaptic ras gtpase activating protein 1 homolog (rat), Ywhaz - tyrosine 3-monooxygenase/tryptophan 5-monooxygenase activation protein, zeta polypeptide, Atp1b1 - atpase, na+/k+ transporting, beta 1 polypeptide, Grid2 - glutamate receptor, ionotropic, delta 2, Dlg4 - discs, large homolog 4 (drosophila), Shank3 - sh3/ankyrin domain gene 3, Grin1 - glutamate receptor, ionotropic, nmda1 (zeta 1), Htt - huntingtin, Ywhae - tyrosine 3-monooxygenase/tryptophan 5-monooxygenase activation protein, epsilon polypeptide, Grin2b - glutamate receptor, ionotropic, nmda2b (epsilon 2), Vamp2 - vesicle-associated membrane protein 2, Cav3 - caveolin 3, Ppp1r9b - protein phosphatase 1, regulatory subunit 9b, Tnik - traf2 and nck interacting kinase]
GO:0008104	protein localization	2,43E-06	3,59E-04	3,22	10870	1412	43	18	[Syngap1 - synaptic ras gtpase activating protein 1 homolog (rat), Ywhaz - tyrosine 3-monooxygenase/tryptophan 5-monooxygenase activation protein, zeta polypeptide, Atp1b1 - atpase, na+/k+ transporting, beta 1 polypeptide, Ywhab - tyrosine 3-monooxygenase/tryptophan 5-monooxygenase activation protein, beta polypeptide, Grid2 - glutamate receptor, ionotropic, delta 2, Dlg4 - discs, large homolog 4 (drosophila), Shank3 - sh3/ankyrin domain gene 3, Grin1 - glutamate receptor, ionotropic, nmda1 (zeta 1), Htt - huntingtin, Ywhaq - tyrosine 3-monooxygenase/tryptophan 5-monooxygenase activation protein, theta polypeptide, Ywhae - tyrosine 3-monooxygenase/tryptophan 5-monooxygenase activation protein, epsilon polypeptide, Grin2b - glutamate receptor, ionotropic, nmda2b (epsilon 2), Ywhag - tyrosine 3-monooxygenase/tryptophan 5-monooxygenase activation protein, gamma polypeptide, Vamp2 - vesicle-associated membrane protein 2, Cav3 - caveolin 3, Ppp1r9b - protein phosphatase 1, regulatory subunit 9b, Dlgap1 - discs, large (drosophila) homolog-associated protein 1, Tnik - traf2 and nck interacting kinase]
GO:0035235	ionotropic glutamate receptor signaling pathway	1,60E-05	1,95E-03	58,34	10870	13	43	3	[Grin1 - glutamate receptor, ionotropic, nmda1 (zeta 1), Atp1a3 - atpase, na+/k+ transporting, alpha 3 polypeptide, Grin2b - glutamate receptor, ionotropic, nmda2b (epsilon 2)]
GO:1900452	regulation of long-term synaptic depression	2,54E-05	2,91E-03	50,56	10870	15	43	3	[Grid2 - glutamate receptor, ionotropic, delta 2, Shank3 - sh3/ankyrin domain gene 3, Fmr1 - fragile x mental retardation syndrome 1]
GO:0010975	regulation of neuron projection development	3,22E-05	3,55E-03	4,75	10870	532	43	10	[Grid2 - glutamate receptor, ionotropic, delta 2, Dlg4 - discs, large homolog 4 (drosophila), Shank3 - sh3/ankyrin domain gene 3, Syngap1 - synaptic ras gtpase activating protein 1 homolog (rat), Grin1 - glutamate receptor, ionotropic, nmda1 (zeta 1), Syt2 - synaptotagmin ii, Itpr1 - inositol 1,4,5-trisphosphate receptor 1, Fmr1 - fragile x mental retardation syndrome 1, Camk2b - calcium/calmodulin-dependent protein kinase ii, beta, Tnik - traf2 and nck interacting kinase]
GO:0045664	regulation of neuron differentiation	4,20E-05	4,38E-03	4,15	10870	670	43	11	[Grid2 - glutamate receptor, ionotropic, delta 2, Dlg4 - discs, large homolog 4 (drosophila), Shank3 - sh3/ankyrin domain gene 3, Syngap1 - synaptic ras gtpase activating protein 1 homolog (rat), Grin1 - glutamate receptor, ionotropic, nmda1 (zeta 1), Syt2 - synaptotagmin ii, Itpr1 - inositol 1,4,5-trisphosphate receptor 1, Ywhag - tyrosine 3-monooxygenase/tryptophan 5-monooxygenase activation protein, gamma polypeptide, Fmr1 - fragile x mental retardation syndrome 1, Camk2b - calcium/calmodulin-dependent protein kinase ii, beta, Tnik - traf2 and nck interacting kinase]
GO:0043113	receptor clustering	4,28E-05	4,40E-03	20,22	10870	50	43	4	[Dlg4 - discs, large homolog 4 (drosophila), Shank3 - sh3/ankyrin domain gene 3, Syngap1 - synaptic ras gtpase activating protein 1 homolog (rat), Grin2b - glutamate receptor, ionotropic, nmda2b (epsilon 2)]
GO:1903279	regulation of calcium:sodium antiporter activity	4,57E-05	4,64E-03	168,53	10870	3	43	2	[Atp1b1 - atpase, na+/k+ transporting, beta 1 polypeptide, Atp1a2 - atpase, na+/k+ transporting, alpha 2 polypeptide]
GO:0099601	regulation of neurotransmitter receptor activity	5,01E-05	5,01E-03	19,45	10870	52	43	4	[Dlg4 - discs, large homolog 4 (drosophila), Shank3 - sh3/ankyrin domain gene 3, Homer3 - homer homolog 3 (drosophila), Dlgap1 - discs, large (drosophila) homolog-associated protein 1]
GO:0010469	regulation of signaling receptor activity	6,01E-05	5,71E-03	11,81	10870	107	43	5	[Dlg4 - discs, large homolog 4 (drosophila), Shank3 - sh3/ankyrin domain gene 3, Homer3 - homer homolog 3 (drosophila), Cav3 - caveolin 3, Dlgap1 - discs, large (drosophila) homolog-associated protein 1]

GO:0006836	neurotransmitter transport	6,29E-05	5,89E-03	11,7	10870	108	43	5	[Ywhaz - tyrosine 3-monooxygenase/tryptophan 5-monooxygenase activation protein, zeta polypeptide, Syt2 - synaptotagmin ii, Atp1a2 - atpase, na+/k+ transporting, alpha 2 polypeptide, Slc1a3 - solute carrier family 1 (glial high affinity glutamate transporter), member 3, Grm1 - glutamate receptor, metabotropic 1]
GO:0097688	glutamate receptor clustering	9,13E-05	8,14E-03	126,4	10870	4	43	2	[Dlg4 - discs, large homolog 4 (drosophila), Shank3 - sh3/ankyrin domain gene 3]
GO:0097113	AMPA glutamate receptor clustering	9,13E-05	8,29E-03	126,4	10870	4	43	2	[Dlg4 - discs, large homolog 4 (drosophila), Shank3 - sh3/ankyrin domain gene 3]
GO:0001504	neurotransmitter uptake	1,25E-04	1,09E-02	30,33	10870	25	43	3	[Atp1a2 - atpase, na+/k+ transporting, alpha 2 polypeptide, Slc1a3 - solute carrier family 1 (glial high affinity glutamate transporter), member 3, Grm1 - glutamate receptor, metabotropic 1]
GO:0051668	localization within membrane	1,31E-04	1,12E-02	10,03	10870	126	43	5	[Dlg4 - discs, large homolog 4 (drosophila), Shank3 - sh3/ankyrin domain gene 3, Syngap1 - synaptic ras gtpase activating protein 1 homolog (rat), Grin2b - glutamate receptor, ionotropic, nmda2b (epsilon 2), Vamp2 - vesicle-associated membrane protein 2]
GO:0051960	regulation of nervous system development	1,30E-04	1,13E-02	3,38	10870	898	43	12	[Grid2 - glutamate receptor, ionotropic, delta 2, Dlg4 - discs, large homolog 4 (drosophila), Shank3 - sh3/ankyrin domain gene 3, Syngap1 - synaptic ras gtpase activating protein 1 homolog (rat), Ywhaz - tyrosine 3-monooxygenase/tryptophan 5-monooxygenase activation protein, zeta polypeptide, Grin1 - glutamate receptor, ionotropic, nmda1 (zeta 1), Syt2 - synaptotagmin ii, Itpr1 - inositol 1,4,5-trisphosphate receptor 1, Ywhag - tyrosine 3-monooxygenase/tryptophan 5-monooxygenase activation protein, gamma polypeptide, Fmr1 - fragile x mental retardation syndrome 1, Camk2b - calcium/calmodulin-dependent protein kinase ii, beta, Tnik - traf2 and nck interacting kinase]
GO:0006605	protein targeting	1,51E-04	1,27E-02	9,72	10870	130	43	5	[Ywhaz - tyrosine 3-monooxygenase/tryptophan 5-monooxygenase activation protein, zeta polypeptide, Ywhaq - tyrosine 3-monooxygenase/tryptophan 5-monooxygenase activation protein, theta polypeptide, Ywhae - tyrosine 3-monooxygenase/tryptophan 5-monooxygenase activation protein, epsilon polypeptide, Ywhag - tyrosine 3-monooxygenase/tryptophan 5-monooxygenase activation protein, gamma polypeptide, Ywhab - tyrosine 3-monooxygenase/tryptophan 5-monooxygenase activation protein, beta polypeptide]
GO:0035304	regulation of protein dephosphorylation	1,80E-04	1,45E-02	14,04	10870	72	43	4	[Htt - huntingtin, Ywhae - tyrosine 3-monooxygenase/tryptophan 5-monooxygenase activation protein, epsilon polypeptide, Ppp1r9b - protein phosphatase 1, regulatory subunit 9b, Ywhab - tyrosine 3-monooxygenase/tryptophan 5-monooxygenase activation protein, beta polypeptide]
GO:0050848	regulation of calcium-mediated signaling	2,21E-04	1,73E-02	13,3	10870	76	43	4	[Prnp - prion protein, Htt - huntingtin, Homer3 - homer homolog 3 (drosophila), Itpr1 - inositol 1,4,5-trisphosphate receptor 1]
GO:0060079	excitatory postsynaptic potential	2,90E-04	2,16E-02	22,98	10870	33	43	3	[Grid2 - glutamate receptor, ionotropic, delta 2, Grin1 - glutamate receptor, ionotropic, nmda1 (zeta 1), Grin2b - glutamate receptor, ionotropic, nmda2b (epsilon 2)]
GO:0009966	regulation of signal transduction	3,26E-04	2,37E-02	2,18	10870	2204	43	19	[Prnp - prion protein, Fancd2 - fanconi anemia, complementation group d2, Syngap1 - synaptic ras gtpase activating protein 1 homolog (rat), Ywhaz - tyrosine 3-monooxygenase/tryptophan 5-monooxygenase activation protein, zeta polypeptide, Agap2 - arfgap with gtpase domain, ankyrin repeat and ph domain 2, Ywhab - tyrosine 3-monooxygenase/tryptophan 5-monooxygenase activation protein, beta polypeptide, Camk2b - calcium/calmodulin-dependent protein kinase ii, beta, Dlg4 - discs, large homolog 4 (drosophila), Shank3 - sh3/ankyrin domain gene 3, Grin1 - glutamate receptor, ionotropic, nmda1 (zeta 1), Htt - huntingtin, Itpr1 - inositol 1,4,5-trisphosphate receptor 1, Homer3 - homer homolog 3 (drosophila), Cav3 - caveolin 3, Grin2b - glutamate receptor, ionotropic, nmda2b (epsilon 2), Ppp1r9b - protein phosphatase 1, regulatory subunit 9b, Dlgap1 - discs, large (drosophila) homolog-associated protein 1, Tnik - traf2 and nck interacting kinase, Grm1 - glutamate receptor, metabotropic 1]
GO:0019722	calcium-mediated signaling	4,61E-04	3,22E-02	10,99	10870	92	43	4	[Prnp - prion protein, Atp1b1 - atpase, na+/k+ transporting, beta 1 polypeptide, Ppp1r9b - protein phosphatase 1, regulatory subunit 9b, Grm1 - glutamate receptor, metabotropic 1]
GO:0019220	regulation of phosphate metabolic process	5,64E-04	3,74E-02	2,57	10870	1377	43	14	[Prnp - prion protein, Syngap1 - synaptic ras gtpase activating protein 1 homolog (rat), Ywhaz - tyrosine 3-monooxygenase/tryptophan 5-monooxygenase activation protein, zeta polypeptide, Agap2 - arfgap with gtpase domain, ankyrin repeat and ph domain 2, Ywhab - tyrosine 3-monooxygenase/tryptophan 5-monooxygenase activation protein, beta polypeptide, Dlg4 - discs, large homolog 4 (drosophila), Htt - huntingtin, Ywhae - tyrosine 3-monooxygenase/tryptophan 5-monooxygenase activation protein, epsilon polypeptide, Grin2b - glutamate receptor, ionotropic, nmda2b (epsilon 2), Cav3 - caveolin 3, Ppp1r9b - protein phosphatase 1, regulatory subunit 9b, Fmr1 - fragile x mental retardation syndrome 1, Grm1 - glutamate receptor, metabotropic 1, Tnik - traf2 and nck interacting kinase]
GO:0048814	regulation of dendrite morphogenesis	7,06E-04	4,39E-02	9,82	10870	103	43	4	[Shank3 - sh3/ankyrin domain gene 3, Grin1 - glutamate receptor, ionotropic, nmda1 (zeta 1), Camk2b - calcium/calmodulin-dependent protein kinase ii, beta, Tnik - traf2 and nck interacting kinase]
GO:0000165	MAPK cascade	7,59E-04	4,59E-02	9,63	10870	105	43	4	[Shank3 - sh3/ankyrin domain gene 3, Ywhaz - tyrosine 3-monooxygenase/tryptophan 5-monooxygenase activation protein, zeta polypeptide, Ywhae - tyrosine 3-monooxygenase/tryptophan 5-monooxygenase activation protein, epsilon polypeptide, Tnik - traf2 and nck interacting kinase]
GO:0023014	signal transduction by protein phosphorylation	7,59E-04	4,61E-02	9,63	10870	105	43	4	[Shank3 - sh3/ankyrin domain gene 3, Ywhaz - tyrosine 3-monooxygenase/tryptophan 5-monooxygenase activation protein, zeta polypeptide, Ywhae - tyrosine 3-monooxygenase/tryptophan 5-monooxygenase activation protein, epsilon polypeptide, Tnik - traf2 and nck interacting kinase]
GO:0097106	postsynaptic density organization	9,84E-04	5,67E-02	42,13	10870	12	43	2	[Shank3 - sh3/ankyrin domain gene 3, Syngap1 - synaptic ras gtpase activating protein 1 homolog (rat)]
